# Natural inspired piperine-based ureas and amides as novel antitumor agents towards breast cancer

**DOI:** 10.1080/14756366.2021.1988944

**Published:** 2021-12-11

**Authors:** Diaaeldin M. Elimam, Abdullah A. Elgazar, Fardous F. El-Senduny, Ramadan A. El-Domany, Farid A Badria, Wagdy M. Eldehna

**Affiliations:** a Department of Pharmacognosy, Faculty of Pharmacy, Kafrelsheikh University, Kafrelsheikh, Egypt; b School of Chemistry and Biosciences, Faculty of Life Sciences, University of Bradford, Bradford, United Kingdom; c Department of Biochemistry, Faculty of Science, Mansoura University, Mansoura, Egypt; d Department of Microbiology and Immunology, Faculty of Pharmacy, Kafrelsheikh University, Kafrelsheikh, Egypt; e Department of Pharmacognosy, Faculty of Pharmacy, Mansoura University, Mansoura, Egypt; f Department of Pharmaceutical Chemistry, Faculty of Pharmacy, Kafrelsheikh University, Kafrelsheikh, Egypt

**Keywords:** Anticancer, piperic acid, VEGFR-2 inhibitors, molecular docking, triple negative breast cancer, natural products

## Abstract

In this work, the natural piperine moiety was utilised to develop two sets of piperine-based amides (**5a–i**) and ureas (**8a–y**) as potential anticancer agents. The anticancer action was assessed against triple negative breast cancer (TNBC) MDA-MB-231, ovarian A2780CP and hepatocellular HepG2 cancer cell lines. In particular, **8q** stood out as the most potent anti-proliferative analogue against TNBC MDA-MB-231 cells with IC_50_ equals 18.7 µM, which is better than that of piperine (IC_50_ = 47.8 µM) and 5-FU (IC_50_ = 38.5 µM). Furthermore, **8q** was investigated for its possible mechanism of action in MDA-MB-231 cells *via* Annexin V-FITC apoptosis assay and cell cycle analysis. Moreover, an in-silico analysis has proposed VEGFR-2 as a probable enzymatic target for piperine-based derivatives, and then has explored the binding interactions within VEGFR-2 active site (PDB:4ASD). Finally, an *in vitro* VEGFR-2 inhibition assay was performed to validate the *in silico* findings, where **8q** showed good VEGFR-2 inhibitory activity with IC_50_ = 231 nM.

## 1. Introduction

Breast cancer is one of the most challenging diseases that humanity is facing right now. In 2020, WHO estimated that out of 2.3 million women diagnosed by breast cancer, 685,000 deaths were reported globally^1^.

Triple-negative breast cancer (TNBC) stands out as the most serious type of breast cancer that represents about 10–15% of all breast cancer cases^2^. This type of breast cancer is characterised by a resistance to hormonal and anti-HER therapy since it lacks for oestrogenic and progesterone receptor (ER and PR), also it has low expression level of human epidermal growth factor receptor 2 (HER-2)^3^. Hence, surgery or chemotherapy are the only therapeutic choices against TNBC, yet they are associated with poor prognosis which means that the quest for development of more effective therapeutics is needed^4^.

**Figure 1.**
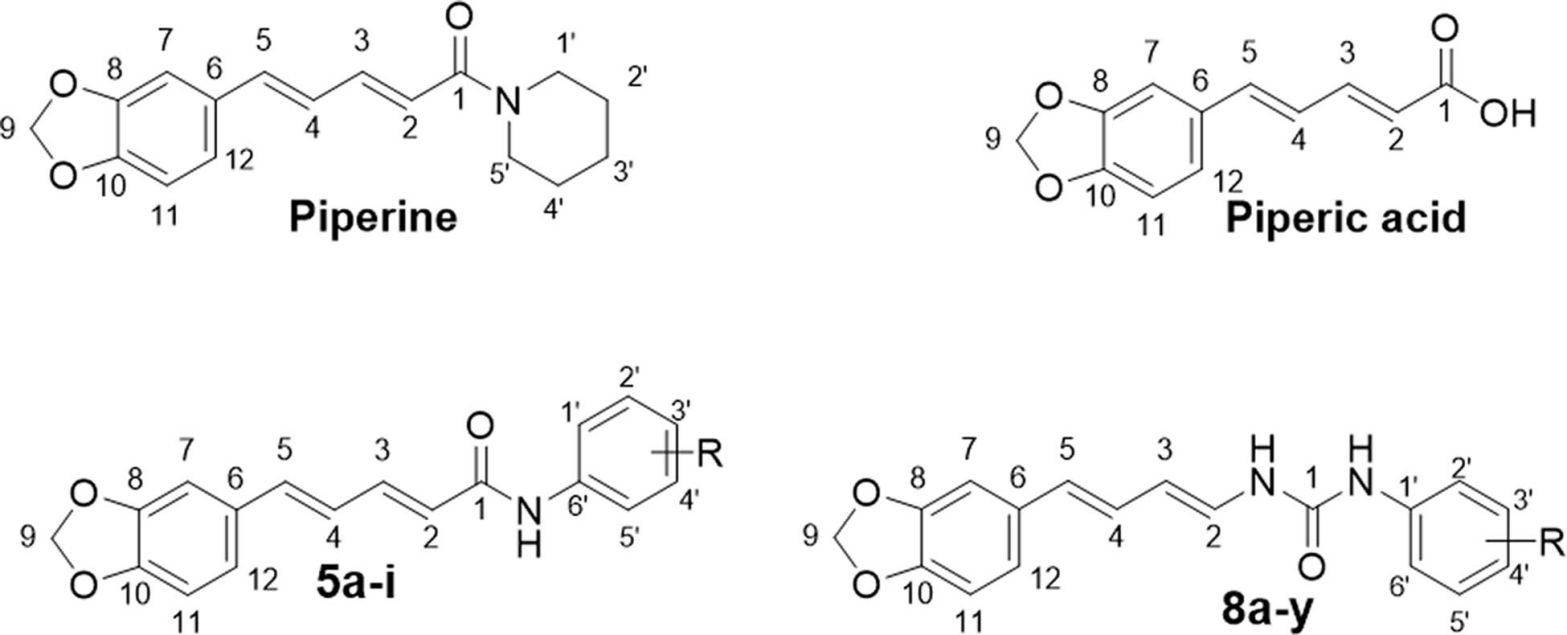
Chemical structure of piperine and piperic acid, as well as the target piperine-based amides (**5a–i**) and ureas (**8a–y**).

Natural products (NPs) have inspired humankind to discover new therapeutic agents for diverse diseases. This fact is coined by statistics showing that NPs represent most of known clinically used chemotherapeutic and antibiotics^5^. As one of the most interesting natural molecules, piperine (Figure 1) was reported to possess plethora of bioactivities such as anti-inflammatory, immunomodulatory, antioxidant, anti-diabetic and anticancer. Also, piperine was widely used to enhance the bioavailability of several drugs due to its ability to modulate p-glycoprotein and cytochrome P450 systems^6^^,^^7^. This could be explained by the versatility of the chemical structure of piperine which gives it the ability to interact with several pathway involved in pathogenesis of diseases.

Remarkably, Piperine was found to regulate several molecular targets associated with apoptosis in breast cancer such as caspase-3, p38 mitogen-activated protein kinase (p38 MAPK), Extracellular Signal-regulated kinase (ERK1/2), Activator Protein (AP-1), Nuclear Factor Kappa B (NF-κB) activation, Signal Transducer and Activator of Transcription (STAT-3) and Akt signalling pathways^7^. Moreover, it was found to reduce the expression of HER-2 genes and reduce the resistance of the cells to chemotherapeutic agents such as paclitaxel^8^^,^^9^. Interestingly, piperine was found to reduce the viability and motility of TNBC *in vitro* through activation of p21 and suppressing survival-promoting activating pathways, also it enhances the cytotoxic effect of gamma irradiation against TNBC in comparison to non-treated cells. These results were also observed in xenograft model in immune-compromised mice^10^. Collectively, these studies suggested piperine as a promising molecule for further modifications to develop efficient anti-cancer candidates.

Literature surveying hinted out that the chemical modification of piperine is a successful approach to enhance its biological activity and to afford opportunities in discovery of new anticancer therapies^11^. In this context, the alkaline hydrolysis of piperine to produce piperic acid (Figure 1) emerged as a common facile strategy to extend the synthetic accessibility of the molecule and furnish the chance to study structure activity relationships of the diverse piperine derivatives^12^^,^^13^.

Inspired by the above-mentioned findings, in the current study the natural piperine moiety was utilised to develop two sets of piperine-based amides (**5a–i**) and ureas (**8a–y**), (Figure 1). The anticancer actions for all piperine-based derivatives (**5a–i** and **8a–y**) will be assessed against three human cancer cell lines: TNBC (MDA-MB-231), ovarian (A2780CP) and hepatocellular (HepG2) following the protocol of MTT assay. Furthermore, the most efficient anti-proliferative counterpart will be investigated for its possible molecular mechanism of action in TNBC (MDA-MB-231) cell line *via* Annexin V-FITC apoptosis assay and cell cycle analysis. Moreover, an *in-silico* analysis has suggested VEGFR-2 as a potential enzymatic target for herein prepared piperine-based derivatives, and then has explored the binding interactions within VEGFR-2 active site. Finally, an *in vitro* VEGFR-2 inhibition assay will be performed to validate and confirm the *in-silico* findings.

## 2. Results and discussion

### 2.1. Chemistry

**Scheme 1.**
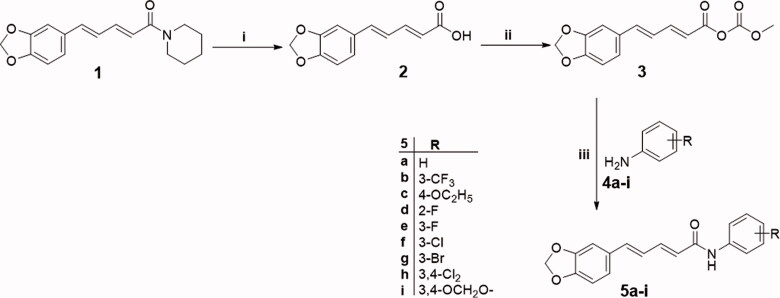
Synthesis of target piperine-based amides **5a–i**; Reagents and conditions: (i) 70% Ethanol/20% KOH/reflux 48 h, (ii) (a) Acetone/Triethylamine/cooling 0 °C in ice bath, (b) Methyl chloroformate cooling/stirring at 0 °C 30 min, (iii) Anhydrous acetone/stirring at 0–5 °C for 2–6 h.

**Scheme 2.**
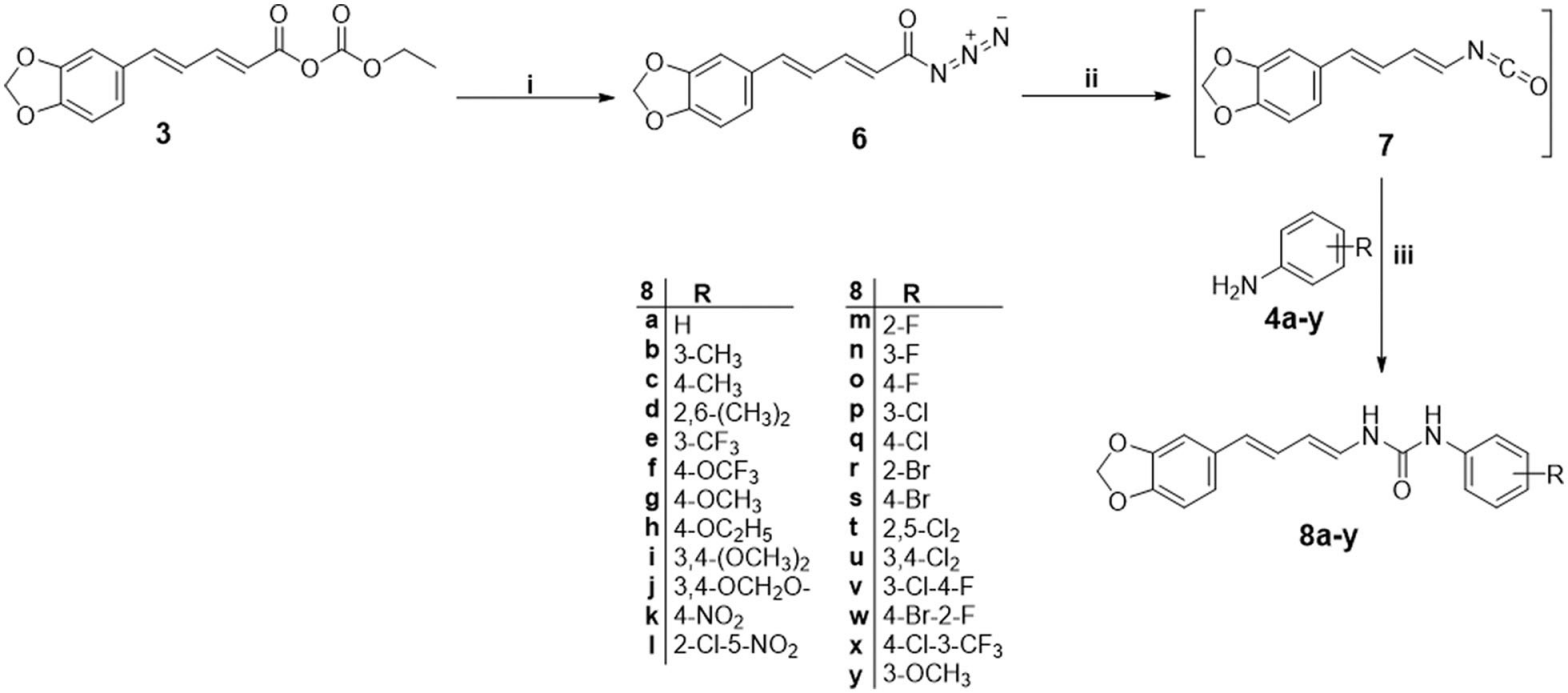
Synthesis of target piperine-based ureas **8a–y**; Reagents and conditions: (**i**) NaN_3_/acetone/stirring 0 °C/1 h, (**ii**) Anhydrous toluene/reflux 1 h, (**iii**) Anhydrous toluene/reflux 3 h.

The synthetic strategies adopted for preparation of target final piperine-based amides and ureas (**5a–i** and **8a–y**) were illustrated in Schemes 1 and 2.

In Scheme 1, piperine **1** was hydrolysed by 20% potassium hydroxide to afford piperic acid **2** which subsequently reacted with methyl chloroformate in the presence of triethylamine to furnish the key intermediate **3**. Thereafter, piperic acid mixed anhydride **3** was reacted with different anilines **4a–i** to produce piperine-based amides **5a–i** (Scheme 1).

Alternatively, mixed anhydride **3** was reacted with sodium azide to produce piperinoyl azide derivative **6**, which was refluxed in dry toluene to *in situ* produce the reactive piperinyl isocyanate **7** *via* Curtius rearrangement^14^. Aniline derivatives **(4a–y**) solubilised in hot toluene, were dropwise-added to the refluxing reaction mixture to immediately precipitate the more polar ureido derivatives (**8a–y)**, which were hot-filtered to furnish the pure product (Scheme 2).

The data obtained from spectral and elemental analyses were used to confirm the proposed structures of the synthesised compounds (**5a–i** and **8a–y**). The ^1^H NMR spectra of amides **5a–i** showed a singlet proton at (∼ δ_Η_ 10.20) demonstrating the amide proton; two multiplet protons and one singlet proton at the aromatic coupling region (δ_Η_ ∼ 8.25–6.80) hinting a tri-substituted aromatic ring; two olefinic multiplet protons overlapping the aromatic region, alongside one doublet benzylic methine proton at δ_Η_ 6.30–6.20 and one doublet up-field shifted olefinic proton (∼ δ_Η_ 6.30) suggesting a butadiene spacer between the aromatic moiety and the amide group (α, β unsaturated ketone); and two singlet protons at (∼ δ_Η_ 6.10) pointing to the methylene dioxy functionality. Regarding Scheme 2 (urea derivatives, **8a–y)**, the change of the splitting pattern of the α-olefinic proton into down-field shifted double doublet, with up field shifting of the β olefinic proton to reach (∼ δ_Η_ 5.8, double doublet) indicate the disruption of the amidic α, β unsaturated ketone system. Also, two signals at (∼δ_Η_ 8.90) of both a singlet proton and a doublet proton (coupled with adjacent methine proton, *J* ∼ 10.0 Hz) confirmed the presence of the ureido motif.

^13^C-NMR spectra (APT experiment), showed quaternary carbon signals at (∼ δ_C_ 165.0) which was assigned to the carbonyl carbon of the amide group, whereas, this value in urea derivatives **8** was up-field shifted (∼ δ_C_ 155.0) due to the bordering of the carbonyl carbon between the two nitrogens of the ureido moiety.

### 2.2. Biological evaluation

#### 2.2.1. Anti-proliferative activity towards cancer cell lines

All the synthesised piperine-based amides (**5a–i**) and ureas (**8a–y**) were screened for their potential *in vitro* anti-proliferative activity using the MTT assay as described by Skehan et al.^15^ The anti-proliferative action of all derivatives herein reported was first evaluated in an initial screening towards three cancer cell lines, namely breast (MDA-MB-231), ovarian (A2780CP) and hepatocellular (HepG2) cancer cell lines. This preliminary assessment of anticancer activity tested each compound in triplicate at 50 μM concentration, and the results were displayed as percentage cell viability caused by each tested derivative in Tables 1 and 2.

**Table 1.** *In vitro* anti-proliferative activity of amide-based derivatives **5a–i** against breast MDA-MB-231, ovarian A2780CP and hepatocellular HepG2 cancer cell lines

Compound	Cell viability (%)		
	at 50 μM		
	MDA-MB-231	A2780CP	HepG2
**5a**	72 ± 2	65 ± 1.01	52 ± 1.48
**5b**	162 ± 5	88 ± 1.08	67 ± 1.24
**5c**	24 ± 1.2	48 ± 0.46	33 ± 0.52
**5d**	79 ± 0.98	96 ± 1.8	52 ± 1.9
**5e**	60 ± 1.12	87 ± 1.7	65 ± 0.95
**5f**	87 ± 1.45	64 ± 1.5	54 ± 1.0
**5g**	166 ± 2.5	81 ± 1.85	63 ± 1.05
**5h**	103 ± 1.5	76 ± 1.2	49 ± 1.45
**5i**	71 ± 1.35	87 ± 1.95	46 ± 1.2

**Table 2.** *In vitro* anti-proliferative activity of urea-based derivatives **8a–y** against breast MDA-MB-231, ovarian A2780CP and hepatocellular HepG2 cancer cell lines

Compound	Cell viability (%)		
	at 50 μM		
	MDA-MB-231	A2780CP	HepG2
**8a**	55 ± 0.75	39 ± 1.14	28 ± 1.1
**8b**	22 ± 0.84	32 ± 1.75	29 ± 1.65
**8c**	38 ± 1.24	26 ± 0.15	25 ± 0.89
**8d**	62 ± 1.54	35 ± 1.3	52 ± 1.45
**8e**	29 ± 1.41	36 ± 0.58	31 ± 1.75
**8f**	69 ± 1.7	31 ± 0.41	34 ± 1.25
**8g**	38 ± 1.2	34 ± 0.78	49 ± 1.47
**8h**	71 ± 1.85	35 ± 1.15	46 ± 1.25
**8i**	49 ± 1.79	40 ± 1.05	56 ± 1.46
**8j**	54 ± 1.78	35 ± 0.75	45 ± 1.35
**8k**	26 ± 0.45	37 ± 1.45	51 ± 1.15
**8l**	46 ± 1.58	40 ± 1.98	54 ± 1.05
**8m**	20 ± 0.75	38 ± 1.78	36 ± 0.98
**8n**	69 ± 1.98	31 ± 0.89	43 ± 1.54
**8o**	45 ± 0.85	31 ± 1.24	27 ± 0.24
**8p**	23 ± 0.14	37 ± 1.28	28 ± 0.35
**8q**	8 ± 0.07	39 ± 1.15	31 ± 0.45
**8r**	8 ± 0.04	27 ± 0.58	34 ± 0.42
**8s**	25 ± 0.16	20 ± 0.49	50 ± 0.48
**8t**	15 ± 0.07	35 ± 1.35	36 ± 0.54
**8u**	33 ± 0.12	39 ± 1.24	39 ± 0.57
**8v**	41 ± 0.47	32 ± 0.98	17 ± 0.08
**8w**	18 ± 0.16	35 ± 0.79	39 ± 0.41
**8x**	19 ± 0.25	26 ± 0.85	30 ± 0.64
**8y**	50 ± 1.0	40 ± 1.42	33 ± 0.24

The tested piperine-based amides and ureas displayed diverse levels of growth inhibitory impact and elicited a distinctive manner of selectivity against the three examined cell lines. Concerning amide derivatives **5a–i**, exploration of the cell viability % displayed in Table 1 revealed that hepatocellular (HepG2) is the most susceptible examined cell line to the impact of the tested piperine-based amides (**5a–i**), with cell viability % range of 33–67 at 50 μM. In addition, the tested amides displayed cell viability % range of 48–96 and 24–87 towards A2780CP and MDA-MB-231 cell lines, respectively, (Table 1). In particular, *para* ethoxy-bearing amide **5c** efficiently inhibited the growth of the examined MDA-MB-231, A2780CP and HepG2 cells with cell viability % equal 24, 48 and 33, respectively.

On the other hand, breast (MDA-MB-231) emerged as the most susceptible examined cell line to the impact of the tested piperine-based ureas (**8a–y**), with cell viability % range of 8–69 at 50 μM as presented in Table 2. Notably, ureas (**8 b**, **8k**, **8 m**, **8p–t**, **8w–x**) exerted effective cell growth inhibition with cell viability % spanning between 8 and 26. Furthermore, the tested ureas exhibited cell viability % range of 20–40 and 17–56 towards A2780CP and HepG2 cell lines, respectively, (Table 2).

Consequently, the quantitative IC_50_ values for amide derivative (**5c**), urea derivatives (**8 b**, **8k**, **8 m**, **8p–t**, **8w–x**) and piperine against MDA-MB-231 cell line were determined and displayed in Table 3. 5-FU was utilised in this assay as a positive control. In particular, urea-bearing counterpart **8q** stood out as the most potent anti-proliferative analogue against MDA-MB-231 in this study with IC_50_ value equals 18.7 µM. In addition, ureas **8 b**, **8t** and **8w** exerted effective anti-proliferative activity (IC_50_ = 29.9, 37.09 and 36 µM, respectively) better than that of piperine (IC_50_ = 47.8 µM) and comparable to 5-FU (IC_50_ = 38.5 µM).

**Table 3.** IC_50_ for anti-proliferative activity of amide derivative (**5c**), urea derivatives (**8 b**, **8k**, **8 m**, **8p–t**, **8w–x**) and Piperine against breast MDA-MB-231 cancer cell line.

Compound	IC_50_ (μM)
	MDA-MB-231
**5c**	>100
**8b**	29.9 ± 0.56
**8k**	97 ± 1.23
**8m**	69.5 ± 1.17
**8p**	>100
**8q**	18.7 ± 0.14
**8r**	>100
**8s**	76.8 ± 1.25
**8t**	37.09 ± 1.42
**8w**	36 ± 1.17
**8x**	86 ± 1.98
**Piperine**	47.8 ± 0.25
**5-FU**	38.5 ± 2.1

#### 2.2.2. Cell cycle analysis

**Figure 2.**
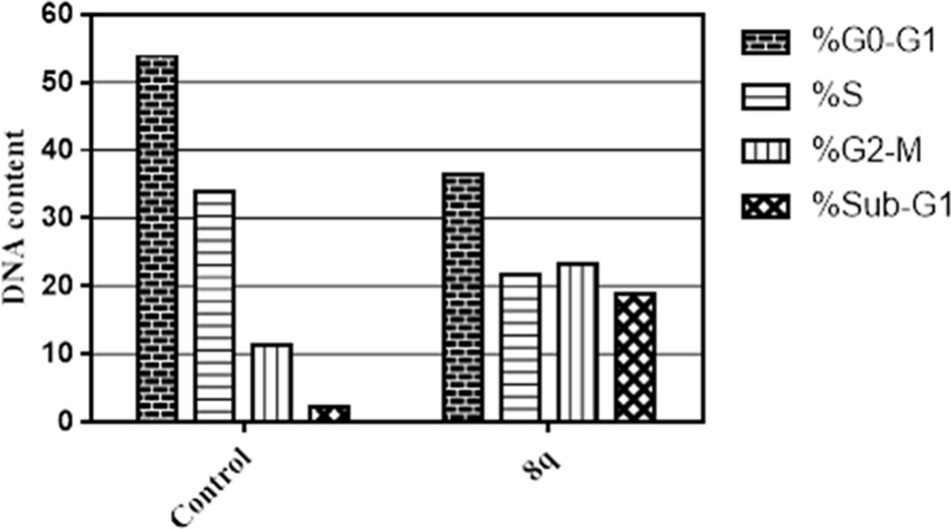
Impact of **8q** on the phases of cell cycle of MDA-MB-231 cells.

Cell cycle analysis using flow cytometric assay was preformed to obtain insights on the anti-proliferative activity of the most potent cytotoxic compound **8q** in this study. The treatment of MDA-MB-231 cells with **8q** at its IC_50_ led to a significant cell cycle arrest at G2-M phase and reduced cellular DNA content at S-phase. Moreover, a significant increase in the apoptotic cells at Sub-G1 phase by 9-fold compared to the control was observed as illustrated in Figure 2 and Supporting Information Table S1.

#### 2.2.3. Annexin V-FITC apoptosis assay

Annexin V-FITC/propidium iodide dual staining assay (AV/PI) was employed to assess if the anti-proliferative action of target urea-bearing derivative **8q** is in agree with the apoptosis induction within MDA-MB-231 indicated by the observed increase in sub-G1 population of treated cells (Figure 2).

**Figure 3.**
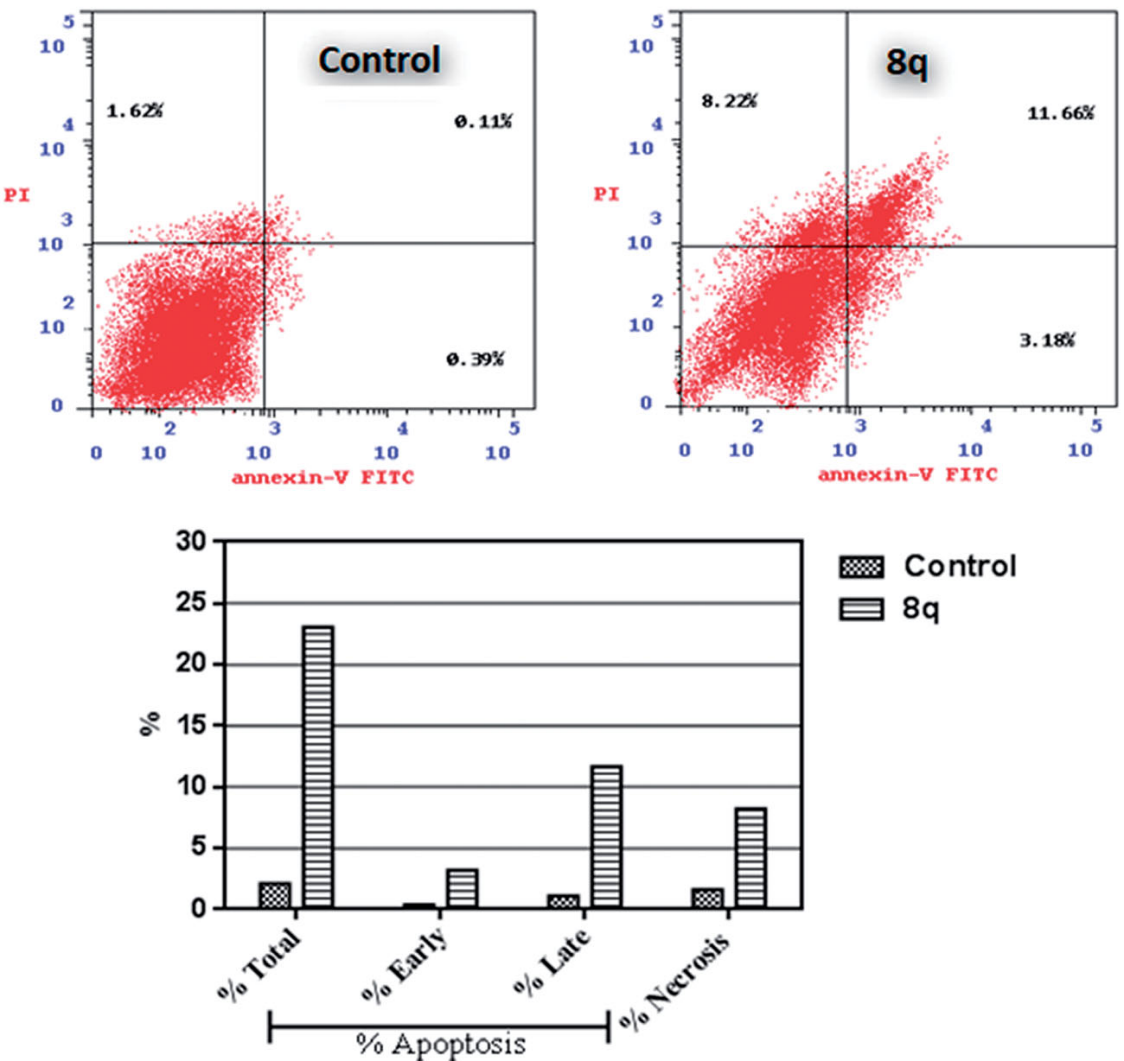
Impact of **8q** on the percentage of Ann V-FITC-positive staining in breast cancer MDA-MB-231 cells. The experiments were done in triplicates. The four quadrants identified as: LL: viable; LR: early apoptotic; UR: late apoptotic; UL: necrotic.

Compound **8q** was able to induce apoptosis in MDA-MB-231 as proved by the significant increase in the percent of the total apoptotic cells where the early apoptotic cells increased from 0.39% to 3.18% while cells in late apoptosis state increased from 0.11% to 11.66% which means that total apoptotic cells increased by 11-fold in comparison to the untreated cells as depicted in Figure 3. Interestingly, these results are in agree with a previous study reported that piperine could induce apoptosis in MDA-MB-231 cell line at 150 µM.^8^

### 2.3. In silico target prediction

Recently, target fishing has been used extensively to identify the plausible molecular targets of natural products and their derivatives^16–19^. As discussed above, compound **8q** showed the best anti-proliferative activity against MDA-MB-231 cells in the MTT assay, accordingly it was selected as representative counterpart to predict the potential molecular target of the prepared piperine-based derivatives.

In this study, the online Swiss TargetPrediction tool^20^ has been utilised to explore the potential targets. Among the top five predicted targets, as shown in Table S2, VEGFR-2 was suggested. It is worth highlighting that suggestion of VEGFR-2 as a possible target is perfectly matched with its known overexpression in TNBC cells^21^^,^^22^, as well as, with the reported anti-angiogenic activity of piperine and its analogous^23^^,^^24^. Consequently, a molecular docking analysis was further conducted to examine the possible binding modes and interactions of target piperine-based ureas within the vicinity of VEGFR-2 active site.

### 2.4. Vegfr-2 inhibitory activity

#### 2.4.1. Molecular docking analysis

**Figure 4.**
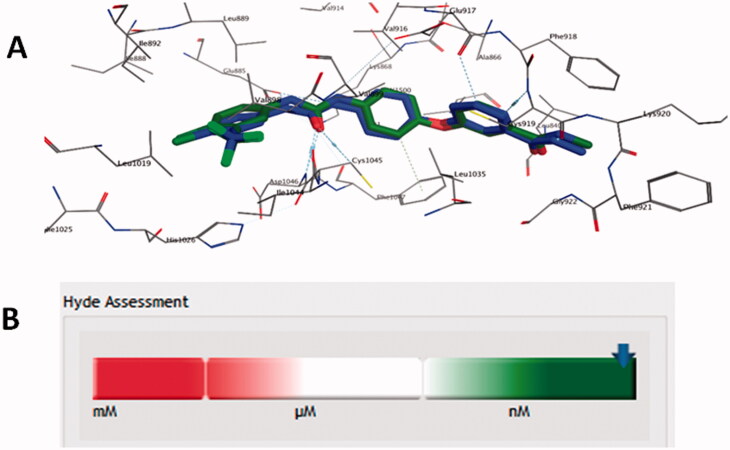
(A) Re-docked pose of the co-crystallized ligand (Green) aligned to the experimental pose (Blue) with RMSD = 0.8 Å in the active site of VEGFR-2 (PDB: 4ASD); (B) Hyde assessment showing the predicted *K*_i_ at low nanomolar range.

According to the suggestion of VEGFR-2 as a potential target for the synthesised derivatives, by Swiss TargetPrediction tool, docking analysis was exploited to support this assumption. Firstly, the docking protocol was validated by redocking of the co-crystallized ligand sorafenib within the VEGFR-2 binding site. Such redocking procedure revealed the ability of the co-crystallized ligand to reproduce the original experimental pose with RMSD = 0.8 Å. Also, rescoring with HYDE assessment predicted *K*_i_ of sorafenib in the low nanomolar range as in agree with its reported experimental value (Figure 4)^25^.

Thereafter, the software was used for molecular docking of derivatives that achieved comparable IC_50_ to 5-FU (ureas **8 b**, **8q**, **8t** and **8w**), and their energy score is presented in Table 4. Generally, the four examined compounds achieved acceptable binding interactions and energy scores. In particular, compound **8q** showed the best energy score (-28.34 kcal/mol) and was able to fit into the active site properly almost at the same position of sorafenib. Inspection of the top docking poses of compound **8q** revealed its ability to form two essential hydrogen bonds with the backbone NH of Asp1046 through the urea carbonyl oxygen, and with the carboxylate of Glu885 through the NH group of the urea linker. It is worthy to mention that these two amino acids are essential for the catalytic activity of VEGR-2^26^^,^^27^, which highlights the significance of incorporation of the urea linker in our target derivatives as an important structural feature for inhibition of VEGFR-2.

**Table 4.** Binding energy and Hyde assessment of top four cytotoxic piperine derivatives and sorafenib.

Compound	FlexXscore	HYDE score
**Sorafenib**	−35.6	−60
**8b**	−27.5	−28
**8q**	−28.34	−36
**8t**	−27.5	−27
**8w**	−27.3	−26

**Figure 5.**
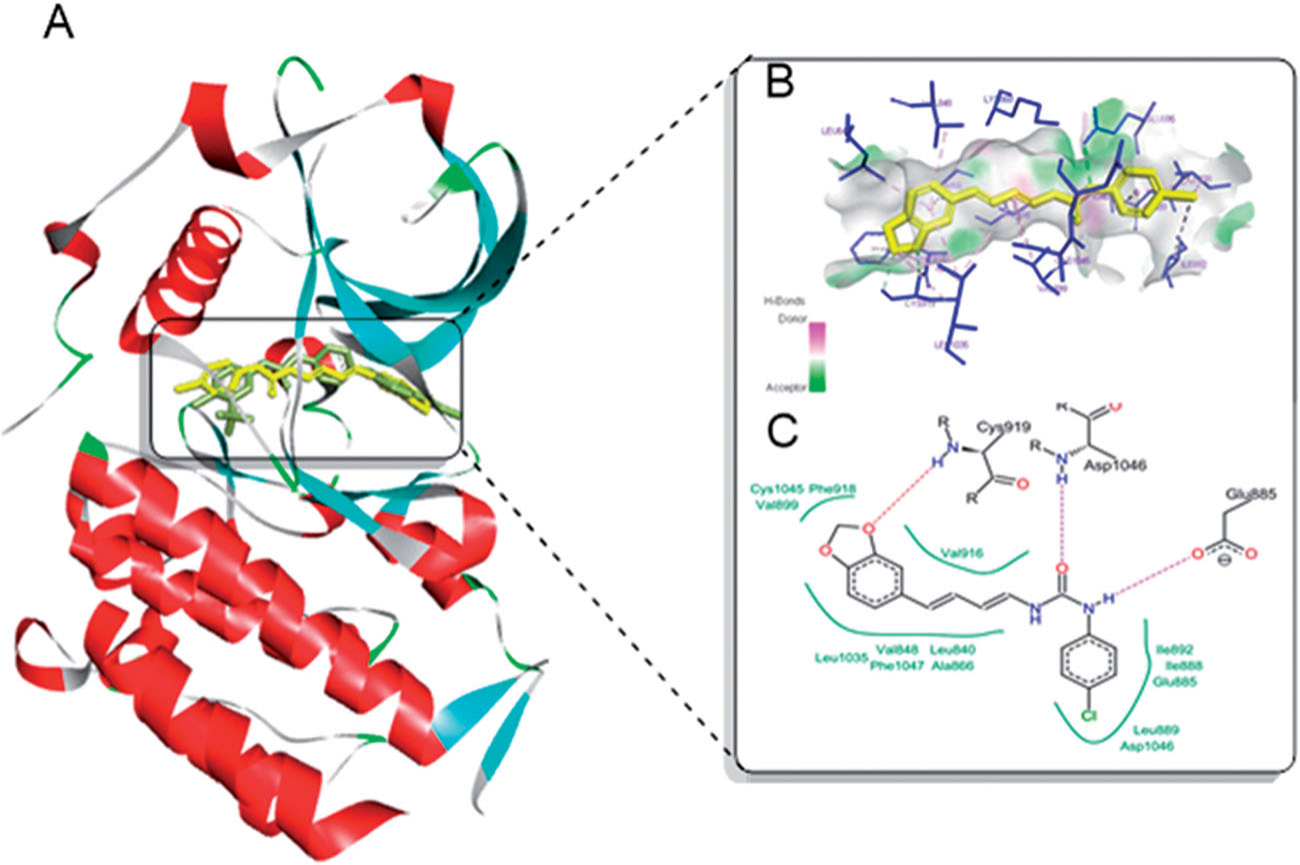
Molecular docking of compound **8q** in the active site of VEGFR-2 (PDB: 4ASD). (A) **8q** (Yellow) aligned to sorafenib (green) in the active site of VEGFR-2; (B) 3 D interactions of **8q** (yellow) with the active site of VEGFR-2 (blue); (C) 2 D interactions of **8q** with amino acids in the active site of VEGFR-2 where hydrogen bonds are showed as dashed lines and hydrophobic contacts are illustrated as spline segments.

Moreover, the conjugated diene in compound **8q** was involved in hydrophobic interactions with Leu840, Val848 and Phe1047. Also, it acted as a linker allowing the methylenedioxybenzene to form the third essential hydrogen bond with Cys919 in the hinge region of ATP binding site through oxygen of the methylenedioxy moiety, and to form hydrophobic interactions with Val899, Phe918 and Cys1045 as represented in Figure 5. In addition, the terminal *para*-chloro phenyl ring occupied the allosteric binding region and exerted good hydrophobic interactions with Ile592, Ile888, Glu885, Leu889 and Asp104. Furthermore, compounds **8w**, **8t** and **8 b** achieved comparable binding energy to **8q** (Table 4) and achieved good interactions within the vicinity of VEGFR-2 active site (Figure S1).

Notably, rescoring the docked poses using Hyde assessment showed that **8q** achieved the lowest binding energy and the highest HYDE score which might explain its better cytotoxic activity over other derivatives.

#### 2.4.2. In vitro VEGFR-2 inhibition assay

Since target fishing showed that compound **8q** might exert its action through inhibition of VEGFR-2, molecular docking was employed to obtain insights on the binding mode of the prepared compounds and revealed that compound **8q** was able to achieve the best binding energy among other examined compounds. Hence, an *in vitro* VEGFR-2 inhibition assay was performed, using sorafenib as positive control, to validate and confirm the *in-silico* findings. The results have been presented in Table 5 as IC_50_ values.

**Table 5.** IC_50_ values for inhibitory activity of piperine-based urea **8q** and Sorafenib against VEGFR-2.

Compound	IC_50_ (nM)^a^
	VEGFR-2
**8q**	231 ± 5
**Sorafenib**	59 ± 1.68

^a^IC_50_ values are the mean ± SD of three separate experiments..

Remarkably, **8q** was able to inhibit the VEGFR-2 with IC_50_ = 231 nM with about 4-fold decreased activity than sorafenib which inhibited the VEGFR-2 with IC_50_ = 59 nM (Table 5). This inhibition impact could be attributed to the presence of several common structural features required for VEGFR-2 inhibition in compound **8q**^26^. Also, as mentioned above piperine and its analogous were reported to possess anti-angiogenic activity in breast cancer^24^.

## 3. Conclusion

In the quest for finding new safe anticancer agents, two sets of thirty four piperine-based amides (**5a–i**) and ureas (**8a–y**) have been prepared, characterised successfully, and tested against three human cancer cell lines: TNBC (MDA-MB-231), ovarian (A2780CP) and hepatocellular (HepG2) following the protocol of MTT assay. Urea derivative **8q** showed significant cytotoxic activity (IC_50_ = 18.7 µM) against MDA-MB-231cells better than piperine (IC_50_ = 47.8 µM) and 5-FU (IC_50_ = 38.5 µM). The flow cytometry study for TNBC MDA-MB-231cells showed that its treatment with **8q** induced apoptosis at the late stage of cell division by causing cell arrest at G2-M phase and halted DNA synthesis by reducing S-phase population. Furthermore, enzyme inhibition assay showed that **8q** is a promising VEGFR-2 inhibitor with IC_50_ = 251 nM, which reveals one of the potential mechanisms responsible for its anticancer activity. Also, the observed inhibitory activity was explained in the light of molecular docking which demonstrated that **8q** was able to fulfil the requirement for binding properly in the active site of the enzyme by interacting with essential amino acid residues (Glu885, Cys919 and Asp1046) known to be necessary to achieve good VEGFR-2 inhibitory activity. Overall the gained results from this work sustained our strategy and granted us a robust opportunity for further optimisation of the natural piperine moiety to charge the therapeutic arsenal with efficient anticancer VEGFR-2 inhibitors.

## 4. Experimental

### 4.1. Chemistry

#### General

Melting points were measured with a FALC melting point apparatus and were uncorrected. The NMR spectra were recorded by Bruker spectrometer at 400 MHz ^13 ^C NMR spectra were run at 100 MHz in deuterated dimethyl sulfoxide (DMSO). Chemical shifts (*δH*) are reported relative to the solvent (DMSO). All coupling constant (*J*) values are given in hertz. Chemical shifts (*δC*) are reported relative to the solvent DMSO. Elemental analyses were carried out at the Regional Centre for Microbiology and Biotechnology, Al-Azhar University, Cairo, Egypt. Unless otherwise noted, all solvents and reagents were commercially available and used without further purification.

#### 4.1.1. Isolation of piperine 1

Dry powder of black pepper fruits (250 g) was packed in Soxhlet and extracted with isopropyl alcohol for 12 h. The extract was evaporated under vacuum using rotatory evaporator. The residual sticky mass was redissolved in 10% alcoholic KOH then cold distilled water was added slowly and left in the refrigerated overnight to give yellow precipitate which was collected by vacuum filtration and washed with cold diethyl-ether. Recrystallization from hot isopropanol was performed then from hot hexane/acetone mixture (3:2) to afford pure piperine as yellow crystalline needles.

#### 4.1.2. Hydrolysis of piperine to piperic acid 2

Piperine (10 g) was dissolved in 300 ml of 20% alcoholic KOH and refluxed with stirring for 72 h. Then, the solution rendered acidic using acetic acid and left on the refrigerator overnight to give yellow precipitate of piperic acid which was collected by vacuum filtration and recrystallized from hot ethyl acetate to give piperic acid (7.50 g, m.p 214–216 °C, yield 98%).

#### 4.1.3. Synthesis of mixed anhydride 3

Piperic acid (0.22 g, 1 mmol) was dissolved in dry acetone (3 ml), and then triethylamine was added dropwise until obtaining clear solution under cooling to 0 °C in an ice bath. Methyl chloroformate (MCF) (0.15 ml, 2 mmol) was added slowly with cooling (at 0 °C) and stirring for 30 min to form the mixed anhydride intermediate **3**, which used in the next step without further purification.

#### 4.1.4. General procedures for synthesis of piperine-based amides 5a–i

A solution of the appropriate aniline derivative (**4a–i**) in acetone was added dropwise to the previously prepared acetone solution of mixed anhydride **3**, under cooling at 0 °C in an ice bath. After complete addition, the reaction mixture was stirred for further 2–6 h at room temperature. The precipitated was filtered-off, washed with cold toluene (thrice) over vacuum, *n*-hexane, and recrystallized (in dark) from ethyl acetate to give pure crystalline product of piperine-based amides **5a–i** which stored in dark and dry conditions.

##### 4.1.4.1. (2E,4E)-5-(Benzo[d]1,3dioxol-5-yl)-N-phenylpenta-2,4-dienamide (5a)

White crystals (yield 61.8%), m.p0.192–193 °C (reported: 173–175 °C^28^) ^1^H NMR (DMSO-d_6_, 400 MHz) *δ ppm*: 10.10 (s, 1H, –CON**H**–), 7.71–7.69 (m, 2H, Aromatic), 7.37–7.31 (m, 4H, Aromatic), 7.08–6.93 (m, 5H, Aromatic and olefinic), 6.31 (d, *J* = 14.9 Hz, 1H, olefinic), 6.01 (s, 2H, C**H_2_**O_2_); ^13 ^C NMR (DMSO-d_6_, 100 MHz) *δ ppm*: 164.3, 148.4, 148.3, 141.5, 139.8, 139.4, 131.2, 129.2, 125.5, 124.8, 123.6, 123.4, 119.6, 108.9, 106.2, 101.8; Elemental Analysis for C_18_H_15_NO_3_: C, 73.71; H, 5.15; N, 4.78, Found C, 73.42; H, 5.18; N, 4.69.

##### 4.1.4.2. (2E,4E)-5-(Benzo[d]1,3dioxol-5-yl)-N-(3-(trifluoromethyl)phenyl)penta-2,4-dienamide (5b)

White crystals (yield 50%), m.p:122–124 °C; ^1^H NMR (DMSO-d_6_, 400 MHz) *δ ppm*: 10.50 (s, 1H, –CON**H**–), 8.20 (s, 1H, Aromatic), 7.87 (d, *J* = 8.3 Hz, 1H,aromatic), 7.57 (t, *J* = 8.0 Hz, 1H, olefinic), 7.42–7.34 (m, 3H, Aromatic), 7.09–6.94 (m, 4H, aromatic and olefinic), 6.30 (d, *J* = 14.9 Hz, 1H, Olefinic), 6.01 (s, 2H, C**H_2_**O_2_); ^13 ^C NMR (DMSO-d_6_, 100 MHz) *δ ppm*: 164.8, 148.5, 148.6, 145, 142.3, 140.6, 140, 131.2, 130.5, 130.1, 129.7, 125.9, 125.4, 125.3, 124.1, 123.9, 123.5, 123.1, 119.9, 119.9, 115.7, 115.6, 115.6, 115.5, 108.9, 106.2, 106.1, 101.8; Elemental Analysis for C_19_H_14_F_3_NO_3_: C, 63.16; H, 3.91; N, 3.88, Found C, 63.39; H, 3.94; N, 3.79.

##### 4.1.4.3. (2E,4E)-5-(Benzo[d]1,3dioxol-5-yl)-N-(4-ethoxyphenyl)penta-2,4-dienamide (5c)

White crystals (yield 53%), m.p: 194–195 °C; ^1^H NMR (DMSO-d_6_, 400 MHz) *δ ppm*: 10.00 (s, 1H, –CON**H**–), 7.60 (d, *J* = 2.0 Hz, 2H, Aromatic), 7.34–7.27 (q, 2H, Aromatic and olefinic), 7.04–6.88 (m, 7H, Aromatic and olefinic), 6.30 (d, 1H, olefinic), 7.60 (d, *J* = 8.6 Hz, 2H, Olefinic), 6.01 (s, 2H, C**H_2_**O_2_), 4.0 (q, *J* = 2.0 Hz, 2H, CH_2_) 1.32 (t, *J* = 6.9 Hz, 3H, CH_3_); ^13 ^C NMR (DMSO-d_6_, 100 MHz) *δ ppm*: 164.8, 148.5, 148.5, 145.1, 142.3, 140.6, 140.0, 131.1, 130.4, 130.0, 129.7, 125.9, 125.4, 125.3, 124.1, 123.9, 123.5, 123.1, 119.9, 119.9, 115.7, 115.6, 115.6, 115.6, 108.9, 106.2, 106.1, 101.8, 63.5 15.2; Elemental Analysis for C_20_H_19_NO_4_: C, 71.20; H, 5.68; N, 4.15, Found C, 70.83; H, 5.76; N, 4.11.

##### 4.1.4.4. (2E,4E)-5-(Benzo[d]1,3dioxol-5-yl)-N-(2-fluorophenyl)penta-2,4-dienamide (5d)

White crystals (yield 58%), m.p:182–184 °C; ^1^H NMR (DMSO-d_6_, 400 MHz) *δ ppm*: 9.94 (s, 1H, –CON**H**–), 8.06–8.02 (m, 1H, Aromatic), 7.38–7.13 (m, 4H, Aromatic and olefinic), 7.06–6.93 (m, 5H, Aromatic and olefinic), 6.50 (d, *J* = 15.0 Hz, 1H, olefinic) 6.07 (s, 2H, C**H_2_**O_2_); ^13 ^C NMR (DMSO-d_6_, 100 MHz) *δ ppm*: 164.2, 159.6, 157.2, 148.5, 148.4, 141.6, 139.5, 136.3, 136.3, 131.2, 125.5, 124.6, 123.5, 121.4, 121.3, 115.9, 115.6, 108.9, 106.2, 101.8; Elemental Analysis for C_18_H_14_FNO_3_: C, 69.45; H, 4.53, N, 4.50, Found C, 69.23; H, 4.48, N, 4.56.

##### 4.1.4.5. (2E,4E)-5-(Benzo[d]1,3dioxol-5-yl)-N-(3-fluorophenyl)penta-2,4-dienamide (5e)

White crystals (yield 60.%), m.p: 200–202 °C; ^1^H NMR (DMSO-d_6_, 400 MHz) *δ ppm*: 10.20 (s, 1H, –CON**H**–), 7.72 (dd, *J* = 8.7, 5.0 Hz, 2H, aromatic), 7.34 (dd, *J* = 14.9, 10.2 Hz, 1H, olefinic) 7.30 (s, 1H, aromatic), 7.17 (t, *J* = 8.3 Hz, 2H, aromatic), 7.12 − 6.71 (m, 4H, aromatic and olefinic), 6.29 (d, *J* = 14.8 Hz, 1H, olefinic), 6.10 (s, 2H, C**H_2_**O_2_); ^13 ^C NMR (DMSO-d_6_, 100 MHz) *δ ppm*: 166.3, 159.6, 157.2, 148.5, 148.4, 141.6, 139.5, 136.3, 136.3, 131.2, 125.5, 124.6, 123.5, 121.4, 121.3, 115.9, 115.6, 108.9, 106.2, 101.8; Elemental Analysis for C_18_H_14_FNO_3_: C, 69.45; H, 4.53; N, 4.50, Found C, 69.73; H, 4.58; N, 4.47.

##### 4.1.4.6. (2E,4E)-5-(Benzo[d]1,3dioxol-5-yl)-N-(3-chlorophenyl)penta-2,4-dienamide (5f)

White crystals (yield 55%), m.p:122–124 °C; ^1^H NMR (DMSO-d_6_, 400 MHz) *δ ppm*: 10.30 (s, 1H, –CON**H**–), 7.95 (d, *J* = 2.0 Hz, 1H, aromatic), 7.53 (d, *J* = 8.1 Hz, 1H, aromatic), 7.40–7.33 (m, 3H, aromatic and olefinic), 7.00–7.10 (m, 5H, aromatic and olefinic), 6.28 (d, *J* = 14.9 Hz, 1H, olefinic), 6.10 (s, 2H, C**H_2_**O_2_); ^13 ^C NMR (DMSO-d_6_, 100 MHz) *δ ppm*: 164.6, 148.5, 148.5, 142.2, 141.3, 139.9, 133.6, 131.2, 130.9, 125.4, 124.2, 123.5, 123.3, 119.0, 118.0, 108.9, 106.2, 101.8; Elemental Analysis for C_18_H_14_ClNO_3_: C, 65.96; H, 4.31; N, 4.27, Found C, 66.21; H, 4.35; N, 4.31.

##### 4.1.4.7. (2E,4E)-5-(Benzo[d]1,3dioxol-5-yl)-N-(3-bromophenyl)penta-2,4-dienamide (5 g)

White crystals (yield 48%), m.p:122–124 °C; ^1^H NMR (DMSO-d_6_, 400 MHz) *δ ppm*: 10.30 (s, 1H, –CON**H**–), 8.08 (d, *J* = 2.4 Hz, 1H, aromatic) 7.58 (d, *J* = 7.9 Hz, 1H, aromatic), 7.36–7.24 (m, 4H, aromatic and olefinic), 7.00–7.10 (m, 3H, aromatic and olefinic), 6.94 (d, *J* = 7.9 Hz, 1H, aromatic), 6.28 (d, *J* = 14.9 Hz, 1H, aromatic), 6.10 (s, 2H, C**H_2_**O_2_); ^13 ^C NMR (DMSO-d_6_, 100 MHz) *δ ppm*: 164.6, 148.5, 148.5, 142.2, 141.5, 139.9, 131.2, 131.2, 126.2, 125.4, 124.2, 123.57, 122.1, 121.9, 118.4, 108.9, 106.2, 101.8; Elemental Analysis for C_18_H_14_BrNO_3_: C, 58.08; H, 3.79; N, 3.76, Found C, 57.87; H, 3.83; N, 3.80.

##### 4.1.4.8. (2E,4E)-5-(Benzo[d]1,3dioxol-5-yl)-N-(3,4-dichlorophenyl)penta-2,4-dienamide (5 h)

White crystals (yield 50.1.%), m.p: 184–187 °C; ^1^H NMR (DMSO-d_6_, 400 MHz) *δ ppm*: 10.40 (s, 1H, –CON**H**–), 8.10 (s, 1H, aromatic) 7.60 (s, 2H, aromatic), 7.40–7.30 (m, 2H, aromatic and olefinic), 7.00–7.10 (m, 3H, aromatic and olefinic), 6.90–6.94 (d, *J* = 8.0 Hz, 1H, aromatic), 6.26 (d, *J* = 14.9 Hz, 1H, aromatic), 6.1 (s, 2H, C**H_2_**O_2_); ^13 ^C NMR (DMSO-d_6_, 100 MHz) *δ ppm*: 164.6, 148.5, 148.5, 142.2, 141.5, 139.9, 131.2, 131.2, 126.2, 125.4, 124.2, 123.6, 122.1, 121.9, 118.3, 108.9, 106.2, 101.8; Elemental Analysis for C_18_H_13_Cl_2_NO_3_: C, 59.69; H, 3.62; N, 3.87, Found C, 59.45; H, 3.66; N, 3.81.

##### 4.1.4.9. (2E,4E)-N,5-Bis(benzo[d]1,3dioxol-5-yl)penta-2,4-dienamide (5i)

White crystals (yield 53.7%), m.p: 231–233 °C (reported: 202–203 °C^29^) ^1^H NMR (DMSO-d_6_, 400 MHz) *δ ppm*: 10.07 (s, 1H, –CON**H**–), 7.42 (d, *J* = 22.5 Hz, 1H, olefinic), 7.32–7.27 (m, 2H, Aromatic), 7.07–6.87 (m, 5H, Aromatic and olefinic), 6.88 (d, *J* = 8.5 Hz, 1H, olefinic), 6.26 (d, *J* = 15.0 Hz, 1H, olefinic), 6.00 (s, 2H, C**H_2_**O_2_), 6.07 (s, 2H, C**H_2_**O_2_); ^13 ^C NMR (DMSO-d_6_, 100 MHz) *δ ppm*: 163.99, 148.45, 148.36, 147.51, 143.31, 141.24, 139.26, 134.35, 131.25, 125.57, 124.80, 123.42, 112.39, 108.96, 108.58, 106.18, 101.79, 101.71, 101.42; Elemental Analysis for C_19_H_15_NO_5_: C, 67.65; H, 4.48; N, 4.15, Found C, 67.88; H, 4.53; N, 4.18.

#### 4.1.5. Synthesis of piperinoyl azide 6

An aqueous solution of sodium azide (0.22 g, 3.3 mmol) was gradually added to the previously prepared acetone solution of mixed anhydride **3** under cooling at 0 °C in an ice bath. The reaction mixture was stirred for 1 h at 0 °C then poured on excess ice-cold brine. The produced precipitate was extracted with ethyl acetate (3 × 15 ml), dried over anhydrous Na_2_SO_4_ and vacuo-removed at room temperature to produce reddish-yellow solid of piperinoyl azide (**3**) (0.36 g, 85% yield), which utilised in the next reaction without further purification.

#### 4.1.6. General procedures for synthesis of piperine-based ureas 8a–y

The azide derivative **6** (0.49 g, 2 mmol) was heated under reflux in dry toluene (4 ml) for 1 h to furnish the isocyanate analogue **7** *via* subjugating to Curtius Rearrangement process. To the previous hot solution, an equimolar amount of the appropriate aniline derivative **4a–y** (2.1 mmol) was added. The mixture was refluxed for additional three hours. After cooling, the obtained solid was filtered, washed with diethyl ether, and recrystallized form dioxane to get piperine-based ureas **8a–y**.

##### 4.1.6.1. 1-((1E,3E)-4-(Benzo[d]1,3dioxol-5-yl)buta-1,3-dien-1-yl)-3-phenylurea (8a)

White crystals (yield 59%), m.p: 238–240 °C; ^1^H NMR (DMSO-d_6_, 400 MHz) *δ ppm*: 8.74 (d, *J* = 11.1 Hz, 2H–NH–CO), 7.45 (d, *J* = 8.0 Hz, 2H, aromatic), 7.28 (t, *J* = 7.8 Hz, 2H,aromatic), 7.10 (s, 1H, aromatic), 7.00 (dt, *J* = 14.8, 9.2 Hz, 2H, olefinic), 6.88–6.76 (m, 3H, aromatic), 6.28 (d, *J* = 15.6 Hz, 1H, olefinic), 6.00 (s, 2H, C**H_2_**O_2_), 5.86 (dd, *J* = 13.9, 10.8 Hz, 1H, olefinic); ^13 ^C NMR (DMSO-d_6_, 100 MHz) *δ ppm*: 153.7, 152.1, 148.2, 146.3, 137.3, 133.1, 131.3, 129.6, 128.6, 127.4, 126.3, 120.5, 118.8, 109.7, 108.8, 105.1, 101.3; Elemental Analysis C_18_H_16_N_2_O_3_: C, 70.12; H, 5.23; N, 9.09, Found C, 69.90; H, 5.24; N, 9.14.

##### 4.1.6.2. 1-((1E,3E)-4-(Benzo[d]1,3dioxol-5-yl)buta-1,3-dien-1-yl)-3-(m-tolyl)urea (8 b)

White crystals (yield 56%), m.p: 197–199 °C; ^1^H NMR (DMSO-d_6_, 400 MHz) *δ ppm*: 8.73 (d, J = 10.7 Hz, 1H, NH–CO), 8.65 (s, 1H, NH–CO), 7.33–7.07 (m, 4H, aromatic and olefinic), 7.00 (dd, *J* = 13.8, 10.8 Hz, 1H, olefinic), 6.81 (dq, *J* = 10.7, 5.3, 3.9 Hz, 4H, aromatic), 6.28 (d, *J* = 15.5 Hz, 1H, olefinic), 6.00 (s, 2H, C**H_2_**O_2_), 5.85 (dd, *J* = 13.9, 10.8 Hz, 1H, olefinic), 2.28 (s, 3H, CH_3_); ^13 ^C NMR (DMSO-d_6_, 100 MHz) *δ ppm*: 152, 148.2, 146.4, 139.8, 138.4, 133.1, 129.1, 128.5, 127.3, 126.4, 123.2, 120.6, 119.2, 115.9, 109.8, 108.8, 105.1, 101.3, 21.6; Elemental Analysis for C_19_H_18_N_2_O_3_: C, 70.79; H, 5.63; N, 8.69, Found C, 71.04; H, 5.67; N, 8.61.

##### 4.1.6.3. 1-((1E,3E)-4-(Benzo[d]1,3dioxol-5-yl)buta-1,3-dien-1-yl)-3-(p-tolyl)urea (8c)

White crystals (yield 58%), m.p: 233–235 °C; ^1^H NMR (DMSO-d_6_, 400 MHz) *δ ppm*: 8.73 (d, *J* = 10.7 Hz, 1H, NH–CO), 8.65 (s, 1H, NH–CO), 7.33–7.07 (m, 4H, aromatic and olefinic), 7.00 (dd, *J* = 13.8, 10.8 Hz, 1H), 6.81 (dq, *J* = 10.7, 5.3, 3.9 Hz, 4H, aromatic), 6.28 (d, *J* = 15.5 Hz, 1H, olefinic), 6.00 (s, 2H, C**H_2_**O_2_), 5.85 (dd, *J* = 13.9, 10.8 Hz, 1H, olefinic), 2.25 (s, 3H, CH_3_); ^13 ^C NMR (DMSO-d_6_, 100 MHz) *δ ppm*: 152.03, 148.22, 146.37, 139.81, 138.44, 133.08, 129.11, 128.50, 127.34, 126.36, 123.24, 120.57, 119.24, 115.94, 109.87, 108.80, 107.35, 105.09, 101.30, 21.69; Elemental Analysis for C_19_H_18_N_2_O_3_: C, 70.79; H, 5.63; N, 8.69, Found C, 70.89; H, 5.68; N, 8.63.

##### 4.1.6.4. 1-((1E,3E)-4-(Benzo[d]1,3dioxol-5-yl)buta-1,3-dien-1-yl)-3–(2,6-dimethylphenyl)urea (8d)

White crystals (yield 54%), m.p: 267–269 °C, ^1^H NMR (DMSO-d_6_, 400 MHz) *δ ppm*: 8.86 (s, 1H, NH–CO), 7.84 (s, 1H, NH–CO), 7.08 (brs, 4H, aromatic), 7.00 (dd, *J* = 13.9, 10.8 Hz, 1H, olefinic), 6.84 (d, *J* = 8.2 Hz, 1H, olefinic), 6.84–6.73 (m, 2H, Aromatic), 6.25 (d, *J* = 15.6 Hz, 1H, olefinic), 6.00 (s, 2H, C**H_2_**O_2_), 5.83 (dd, *J* = 13.9, 10.8 Hz, 1H, olefinic), 2.18 (s, 6H, 2 CH_3_); ^13 ^C NMR (DMSO-d_6_, 100 MHz) *δ ppm*: 152.8, 148.2, 146.3, 136.1, 135.6, 133.2, 129.6, 127.6, 127.2, 126.6, 125.7, 120.4, 109.2, 109.1, 105.1, 101.3, 18.6; Elemental Analysis for C_20_H_20_N_2_O_3_: C, 71.41; H, 5.99; N, 8.33, Found C, 71.62; H, 6.06; N, 8.25.

##### 4.1.6.5. 1-((1E,3E)-4-(Benzo[d]1,3dioxol-5-yl)buta-1,3-dien-1-yl)-3–(3-(trifluoromethyl)phenyl)urea (8e)

White crystals (yield 48.3%), m.p: 190–193 °C; ^1^H NMR (DMSO-d_6_, 400 MHz) *δ ppm*: 9.11 (s, 1H, NH–CO), 8.89 (d, *J* = 10.6 Hz, 1H, NH–CO), 7.98 (s, 1H, aromatic), 7.61 (d, *J* = 8.4 Hz, 1H, aromatic), 7.51 (t, *J* = 8.1 Hz, 1H, aromatic), 7.32 (d, *J* = 7.7 Hz, 1H, aromatic), 7.10 (s, 1H, aromatic), 7.00 (dd, *J* = 14.0, 10.2 Hz, 1H,olefinic), 6.93–6.76 (m, 3H,aromatic and olefinic), 6.30 (d, *J* = 15.6 Hz, 1H, olefinic), 6.00 (t, *J* = 1.8 Hz, 2H, C**H_2_**O_2_), 5.91 (t, *J* = 12.4 Hz, 1H, olefinic); ^13 ^C NMR (DMSO-d_6_, 100 MHz) *δ ppm*: 152.1, 148.2, 146.5, 140.8, 135.1, 124.7, 128.2, 116.7, 114.7, 108.8, 105.1, 101.3; Elemental Analysis for C_19_H_15_F_3_N_2_O_3_: C, 60.64; H, 4.02; N, 7.44, Found C, 60.47; H, 3.98; N, 7.80.

##### 4.1.6.6. 1-((1E,3E)-4-(Benzo[d]1,3dioxol-5-yl)buta-1,3-dien-1-yl)-3–(4-(trifluoromethoxy)phenyl)urea (8f)

White crystals (yield 46%), m.p: 237–239 °C; ^1^H NMR (DMSO-d_6_, 400 MHz) *δ ppm*: 8.95 (s, 1H, NH–CO), 8.81 (d, *J* = 10.6 Hz, 1H, NH–CO), 7.55 (dd, *J* = 9.0, 2.4 Hz, 2H, Aromatic), 7.29 (d, *J* = 8.5 Hz, 2H, aromatic), 7.09 (s, 1H, aromatic), 7.00 (dd, *J* = 13.6, 9.7 Hz, 1H, olefinic), 6.88–6.75 (m, 3H, aromatic and olefinic), 6.29 (d, *J* = 15.6 Hz, 1H, olefinic), 6.00 (brs, 2H, C**H_2_**O_2_), 5.88 (t, *J* = 12.4 Hz, 1H, olefinic); ^13 ^C NMR (DMSO-d_6_, 100 MHz) *δ ppm*: 152.0, 148.2, 146.4, 143.1, 139.2, 130.5, 124.5, 124.4, 117.7, 110.4, 108.8, 105.1, 101.3; Elemental Analysis for C_19_H_15_F_3_N_2_O_4_: C, 58.17; H, 3.85; N, 7.14, Found C, 57.86; H, 3.87; N, 7.10.

##### 4.1.6.7. 1-((1E,3E)-4-(Benzo[d]1,3dioxol-5-yl)buta-1,3-dien-1-yl)-3–(4-methoxyphenyl)urea (8g)

White crystals (yield 53.7%), m.p: 226–228 °C; ^1^H NMR (DMSO-d_6_, 400 MHz) *δ ppm*: 8.72 (d, *J* = 10.5 Hz, 2H, NH–CO), 7.23–7.12 (m, 2H, aromatic), 7.09 (s, 1H, aromatic), 7.05–6.90 (m, 2H, aromatic and olefinic), 6.89–6.74 (m, 3H, aromatic and olefinic), 6.56 (d, *J* = 8.3 Hz, 1H, olefinic), 6.28 (d, *J* = 15.5 Hz, 1H, olefinic), 6.00 (d, *J* = 2.3 Hz, 2H, C**H_2_**O_2_), 5.85 (t, *J* = 12.4 Hz, 1H, olefinic), 3.73 (s, 3H, OCH_3_); ^13 ^C NMR (DMSO-d_6_, 100 MHz) *δ ppm*: 155.1, 152.2, 148.2, 146.3, 133.1, 132.9, 128.8, 127.4, 126, 120.6, 120.5, 114.5, 109.5, 108.8, 105.1, 101.3, 55.6; Elemental Analysis for: C_19_H_18_N_2_O_4_: C, 67.45; H, 5.36; N, 8.28, Found C, 67.66; H, 5.41; N, 8.35.

##### 4.1.6.8. 1-((1E,3E)-4-(Benzo[d]1,3dioxol-5-yl)buta-1,3-dien-1-yl)-3–(4-ethoxyphenyl)urea (8h)

White crystals (yield 54%), m.p: 240–242 °C; ^1^H NMR (DMSO-d_6_, 400 MHz) *δ ppm*: 8.67 (d, J = 10.7 Hz, 1H, NH–CO), 8.53 (s, 1H, NH–CO), 7.34 (d, *J* = 8.4 Hz, 2H, aromatic), 7.09 (s, 1H, aromatic), 7.01 (dd, *J* = 13.9, 10.7 Hz, 1H, olefinic), 6.82 (td, *J* = 15.8, 14.6, 9.3 Hz, 5H, aromatic and aliphatic), 6.26 (d, *J* = 15.5 Hz, 1H, olefinic), 6.00 (s, 2H, C**H_2_**O_2_), 5.83 (dd, *J* = 13.9, 10.8 Hz, 1H, olefinic), 3.97 (q, *J* = 6.9 Hz, 2H, C**H_2_**CH_3_), 1.31 (t, *J* = 6.9 Hz, 3H, CH_2_C**H_3_**); ^13 ^C NMR (DMSO-d_6_, 100 MHz) *δ ppm*: 154.3, 153.7, 152.2, 148.2, 146.3, 133.1, 132.8, 128.8, 128.4, 127.4, 127.3, 126.3, 126.1, 121.4, 120.6, 120.5, 115.1, 114.9, 109.5, 109.1, 108.8, 108.7, 107.4, 106., 105.1, 102.3, 101.3, 63.6, 15.2; Elemental Analysis C_20_H_20_N_2_O_4_: C, 68.17; H, 5.72; N, 7.95, Found C, 67.04; H, 5.74; N, 8.02.

##### 4.1.6.9. 1-((1E,3E)-4-(Benzo[d]1,3dioxol-5-yl)buta-1,3-dien-1-yl)-3–(3,4-dimethoxyphenyl)urea (8i)

White crystals (yield 51%), m.p: 240–242 °C; ^1^H NMR (DMSO-d_6_, 400 MHz) *δ ppm*: 8.67 (d, J = 10.7 Hz, 1H, NH–CO), 8.57 (s, 1H, NH–CO), 7.16 (s, 1H, aromatic), 7.09 (s, 1H, aromatic), 7.00 (t, *J* = 12.2 Hz, 1H, olefinic), 6.83 (ddd, *J* = 27.8, 18.7, 10.1 Hz, 5H, aromatic and olefinic), 6.27 (d, *J* = 15.5 Hz, 1H, olefinic), 6.00 (s, 2H, C**H_2_**O_2_), 5.84 (t, *J* = 12.5 Hz, 1H, olefinic), 3.72, 3.74 (2 s, 6H, 2 OCH_3_); ^13 ^C NMR (DMSO-d_6_, 100 MHz) *δ ppm*: 152.2, 149.2, 148.2, 146.3, 144.6, 133.5, 133.1, 128.7, 127.4, 126.2, 120.5, 112.8, 110.8, 109.6, 108.8, 105.1, 104.5, 101.3, 56.3, 55.8; Elemental Analysis for C_20_H_20_N_2_O_5_: C, 65.21; H, 5.47; N, 7.60, Found C, 65.07; H, 5.51; N, 7.67.

##### 4.1.6.10. 1-(Benzo[d]1,3dioxol-5-yl)-3-((1E,3E)-4-(benzo[d]1,3dioxol-5-yl)buta-1,3-dien-1-yl)urea (8j)

White crystals (yield 49%), m.p: 230–233 °C; ^1^H NMR (DMSO-d_6_, 400 MHz) *δ ppm*: 8.69 (d, *J* = 10.8 Hz, 1H, NH–CO), 8.62 (s, 1H, NH–CO), 7.19 (d, *J* = 2.0 Hz, 1H, aromatic), 7.09 (s, 1H, aromatic), 6.99 (dd, *J* = 13.9, 10.7 Hz, 1H, olefinic), 6.88–6.72 (m, 5H, aromatic and olefinic), 6.27 (d, *J* = 15.6 Hz, 1H, olefinic), 5.99 (d, *J* = 8.9 Hz, 4H, C**H_2_**O_2_), 5.84 (dd, *J* = 13.9, 10.8 Hz, 1H, olefinic); ^13 ^C NMR (DMSO-d_6_, 100 MHz) *δ ppm*: 152.2, 148.2, 147.6, 146.4, 142.6, 134.3, 133.1, 128.6, 127.4, 126.3, 120.5, 111.6, 109.7, 108.8, 108.6, 105.1, 101.5, 101.3, 101.3; Elemental Analysis for C_19_H_16_N_2_O_5_: C, 64.77; H, 4.58; N, 7.95, Found C, 64.91; H, 4.62; N, 8.01.

##### 4.1.6.11. 1-((1E,3E)-4-(Benzo[d]1,3dioxol-5-yl)buta-1,3-dien-1-yl)-3–(4-nitrophenyl)urea (8k)

White crystals (yield 55%), m.p: 223–225 °C; ^1^H NMR (DMSO-d_6_, 400 MHz) *δ ppm*: 9.54 (s, 1H, NH–CO), 9.03 (d, *J* = 10.5 Hz, 1H, NH–CO), 8.20 (d, *J* = 9.4 Hz, 2H, aromatic), 7.70 (d, *J* = 8.8 Hz, 2H) 7.11 (s, 1H, aromatic), 7.00 (t, *J* = 11.8 Hz, 1H, olefinic), 6.91–6.78 (m, 3H, aromatic and olefinic), 6.32 (d, *J* = 15.6 Hz, 1H, olefinic), 6.01 (s, 2H, C**H_2_**O_2_), 5.98 (m, 1H, olefinic); ^13 ^C NMR (DMSO-d_6_, 100 MHz) *δ ppm*: 151.6, 148.2, 146.6, 146.5, 141.6, 132.9, 127.7, 127.3, 126.9, 125.6, 120.8, 118.1, 111.5, 108.8, 105.2, 101.4; Elemental Analysis for C_18_H_15_N_3_O_5_: C, 61.19; H, 4.28; N, 11.89, Found C, 61.33; H, 4.25; N, 11.96.

##### 4.1.6.12. 1-((1E,3E)-4-(Benzo[d]1,3dioxol-5-yl)buta-1,3-dien-1-yl)-3–(2-chloro-5-nitrophenyl)urea (8l)

White crystals (yield 46%), m.p: 216–218 °C; ^1^H NMR (DMSO-d_6_, 400 MHz) *δ ppm*: 9.64 (d, J = 10.4 Hz, 1H, NH–CO), 9.15 (s, 1H, NH–CO), 8.70 (s, 1H, aromatic), 8.00–7.73 (m, 2H, aromatic), 7.11 (s, 1H, aromatic), 6.98 (q, *J* = 14.8, 13.5 Hz, 1H, olefinic), 6.91–6.78 (m, 3H, aromatic and olefinic), 6.36 (d, *J* = 15.5 Hz, 1H, olefinic), 6.01 (s, 2H, C**H_2_**O_2_), 5.91 (t, *J* = 12.4 Hz, 1H, olefinic); ^13 ^C NMR (DMSO-d_6_, 100 MHz) *δ ppm*: 151.7, 148.3, 147, 146.6, 137.4, 132.8, 130.8, 128.2, 127.6, 127.3, 126.8, 126.6, 120.8, 117.8, 114.9, 108.8, 105.2, 101.4; Elemental Analysis for C_18_H_14_ClN_3_O_5_: C, 55.75; H, 3.64; N, 10.84, Found C, 55.89; H, 3.63; N, 10.92.

##### 4.1.6.13. 1-((1E,3E)-4-(Benzo[d]1,3dioxol-5-yl)buta-1,3-dien-1-yl)-3–(2-fluorophenyl)urea (8m)

White crystals (yield 55%), m.p: 230–233 °C; ^1^H NMR (DMSO-d_6_, 400 MHz) *δ ppm*: 9.08 (d, *J* = 10.5 Hz, 1H, NH–CO), 8.58 (s, 1H, NH–CO), 8.12 (t, *J* = 8.3 Hz, 1H,aromatic), 7.24 (t, *J* = 9.9 Hz, 1H, olefinic), 7.19–7.07 (m, 2H, aromatic), 7.06–6.94 (m, 2H, aromatic), 6.83 (q, *J* = 9.4 Hz, 3H, aromatic and olefinic), 6.31 (d, *J* = 15.5 Hz, 1H, olefinic), 6.0 (brs, 2H, C**H_2_**O_2_), 5.84 (dd, *J* = 13.8, 10.9 Hz, 1H, olefinic); ^13 ^C NMR (DMSO-d_6_, 100 MHz) *δ ppm*: 153.7, 151.3, 148.2, 146.5, 132.9, 127.9, 127.8, 127.7, 127.1, 126.8, 125, 125.1, 123.2, 123.3, 121.1, 120.7, 115.6, 115.4, 110.4, 108.8, 105.1, 101.3; Elemental Analysis for C_18_H_15_FN_2_O_3_: C, 66.25; H, 4.63; N, 8.58, Found C, 65.97; H, 4.66; N, 8.49.

##### 4.1.6.14. 1-((1E,3E)-4-(Benzo[d]1,3dioxol-5-yl)buta-1,3-dien-1-yl)-3–(3-fluorophenyl)urea (8n)

White crystals (yield 55%), m.p: 235–237 °C; ^1^H NMR (DMSO-d_6_, 400 MHz) *δ ppm*: 8.75 (d, *J* = 11.9 Hz, 2H, NH–CO), 7.51–7.41 (m, 2H, aromatic), 7.12 (dd, *J* = 15.1, 6.7 Hz, 3H, aromatic), 7.00 (dd, *J* = 13.7, 10.8 Hz, 1H, olefinic), 6.86–6.78 (m, 3H, aromatic and olefinic), 6.28 (d, *J* = 15.5 Hz, 1H, olefinic), 6.00 (d, *J* = 2.1 Hz, 2H, C**H_2_**O_2_), 5.86 (dd, *J* = 14.0, 10.8 Hz, 1H, olefinic); ^13 ^C NMR (DMSO-d_6_, 100 MHz) *δ ppm*: 157.9, 152.2, 148.2, 146.4, 136.3, 133.1, 128.5, 127.3, 126.4, 120.5, 115.7, 110.0, 108.8, 101.3; Elemental Analysis for C_18_H_15_FN_2_O_3_: C, 66.25; H, 4.63; N, 8.58, Found C, 66.08; H, 4.67; N, 8.61.

##### 4.1.6.15. 1-((1E,3E)-4-(Benzo[d]1,3dioxol-5-yl)buta-1,3-dien-1-yl)-3–(4-fluorophenyl)urea (8o)

White crystals (yield 53%), m.p: 234–237 °C; ^1^H NMR (DMSO-d_6_, 400 MHz) *δ ppm*: 8.74 (d, *J* = 11.5 Hz, 2H, NH–CO), 7.52–7.40 (m, 2H, aromatic), 7.12 (ddd, *J* = 14.4, 6.1, 1.9 Hz, 3H, aromatic), 7.00 (dd, *J* = 14.0, 10.7 Hz, 1H, olefinic), 6.89–6.75 (m, 3H, aromatic and olefinic), 6.28 (d, *J* = 15.6 Hz, 1H, olefinic), 6.00 (brs, 2H, C**H_2_**O_2_), 5.86 (dd, *J* = 13.9, 10.7 Hz, 1H, olefinic); ^13 ^C NMR (DMSO-d_6_, 100 MHz) *δ ppm*: 159.1, 156.7, 152.2, 148.2, 146.4, 136.3, 136.3, 133.1 128.5, 127.3, 126.4, 120.6, 120.6, 120.5, 115.8, 115.6, 110, 108.8, 105.1, 101.3; Elemental Analysis for C_18_H_15_FN_2_O_3_: C, 66.25; H, 4.63; N, 8.58, Found C, 66.17; H, 4.59; N, 8.64.

##### 4.1.6.16. 1-((1E,3E)-4-(Benzo[d]1,3dioxol-5-yl)buta-1,3-dien-1-yl)-3–(3-chlorophenyl)urea (8p)

White crystals (yield 53%), m.p: 212–214 °C; ^1^H NMR (DMSO-d_6_, 400 MHz) *δ ppm*: 8.96 (s, 1H, NH–CO), 8.85 (d, *J* = 10.7 Hz, 1H, NH–CO), 7.70 (s, 1H, aromatic), 7.29 (d, *J* = 6.5 Hz, 2H, aromatic and olefinic), 7.10 (s, 1H, aromatic), 7.08–6.93 (m, 2H, olefinic), 6.88–6.76 (m, 3H, aromatic), 6.29 (d, *J* = 15.6 Hz, 1H, olefinic), 6.00 (brs, 2H, CH2O2), 5.89 (dd, *J* = 13.9, 10.8 Hz, 1H, olefinic); ^13 ^C NMR (DMSO-d_6_, 100 MHz) *δ ppm*: 152, 148.3, 146.4, 141.5, 133.7, 133, 130.9, 128.2, 127.3, 127.2, 126.8, 122.1, 120.6, 118.1, 117.2, 110.6, 108.8, 105.1, 101.3; Elemental Analysis for C_18_H_15_ClN_2_O_3_: C, 63.07; H, 4.41; N, 8.17, Found C, 62.83; H, 4.45; N, 8.11.

##### 4.1.6.17. 1-((1E,3E)-4-(Benzo[d]1,3dioxol-5-yl)buta-1,3-dien-1-yl)-3–(4-chlorophenyl)urea (8q)

White crystals (yield 47%), m.p: 243–245 °C; ^1^H NMR (DMSO-d_6_, 400 MHz) *δ ppm*: 8.89 (s, 1H, NH–CO), 8.79 (d, *J* = 10.7 Hz, 1H, NH–CO), 7.48 (d, *J* = 8.5 Hz, 2H, aromatic), 7.33 (d, *J* = 8.4 Hz, 2H, aromatic), 7.09 (s, 1H, aromatic), 7.00 (dd, *J* = 13.9, 10.7 Hz, 1H, olefinic), 6.93–6.75 (m, 3H aromatic and olefinic), 6.29 (d, *J* = 15.6 Hz, 1H, olefinic), 6.00 (s, 2H, C**H_2_**O_2_), 5.87 (dd, *J* = 13.9, 10.8 Hz, 1H, olefinic); ^13 ^C NMR (DMSO-d_6_, 100 MHz) *δ ppm*: 152.0, 148.2, 146.4, 138.9, 133.0, 129.1, 128.3, 127.2, 126.6, 126, 120.6, 120.28, 110.3, 108.8, 105.1, 101.3; Elemental Analysis for C_18_H_15_ClN_2_O_3_: C, 63.07; H, 4.41; N, 8.17, Found C, 63.24; H, 4.37; N, 8.13.

##### 4.1.6.18. 1-((1E,3E)-4-(Benzo[d]1,3dioxol-5-yl)buta-1,3-dien-1-yl)-3–(2-bromophenyl)urea (8r)

White crystals (yield 48%), m.p: 234–236 °C; ^1^H NMR (DMSO-d_6_, 400 MHz) *δ ppm*: 9.54 (d, *J* = 9.9 Hz, 1H, NH–CO), 8.14 (s, 1H, NH–CO), 8.05 (d, *J* = 8.3 Hz, 1H, aromatic), 7.62 (d, *J* = 8.0 Hz, 1H, aromatic), 7.32 (d, J = 15.9 Hz, 1H, olefinic), 7.10 (s, 1H, aromatic), 7.00 (q, *J* = 8.1 Hz, 2H, aromatic), 6.84 (dd, *J* = 8.1, 5.5 Hz, 3H, aromatic and olefinic), 6.32 (d, *J* = 15.7 Hz, 1H, olefinic), 6.00 (d, *J* = 2.3 Hz, 2H, C**H_2_**O_2_), 5.85 (t, *J* = 12.4 Hz, 1H, olefinic); ^13 ^C NMR (DMSO-d_6_, 100 MHz) *δ ppm*: 151.8, 148.2, 146.5, 137.3, 137.2, 132.9, 129.3, 128.1, 127.9, 127, 126.9, 124.8, 122.7, 113.4, 110.5, 110.5, 108.6, 101.3; Elemental Analysis for C_18_H_15_BrN_2_O_3_: C, 55.83; H, 3.90; N, 7.23, Found C, 56.07; H, 3.88; N, 7.27.

##### 4.1.6.19. 1-((1E,3E)-4-(Benzo[d]1,3dioxol-5-yl)buta-1,3-dien-1-yl)-3–(3-bromophenyl)urea (8s)

White crystals (yield 48%), m.p: 253–256 °C; ^1^H NMR (DMSO-d_6_, 400 MHz) *δ ppm*: 8.88 (s, 1H, NH–CO), 8.79 (d, *J* = 10.7 Hz, 1H), NH–CO, 7.47–7.42 (m, 4H, aromatic), 7.09 (s, 1H, aromatic), 6.99 (dd, *J* = 13.9, 10.7 Hz, 1H, olefinic), 6.93–6.75 (m, 3H, aromatic and aliphatic), 6.29 (d, *J* = 15.6 Hz, 1H, olefinic), 6.00 (s, 2H, C**H_2_**O_2_), 5.87 (dd, *J* = 13.9, 10.8 Hz, 1H, olefinic); ^13 ^C NMR (DMSO-d_6_, 100 MHz) *δ ppm*: 151.9, 148.2, 146.4, 139.4, 133, 132, 128.3, 127.2, 126.6, 120.7, 120.6, 113.9, 110.4, 108.8, 105.1, 101.3; Elemental Analysis for C_18_H_15_BrN_2_O_3_: C, 55.83; H, 3.90; N, 7.23, Found C, 55.98; H, 3.87; N, 7.30.

##### 4.1.6.20. 1-((1E,3E)-4-(Benzo[d]1,3dioxol-5-yl)buta-1,3-dien-1-yl)-3–(2,5-dichlorophenyl)urea (8t)

White crystals (yield 47%), m.p: 228–231 °C; ^1^H NMR (DMSO-d_6_, 400 MHz) *δ ppm*: 9.57 (d, *J* = 10.5 Hz, 1H, NH–CO), 8.46 (s, 1H, NH–CO), 8.31 (d, *J* = 2.5 Hz, 1H, aromatic), 7.50 (d, *J* = 8.6 Hz, 1H, olefinic), 7.11 (s, 2H, aromatic), 7.09 (*brs*, 1H), 6.98 (dd, *J* = 13.9, 10.5 Hz, 1H, olefinic), 6.91–6.78 (m, 3H, aromatic), 6.34 (d, *J* = 15.6 Hz, 1H, olefinic), 6.01 (s, 2H, C**H_2_**O_2_), 5.88 (dd, *J* = 14.0, 10.7 Hz, 1H, olefinic); ^13^C NMR (DMSO-d_6_, 100 MHz) *δ ppm*: 151.6, 148.2, 146.5, 137.5, 132.9, 132.4, 131, 127.6, 127.3, 126.9, 123.3, 120.7, 120.5, 120.4, 111.2, 108.2, 105.2, 101.4; Elemental Analysis for C_18_H_14_Cl_2_N_2_O_3_: C, 57.31; H, 3.74; N, 7.43, Found C, 57.16; H, 3.77; N, 7.52.

##### 4.1.6.21. 1-((1E,3E)-4-(Benzo[d]1,3dioxol-5-yl)buta-1,3-dien-1-yl)-3–(3,4-dichlorophenyl)urea (8u)

White crystals (yield 48%), m.p: 235–237 °C; ^1^H NMR (DMSO-d_6_, 400 MHz) *δ ppm*: 9.06 (s, 1H, NH–CO), 8.90 (d, *J* = 10.6 Hz, 1H, NH–CO), 7.86 (s, 1H, aromatic), 7.52 (d, *J* = 8.8 Hz, 1H, aromatic), 7.35 (d, *J* = 8.9 Hz, 1H, aromatic), 7.10 (s, 1H, aromatic), 6.98 (t, *J* = 12.1 Hz, 1H, olefinic), 6.88–6.75 (m, 3H, aromatic and olefinic), 6.30 (d, *J* = 15.6 Hz, 1H, olefinic), 6.00 (s, 2H, C**H_2_**O_2_), 5.90 (t, *J* = 12.4 Hz, 1H, olefinic); ^13 ^C NMR (DMSO-d_6_, 100 MHz) *δ ppm*: 151.9, 148.2, 146.5, 140.2, 132.9, 131.5, 131.1, 128, 127.1, 126.9, 123.7, 120.7, 119.8, 118.9, 110.8, 108.8, 105.1, 101.3; Elemental Analysis for C_18_H_14_Cl_2_N_2_O_3_: C, 57.31; H, 3.74; N, 7.43, Found C, 57.53; H, 3.71; N, 7.50.

##### 4.1.6.22. 1-((1E,3E)-4-(Benzo[d]1,3dioxol-5-yl)buta-1,3-dien-1-yl)-3–(3-chloro-4-fluorophenyl)urea (8v)

White crystals (yield 50%), m.p: 240–242 °C; ^1^H NMR (DMSO-d_6_, 400 MHz) *δ ppm*: 9.24 (d, *J* = 10.7 Hz, 1H, NH–CO), 8.77 (s, 1H, NH–CO), 8.11 (t, *J* = 8.8 Hz, 1H, aromatic), 7.57 (d, *J* = 10.7 Hz, 1H, aromatic), 7.36 (d, *J* = 8.9 Hz, 1H), 7.09 (s, 1H), 6.98 (dd, *J* = 13.9, 10.6 Hz, 1H, olefinic), 6.89–6.77 (m, 3H, aromatic and olefinic), 6.31 (d, *J* = 15.6 Hz, 1H, olefinic), 6.00 (brs, 2H, C**H_2_**O_2_), 5.85 (dd, *J* = 13.9, 10.8 Hz, 1H, olefinic);^13^C NMR (DMSO-d_6_, 100 MHz) *δ ppm*: 153.5, 151.7, 151.0, 148.2, 146.4, 132.9, 128, 127.9, 127.7, 127.5, 127.5, 127, 126.9, 122.3, 122.2, 120.6, 118.9, 118.6, 113.3, 113.2, 110.7, 108.8, 105.1, 101.3; Elemental Analysis for C_18_H_14_Cl_2_N_2_O_3_: C, 59.93; H, 3.91; N, 7.77, Found C, 60.23; H, 3.88; N, 7.85.

##### 4.1.6.23. 1-((1E,3E)-4-(Benzo[d]1,3dioxol-5-yl)buta-1,3-dien-1-yl)-3–(4-bromo-2-fluorophenyl)urea (8w)

White crystals (yield 45%), m.p: 245–247 °C; ^1^H NMR (DMSO-d_6_, 400 MHz) *δ ppm*: 8.88 (s, 1H, NH–CO), 8.79 (d, *J* = 10.7 Hz, 1H, NH–CO), 7.50–7.36 (m, 4H, aromatic), 7.13–7.06 (m, 1H, aromatic), 6.99 (dd, *J* = 13.9, 10.6 Hz, 1H, olefinic), 6.86–6.78 (m, 2H aromatic and olefinic), 6.29 (d, *J* = 15.6 Hz, 1H, olefinic), 6.00 (s, 2H, C**H_2_**O_2_), 5.87 (dd, *J* = 13.9, 10.8 Hz, 1H, olefinic); ^13 ^C NMR (DMSO-d_6_, 100 MHz) *δ ppm*: 155.5, 144.4, 132.9, 130.1, 125.4, 122.2, 120.7, 118.8, 113.3, 110.7, 108.8, 105.2, 101.3; Elemental Analysis for C_18_H_14_BrFN_2_O_3_: C, 53.35; H, 3.48; N, 6.91, Found C, 53.47; H, 3.46; N, 6.89.

##### 4.1.6.24. 1-((1E,3E)-4-(Benzo[d]1,3dioxol-5-yl)buta-1,3-dien-1-yl)-3–(4-chloro-3-(trifluoromethyl)phenyl)urea (8x)

White crystals (yield 44%), m.p: 233–235 °C; ^1^H NMR (DMSO-d_6_, 400 MHz) *δ ppm*: 9.45 (s, 1H, NH–CO), 9.21 (d, *J* = 10.4 Hz, 1H, NH–CO), 7.78 (d, *J* = 6.8 Hz, 1H, aromatic), 7.33 (d, *J* = 6.8 Hz, 2H, aromatic), 7.09 (s, 1H, aromatic), 6.99 (dd, *J* = 13.9, 10.6 Hz, 1H, olefinic), 6.88–6.75 (m, 3H, Aromatic and olefinic), 6.27 (d, *J* = 15.6 Hz, 1H, olefinic), 6.00 (s, 2H, C**H_2_**O_2_), 5.86 (dd, *J* = 13.9, 10.8 Hz, 1H, olefinic); ^13 ^C NMR (DMSO-d_6_, 100 MHz) *δ ppm*: 154, 152.2, 151.6, 148.2, 146.4, 137.4, 137.4, 133, 128.2, 127.2, 126.6, 120.6, 119.7, 119.7, 119.5, 118.8, 118.7, 117.5, 117.2, 110.4, 108.8, 105.1, 101.3; Elemental Analysis for C_19_H_14_ClF_3_N_2_O_3_: C, 55.56; H, 3.44; N, 6.82, Found C, 55.73; H, 3.45; N, 6.75.

##### 4.1.6.25. 1-((1E,3E)-4-(Benzo[d]1,3dioxol-5-yl)buta-1,3-dien-1-yl)-3–(3-methoxyphenyl)urea (8y)

White crystals (yield 53%), m.p: 202–203 °C; ^1^H NMR (DMSO-d_6_, 400 MHz) *δ ppm*: 8.72 (d, *J* = 10.5 Hz, 2H, NH–CO), 7.23–7.12 (m, 2H, aromatic), 7.09 (s, 1H, aromatic), 7.05–6.90 (m, 2H, aromatic), 6.89–6.74 (m, 3H, aromatic and olefinic), 6.56 (d, *J* = 8.3 Hz, 1H, olefinic), 6.28 (d, *J* = 15.5 Hz, 1H, olefinic), 6.00 (d, *J* = 2.3 Hz, 2H, C**H_2_**O_2_), 5.85 (t, *J* = 12.4 Hz, 1H, olefinic), 3.73 (t, *J* = 1.9 Hz, 3H, methoxy); ^13 ^C NMR (DMSO-d_6_, 100 MHz) *δ ppm*: 160.1, 152, 151.9, 148.2, 141.1, 133.1, 130.1, 128.4, 128.3, 128.2, 126.5, 120.6, 110.9, 110, 108.8, 107.8, 105.1, 101.3, 55.4; Elemental Analysis for C_19_H_18_N_2_O_4_: C, 67.45; H, 5.36; N, 8.28, Found C, 67.63; H, 5.32; N, 8.33.

### 4.2. Biological evaluations

All the utilised procedures for the biological evaluations have been performed as described previously; cytotoxicity^15^^,^^30^^,^^31^, cell cycle^32^^,^^33^, apoptosis Annexin V-FITC/PI^34^ and VEGFR-2 inhibitory^35^ assays, and was described in details in the Supplementary Materials.

### 4.3. *In silico* study

To investigate the potential mechanism of action of the prepared derivative, Target fishing was employed using the chemical structure of **8q** as a template. Swiss TargetPrediction, was used using the default settings^36^ to give set of targets where the top 5 of them was screened for their correlation with triple negative breast cancer. The chemical structure of prepared derivatives were drawn using Chemoffice software^37^ and saved as SDF file, which was exported MOE software^38^ for 3 D structure generation and energy minimisation using MMFF94x forcefield and the file was saved as mol2 file. The 3 D structure of Vascular endothelia growth factor 2 (VEGFR-2) was downloaded from Protein data bank PDB: 4ASD where water molecules were removed, bond orders was assigned, hydrogens were added, hydrogen bonds were optimised, charges were corrected, the protein complex was minimised and saved as PDB.

The optimised PDB was loaded in protein preparation module integrated in Leadit software^39^^,^^40^ where the active site was defined as sphere with radius 6.5 A around the co-crystallized ligand. The software was validated by redocking the co-crystallized ligand and calculating RMSD between the produced and experimental pose. The Compounds were loaded to Leadit interface and were docked to the active site. HYDE assessment was employed to re-rank the compounds by taking desolvation energy in consideration^41^and to predict their dissociation constant K_i_. Finally, best poses were visualised to investigate their interaction with the active site using Discovery studio ligand interaction visualiser^42^.

## Supplementary Material

Supplemental Material
