# Supplementary material for: Natural inspired piperine-based ureas and amides as novel antitumor agents towards breast cancer
Source: J Enzyme Inhib Med Chem. 2021 Dec 11;37(1):39–50. doi: 10.1080/14756366.2021.1988944 (PMC8667897; doi:10.1080/14756366.2021.1988944)

Supporting Information

Natural inspired piperine-based ureas and amides as novel antitumor agents toward breast cancer

Diaaeldin M. Elimam, Abdullah A. Elgazar, Fardous F El-Senduny, Ramadan A. El-Domany, Farid A Badria*, Wagdy M. Eldehna*

Tables of Contents

1. **Cytotoxicity assay** 2
2. **Cell Cycle Analysis** 2
3. **AnnexinV-FITC/PI Apoptosis Assay** 3
4. **Table S1.** Effect of 8q on cell cycle and apoptosis in MDA-MB-231 cells 3
5. **Table S2.** Top 5 Targets predicted by Swisstargetpredict server for **8q** 3
6. **VEGFR-2 Kinase Inhibitory Activity** 4
7. **Figure S1** 4
8. **Figure S2** 5
9. **NMR Spectra** 6-51

1. Cytotoxicity assay

The examined breast (MDA-MB-231), ovarian (A2780CP) and hepatocellular (HepG2) cancer cell lines have been obtained from American Type Culture Collection (ATCC). The cells were maintained in Dulbecco's modified Eagle's medium (DMEM) supplemented with 10% heat inactivated fetal calf serum (GIBCO), penicillin (100 U/ml) and streptomycin (100 µg/ml) at 37 ^{o}C in humidified atmosphere containing 5% CO_2. Cells at a concentration of 0.50 x 10^6 were grown in a 25 cm^2 flask in 5 ml of culture medium. The anti-proliferative activity of the tested piperine-based amides (**5a-i**) and ureas (**8a-y**) measured *in vitro* using the MTT assay. Briefly, cells were plated out in 96-well microtiter plate (0.5X10^4 cells/well) and incubated for 24 h before treatment with the tested benzofurans to allow cells to attach to the bottom of the well of the plate. Tested benzofuranswere were dissolved in DMSO at 1 mg/ml immediately before use and diluted to the appropriate volume just before addition to the cell culture. Different concentrations of the tested piperine-based amides (**5a-i**) and ureas (**8a-y**) were added to the cells (three wells were prepared for each individual dose). Cells were incubated with the piperine derivatives for 48 h at 37°C and in atmosphere of 5% CO_2. At the end of exposure, MTT solution in PBS (5 mg/ml) was then added to all well including no cell blank and left to incubate for 90 min. The formation of formazan crystals were visually confirmed using phase contract microscopy. DMSO (100 µl/well) was added to dissolve the formazan crystals with shaking for 10 min after which the absorbance was read at 590 nm against no cell blanks on a microplate reader. The relation between percent of surviving fraction and log drug concentration is plotted to get the survival curve for each cell line. The concentration required for 50% inhibition of cell viability (IC_{50}) was calculated and the results are presented in Table 1.

2. Cell Cycle Analysis

Breast cancer MDA-MB-231 cells were treated with compound **8q** for 24 h (at its IC_{50} concentration), and then cells were washed twice with ice-cold phosphate buffered saline (PBS). Subsequently, the treated cells were collected by centrifugation, fixed in ice-cold 70% (*v*/*v*) ethanol, washed with PBS, re-suspended with 100 µg/mL RNase, stained with 40 µg/mL PI, and analyzed by flow cytometry using FACS Calibur (Becton Dickinson, BD, Franklin Lakes, NJ, USA). The cell cycle distributions were calculated using CellQuest software 5.1 (Becton Dickinson).

3. Annexin V-FITC Apoptosis Assay

Phosphatidylserine externalization was assayed using Annexin V-FITC/PI apoptosis detection kit (BD Biosciences, USA) according to the manufacturer's instructions. Breast cancer MDA-MB-231 cells were cultured to a monolayer then treated with compound **8q** at its IC_{50} concentration. Briefly, cells were then harvested *via* trypsinization, and rinsed twice in PBS followed by binding buffer. Moreover, cells were re-suspended in 100 µL of binding buffer with the addition of 1 µL of FITC-Annexin V followed by an incubation period of 30 min at 4 °C. Cells were then rinsed in binding buffer and resuspended in 150 µL of binding buffer with the addition of 1 µL of DAPI (1 µg/µL in PBS). Cells were then analyzed using the flow cytometer BD FACS Canto II and the results were interpreted with FlowJo7.6.4 software (Tree Star, Ashland, OR, USA).

4. Table S1. Effect of **8q** on cell cycle and apoptosis in MDA-MB-231 cells

	**%G0-G1**	**%S**	**%G2/M**	**%Sub-G1**
**8q**	36.52	21.57	23.17	18.74
**Control**	52.7	33.8	11.42	2.08
	**Total apoptosis**	**Early apoptosis**	**Late apoptosis**	**Necrosis**
**8q**	23.06	3.18	11.66	8.22
**Control**	2.12	0.39	0.11	1.62

5. Table S2. Top 5 Targets predicted by Swisstargetpredict server for **8q**

**Target**	**Common name**	**Uniport ID**	**ChEMBL ID**	**Target Class**
**Diacylglycerol O-acyltransferase 1**	DGAT1	O75907	CHEMBL6009	Enzyme
**Vascular endothelial growth factor receptor 2**	KDR	P35968	CHEMBL279	Kinase
**Rho-associated protein kinase 2**	ROCK2	O75116	CHEMBL2973	Kinase
**Tyrosine-protein kinase SYK**	SYK	P43405	CHEMBL2599	Kinase
**Estrogen receptor alpha**	ESR1	P03372	CHEMBL206	Nuclear receptor

6. VEGFR-2 Kinase Inhibitory Activity

The VEGFR-2 kinase inhibitory activity was determined *in vitro* for urea derivative **8q** using VEGFR2(KDR) Kinase Assay Kit (Catalog No. 40325) (BPS Bioscience, San Diego, CA, USA), according to the manufacturer's instructions.

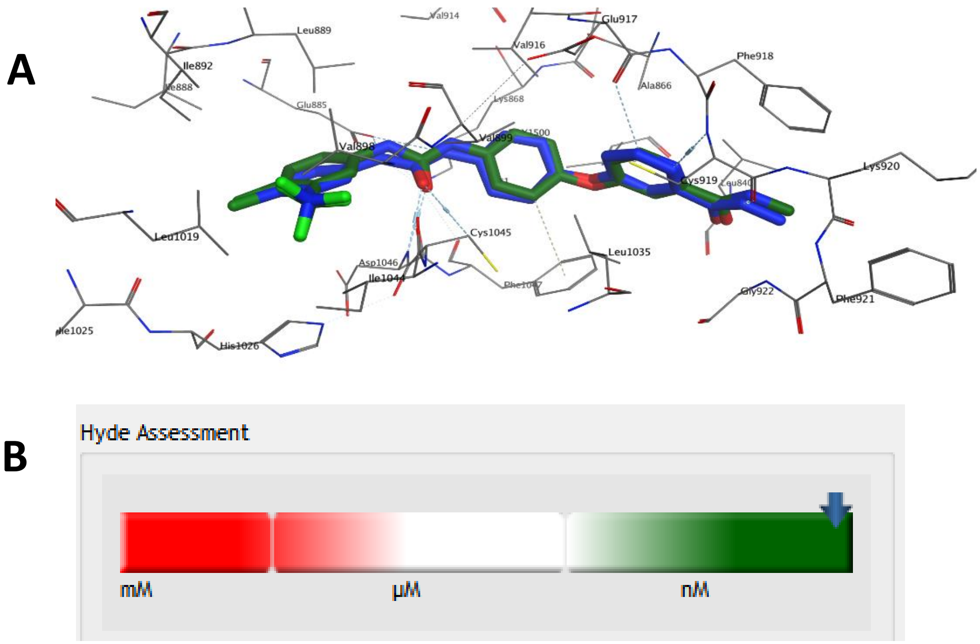

7. Figure S1. (**A**) Redocked pose of the co-crystallized ligand (green) aligned to the experimental pose (blue) with RMSD=0.8 in the active site of VEGFR-2 PDB: 4ASD; (**B**) Hyde assessment showing the predicted K_I at low nanomolar range.

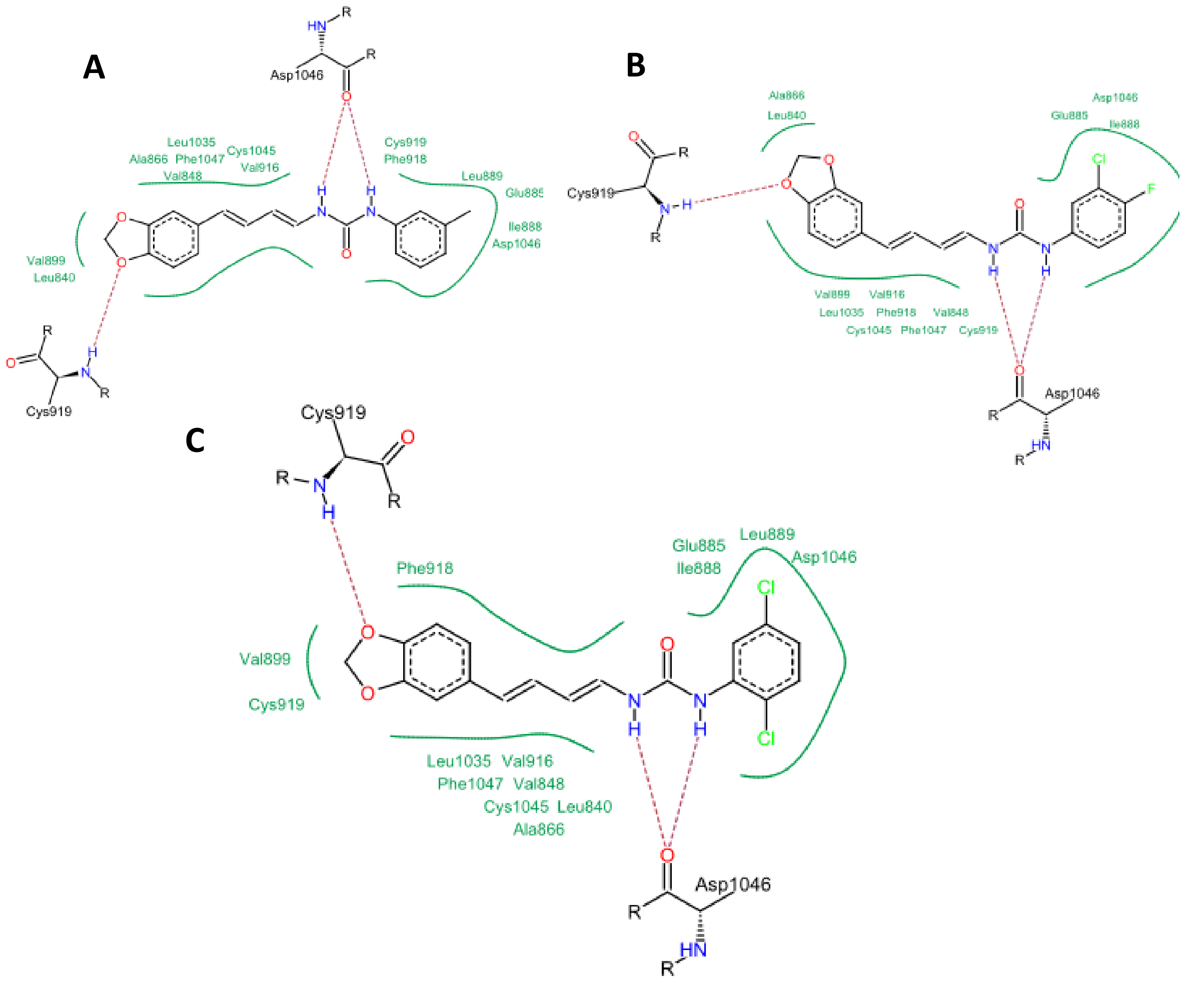

8. Figure S2. 2D interactions of 8b (A), 8w (B), 8t (C) in the active site of VEGFR-2. where hydrogen bonds are showed as dashed lines and hydrophobic contacts are illustrated as spline segments

Diaa Elimam-Pip-An-HNMR-DMSO-AF.10.fid — Diaa Elimam-Pip-An-HNMR-DMSO-AF

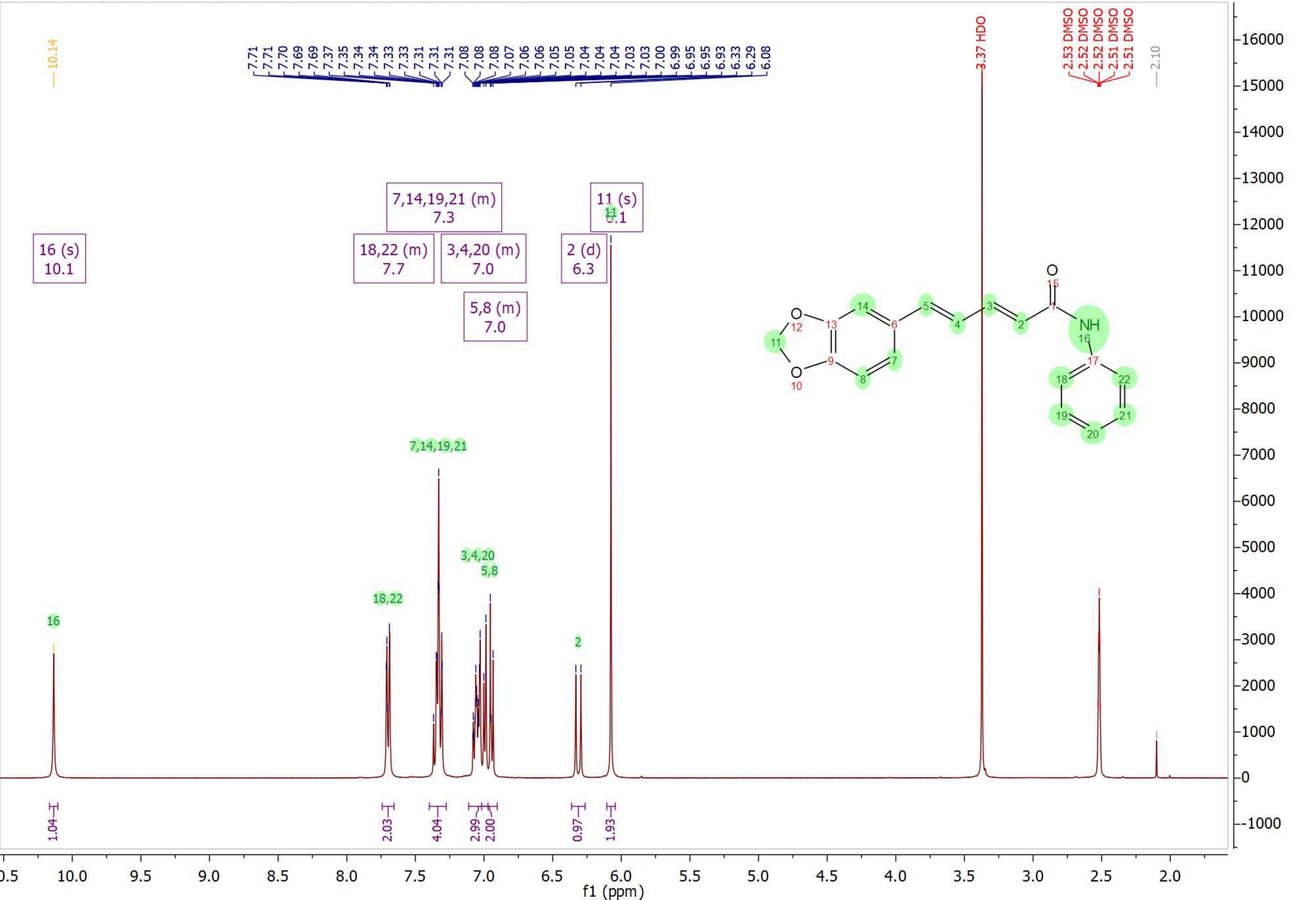

Diaa Elimam-Pip-An-Apt.10.fid — Diaa Elimam-Pip-An-Apt

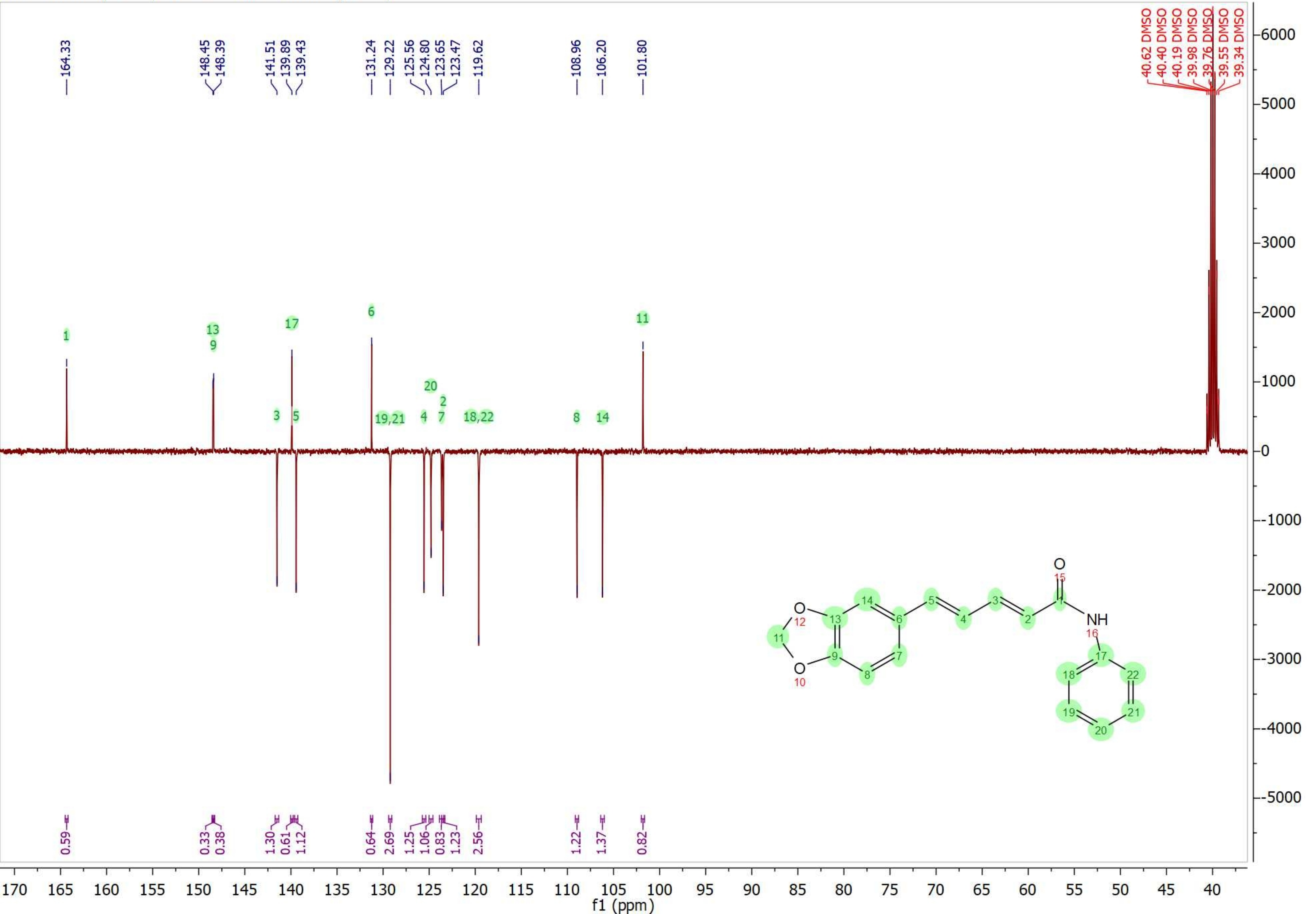

Diaa Elemam-3BrPAM-HNMR-DMSO-AF.10.fid — Diaa Elemam-3BrPAM-HNMR-DMSO-AF

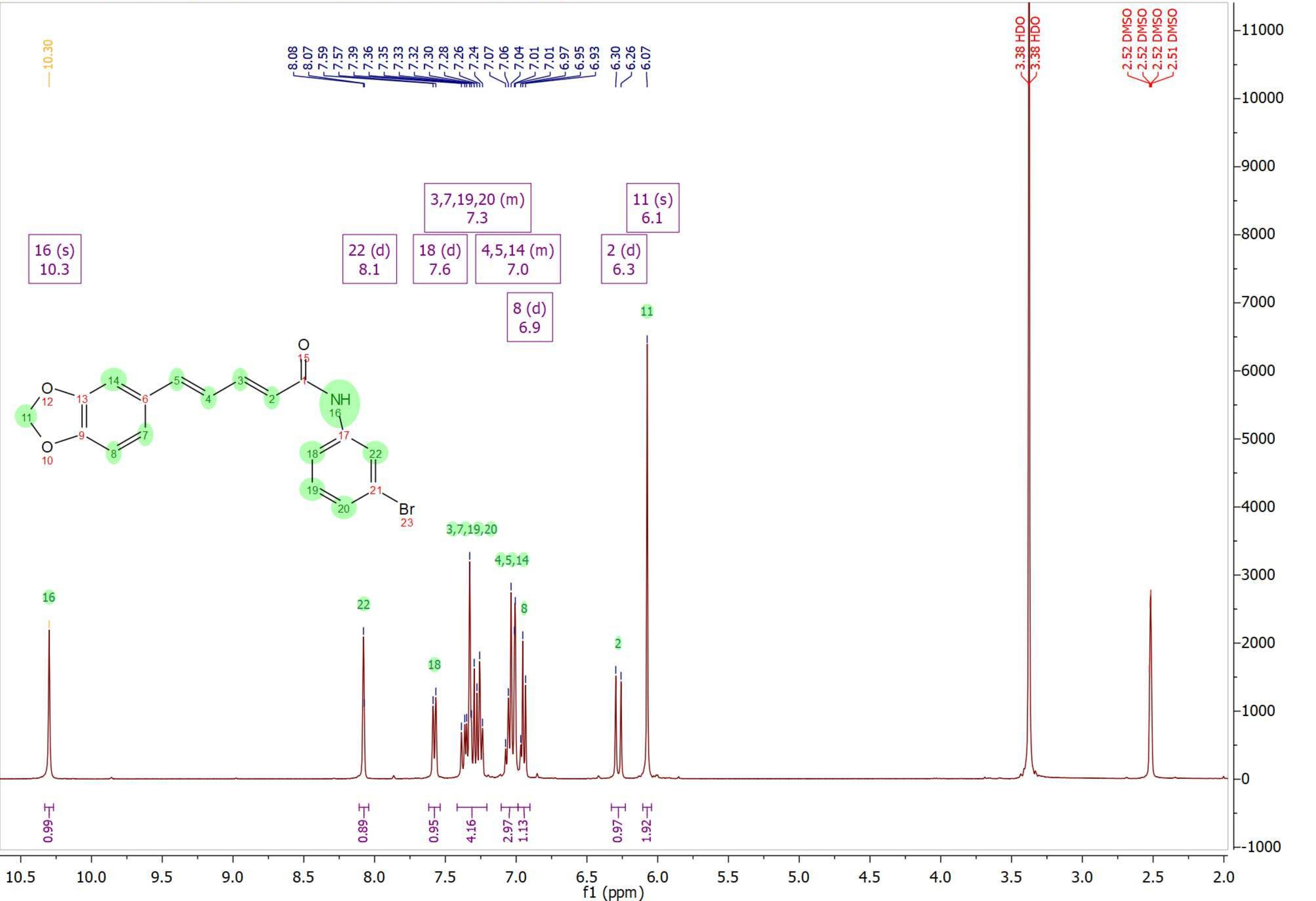

Diaa Elemam-3BrPAM-APT-AF.10.fid — Diaa Elemam-3BrPAM-APT-AF

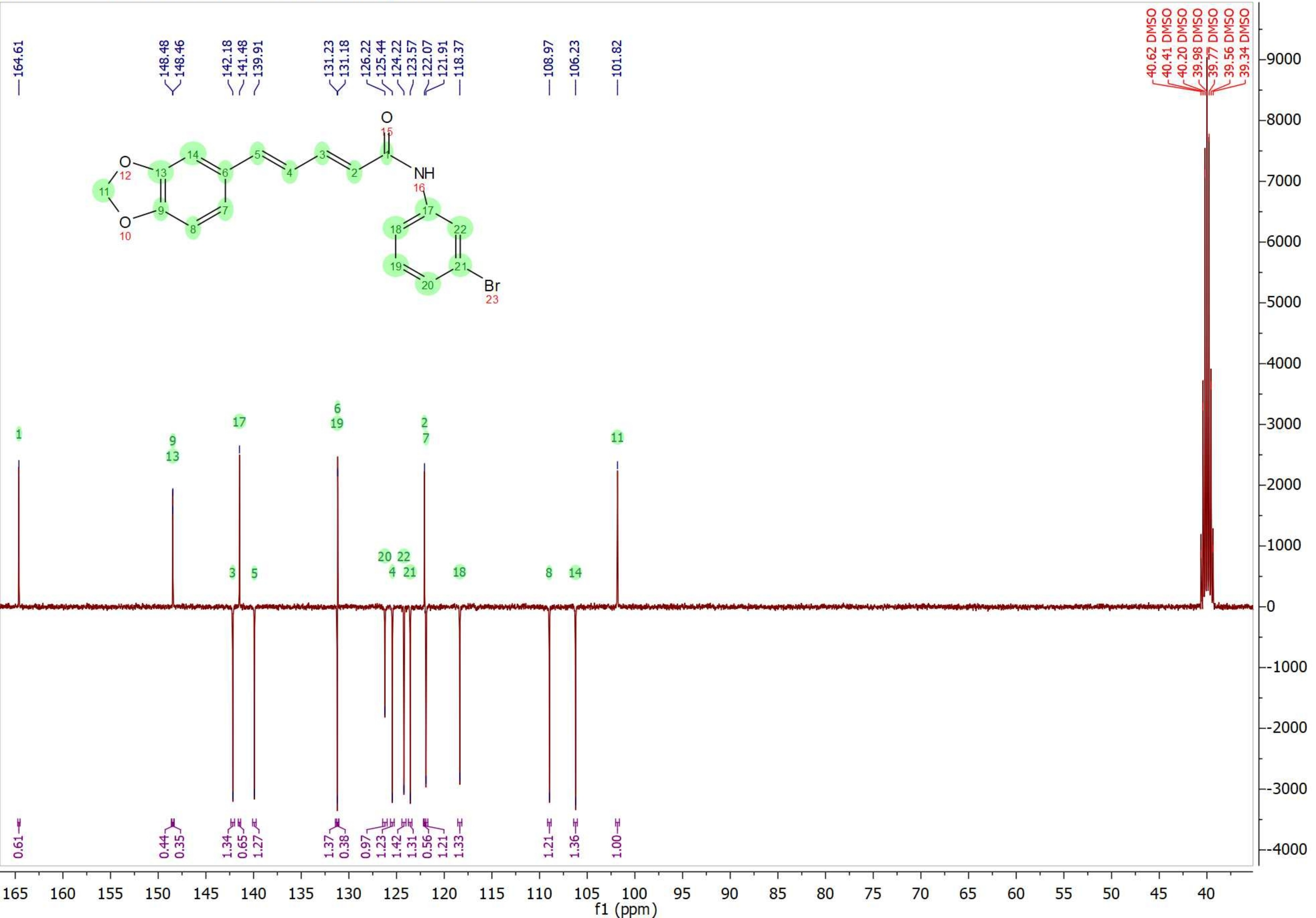

Diaa Elemam-3clPAM-HNMR-DMSO-AF.10.fid — Diaa Elemam-3clPAM-HNMR-DMSO-AF

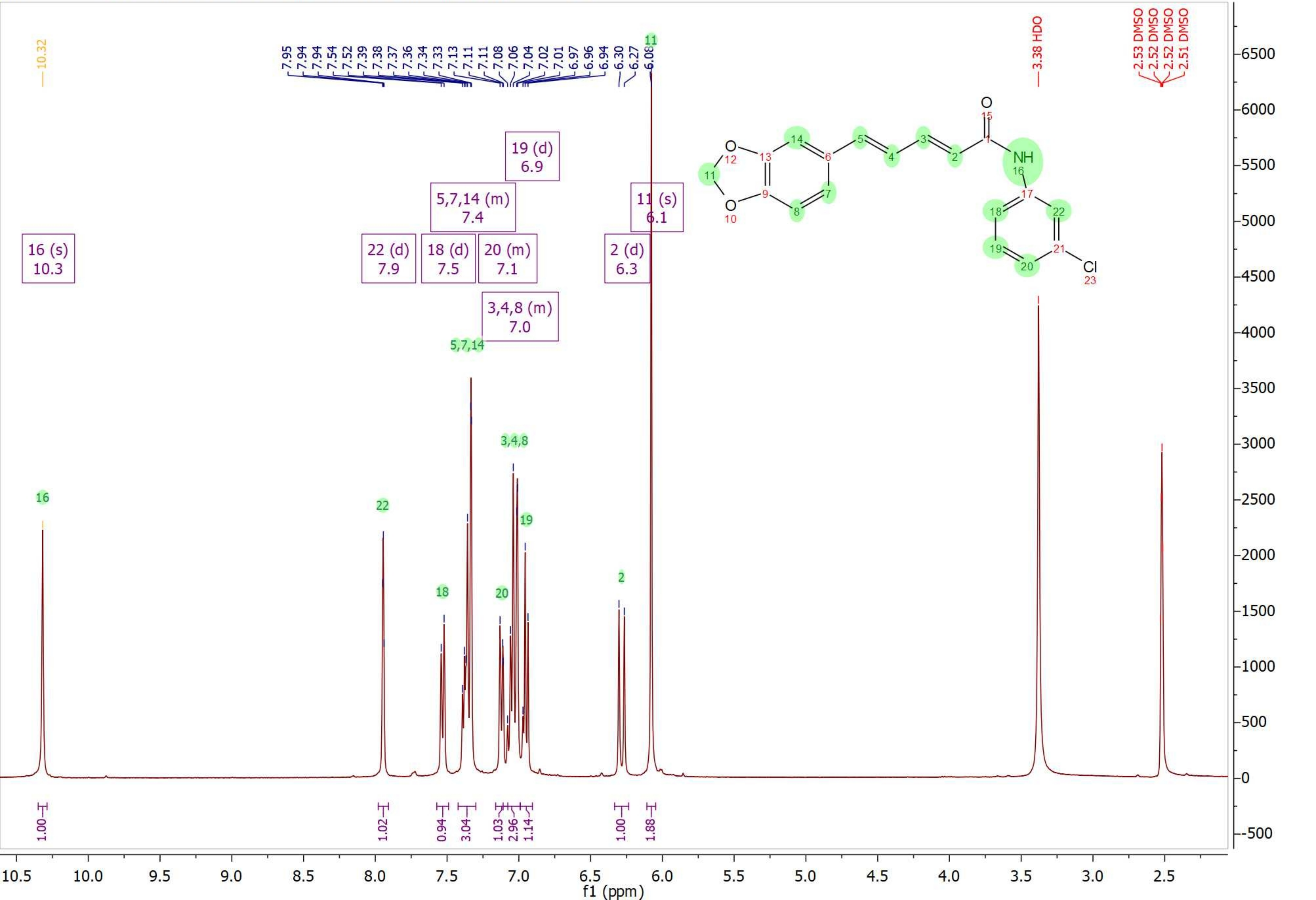

Diaa Elemam-3 cl PAM-APT-AF.10.fid — Diaa Elemam-3 cl PAM-APT-AF

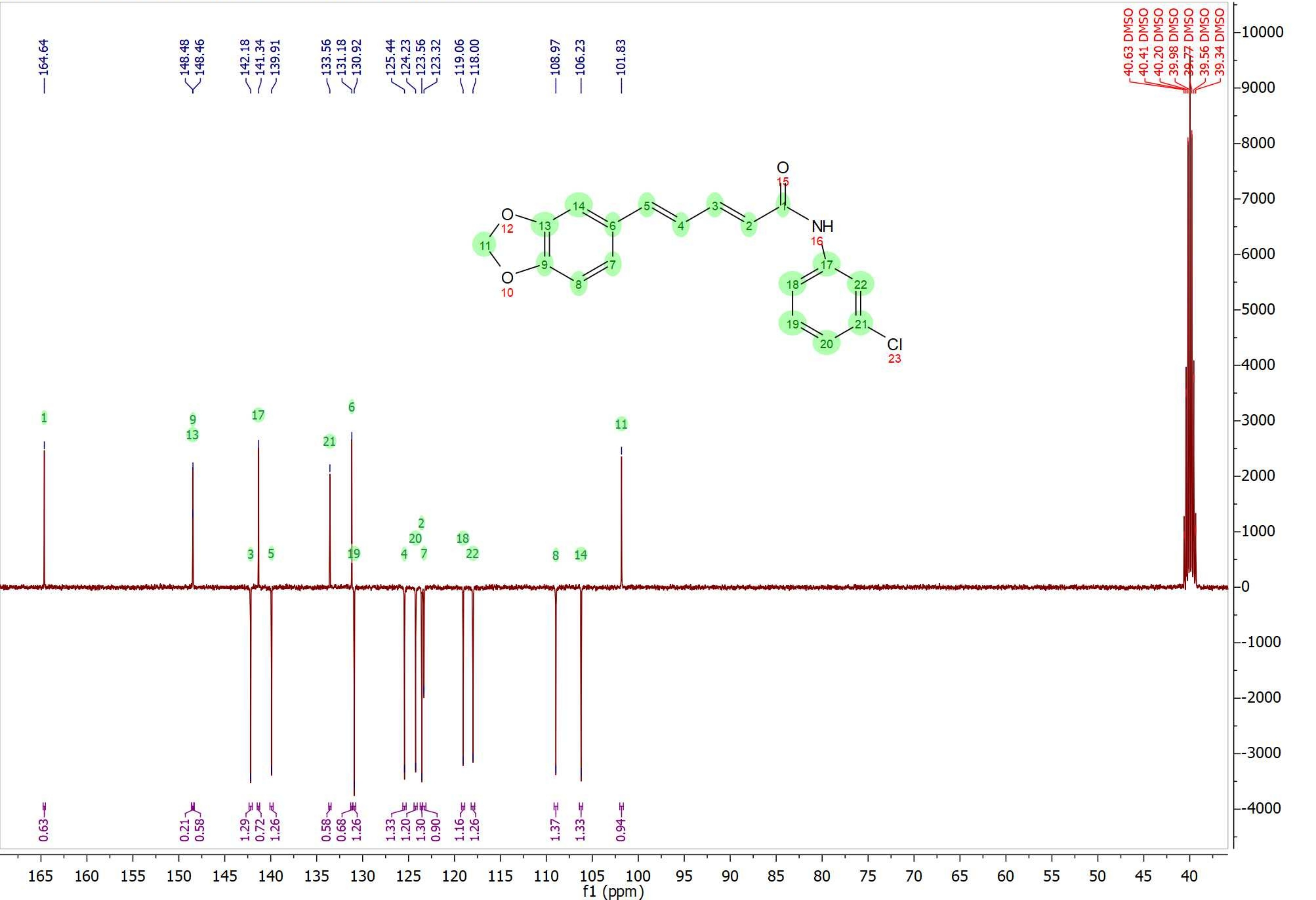

Diaa Elemam-3FPAM-HNMR-DMSO-AF.10.fid — Diaa Elemam-3FPAM-HNMR-DMSO-AF

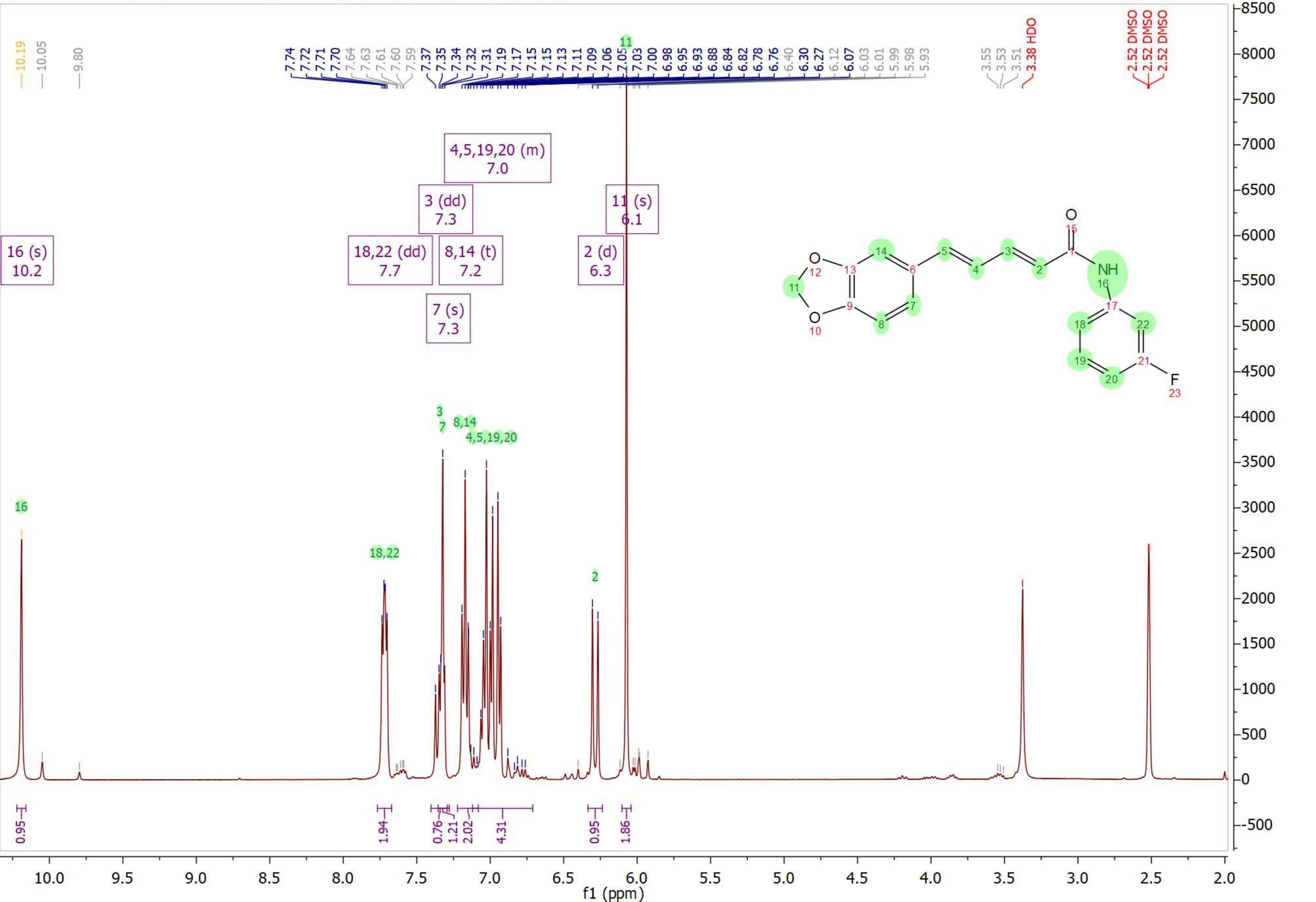

Diaa Elemam-3FPAM-APT-AF.10.fid — Diaa Elemam-3FPAM-APT-AF

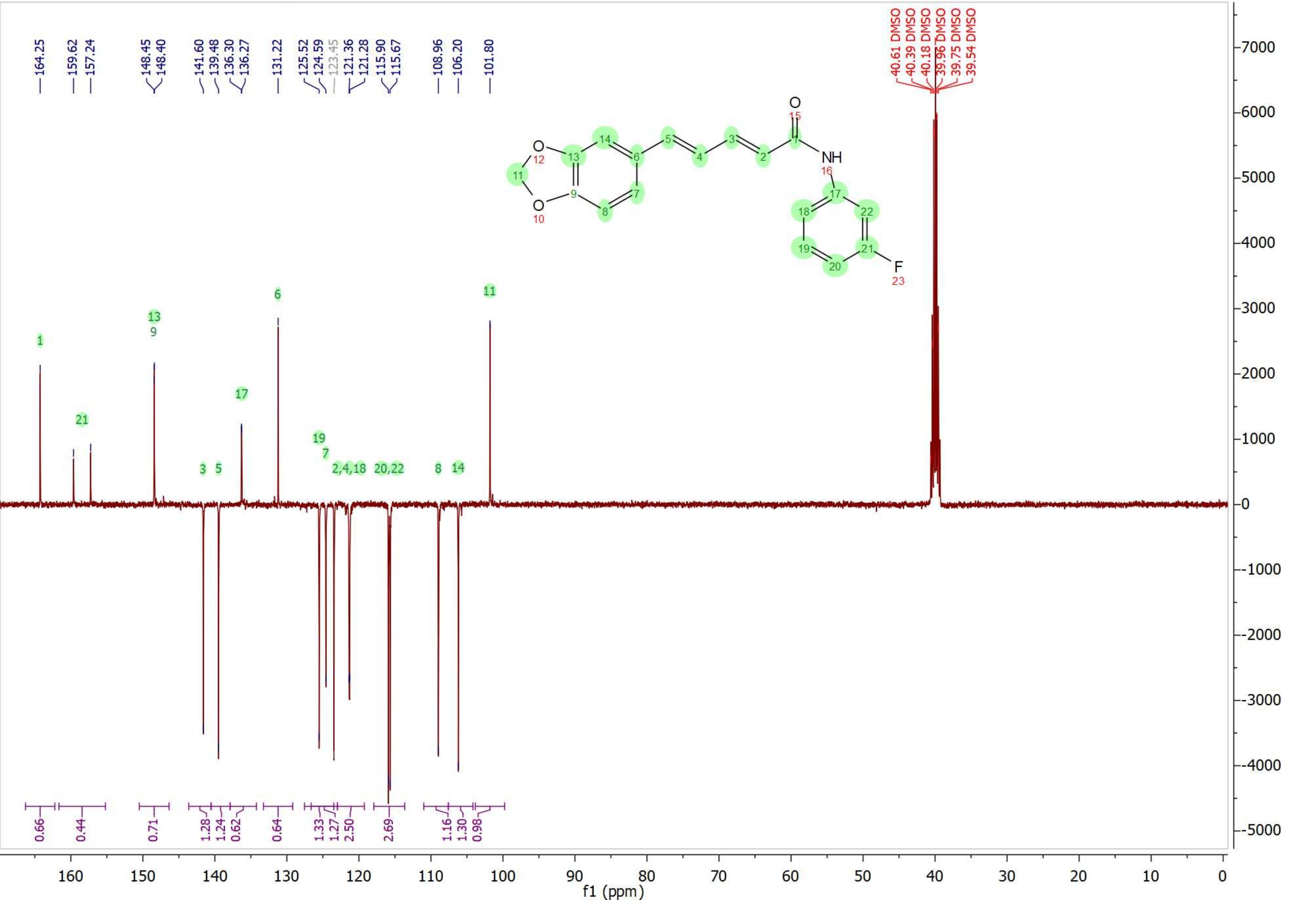

Diaa Eldin-2-F-P-amide-proton-ES.10.fid — Diaa Eldin-2-F-P-amide-proton

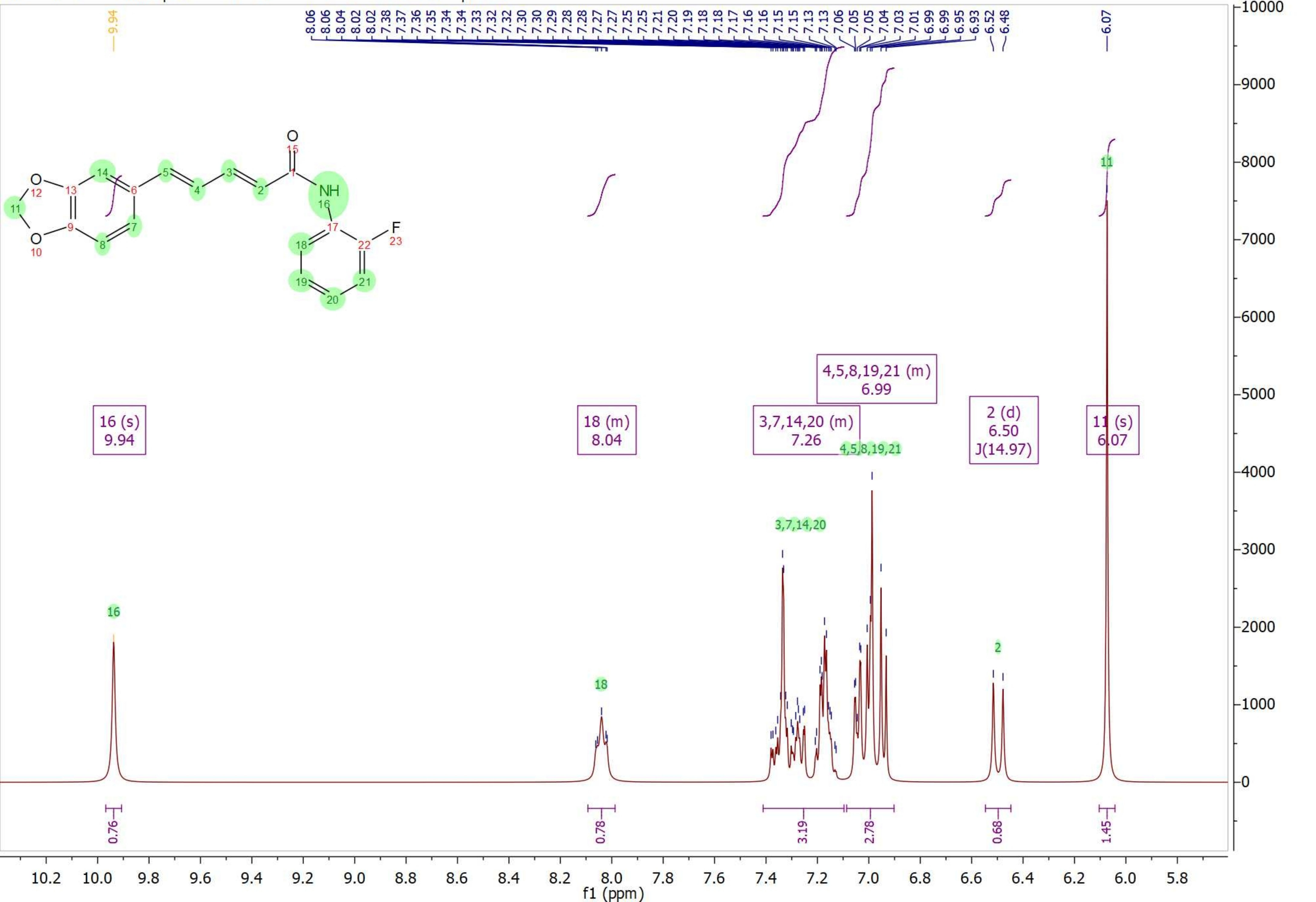

Diaa Elemam-3CF3-HNMR-DMSO-AF.10.fid — Diaa Elemam-3CF3-HNMR-DMSO-AF

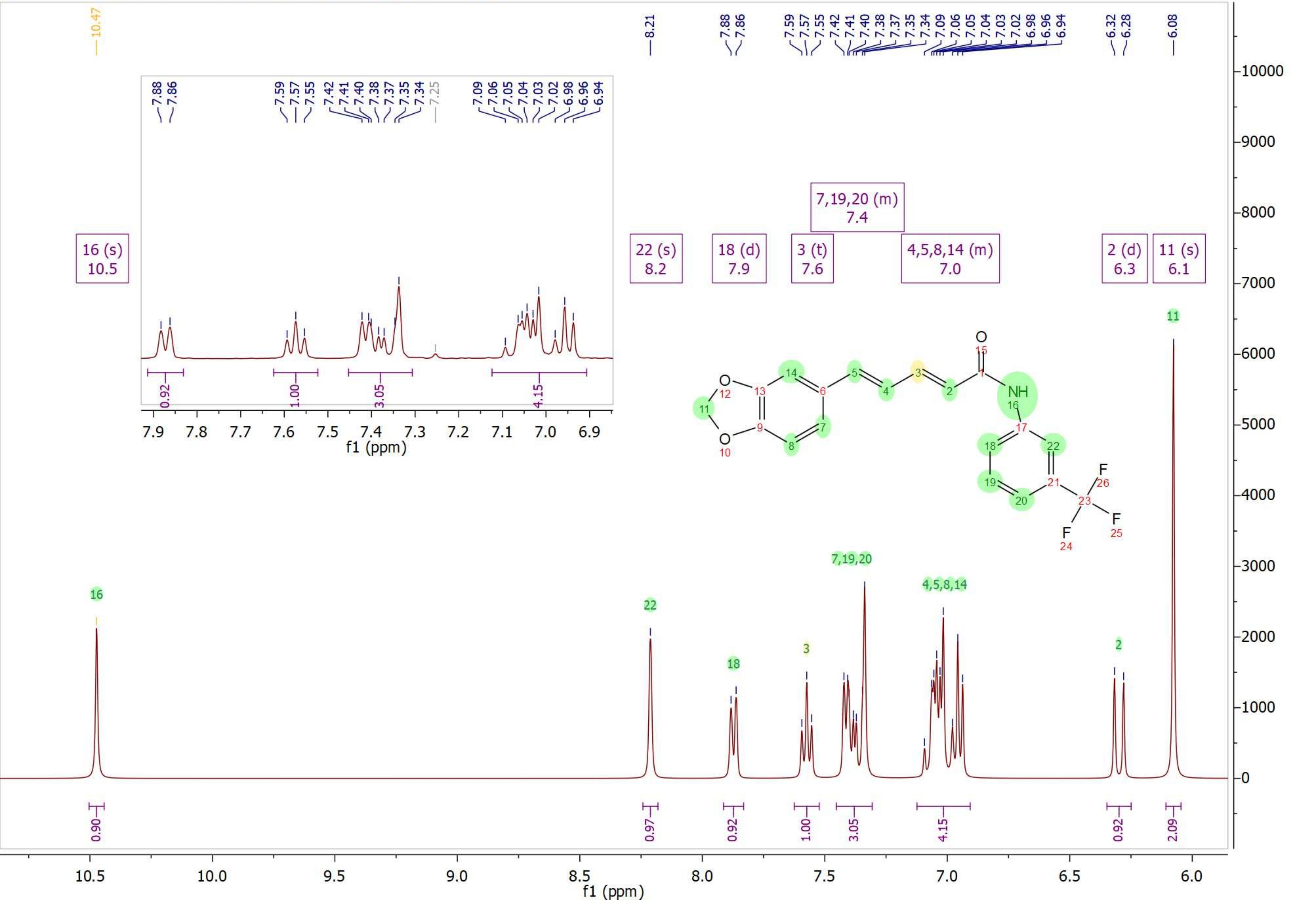

Diaa Elemam-3 CF3PAM-APT-AF.10.fid — Diaa Elemam-3 CF3PAM-APT-AF

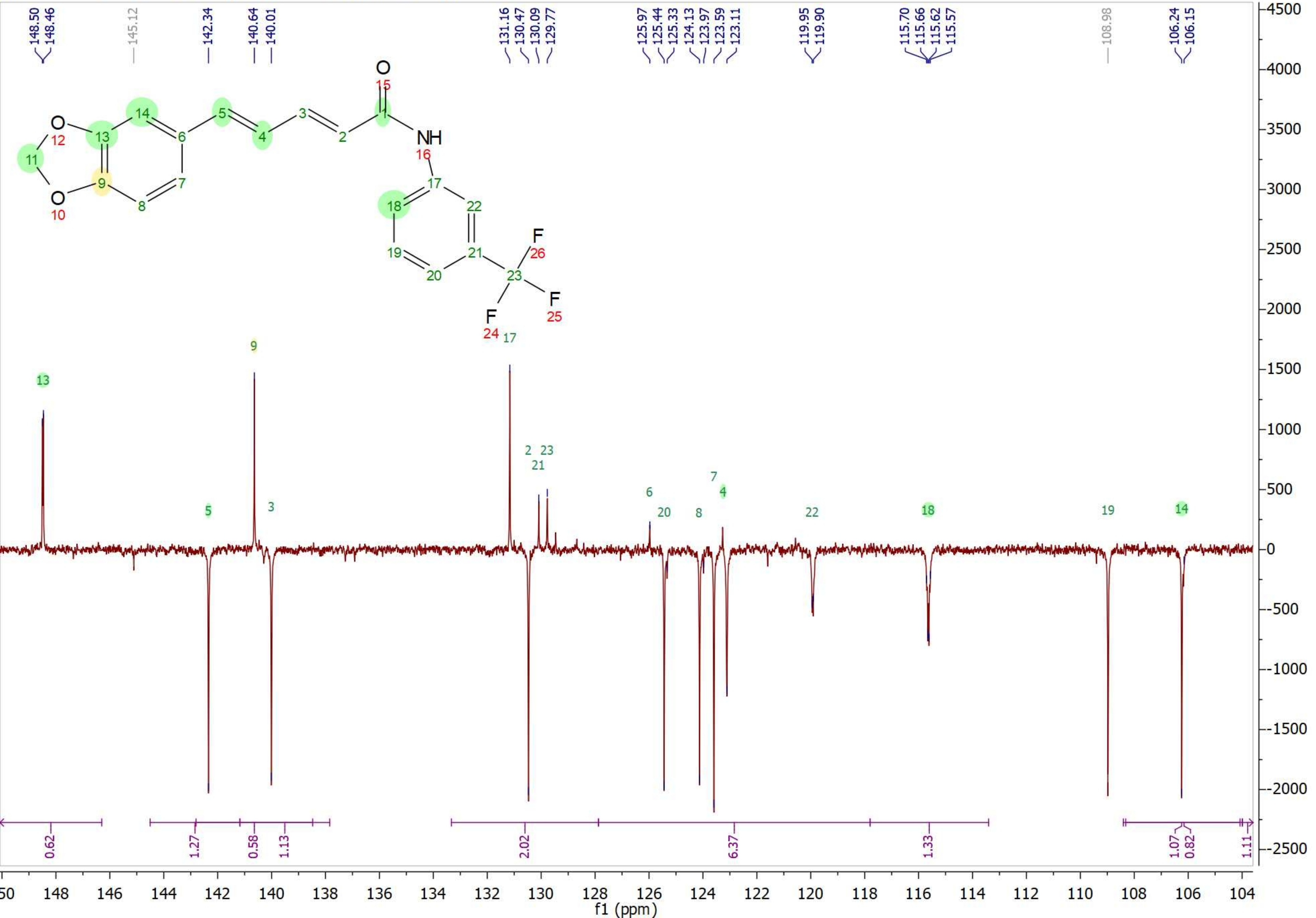

Diaa Elemam-3,4dcPAM-HNMR-DMSO-AF.10.fid — Diaa Elemam-3,4dcPAM-HNMR-DMSO-AF

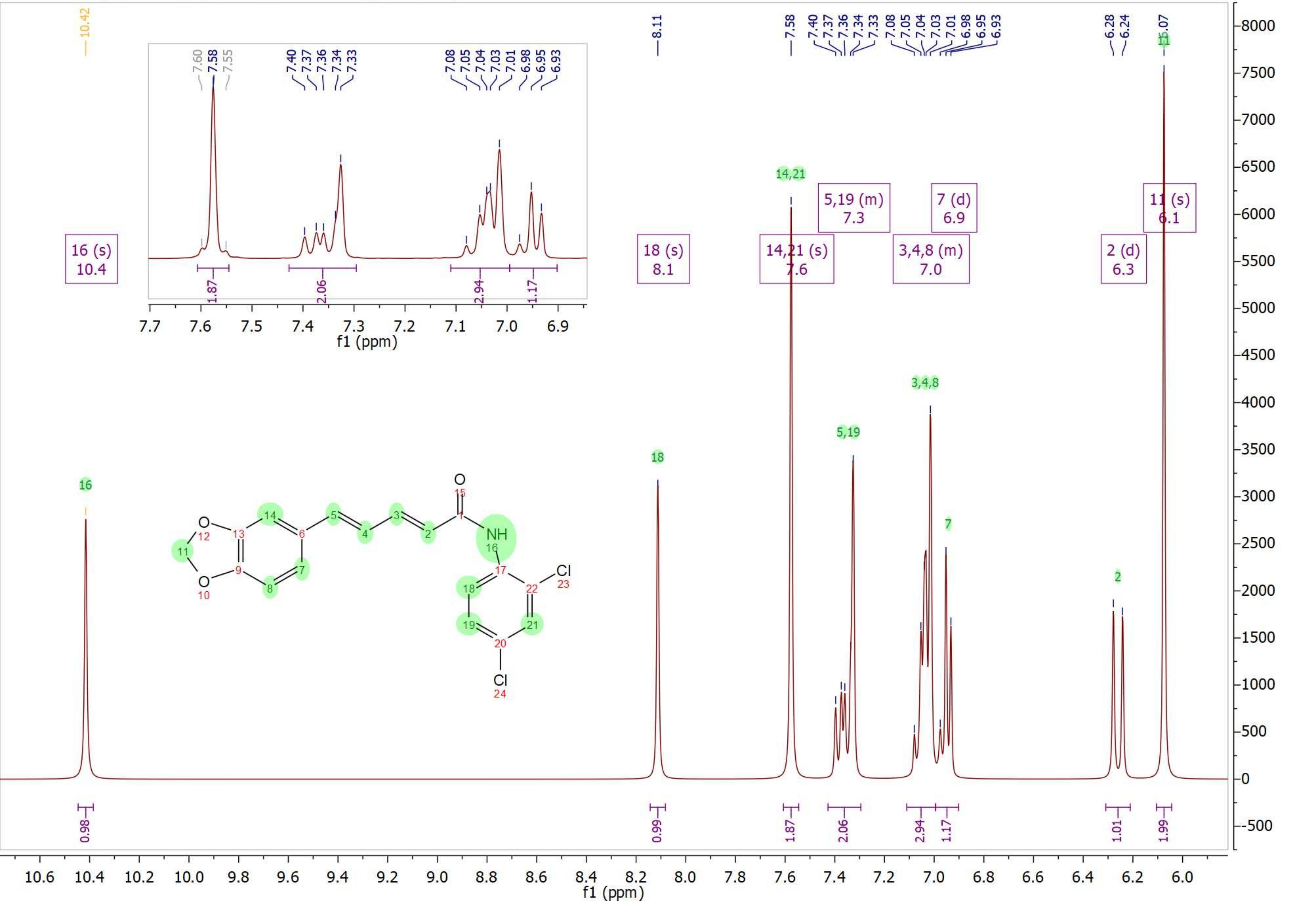

Diaa Elemam-3,4 dcPAM-APT-AF.10.fid — Diaa Elemam-3,4 dcPAM-APT-AF

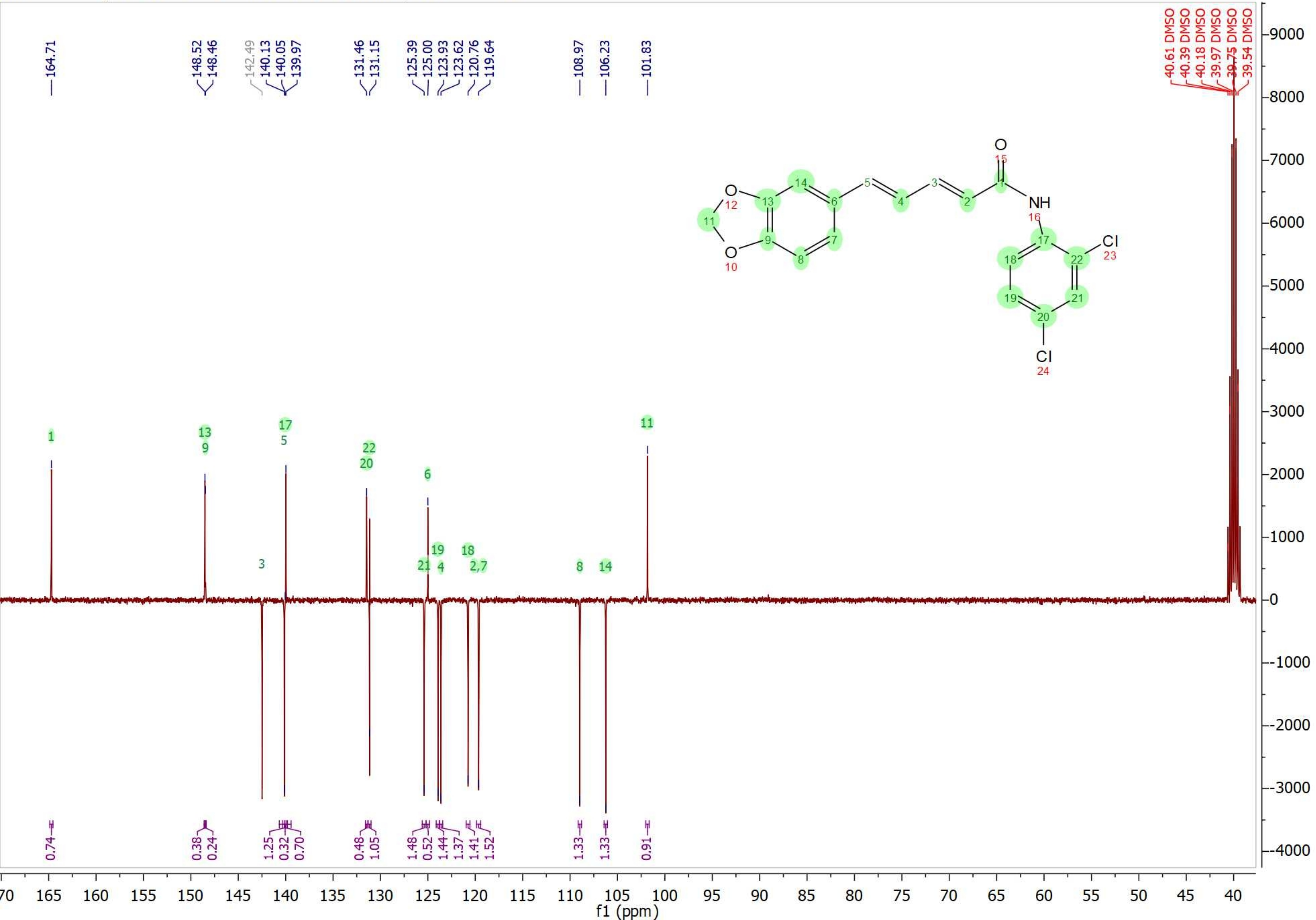

Diaa Elemam-3EtPAM-HNMR-DMSO-AF.10.fid — Diaa Elemam-3EtPAM-HNMR-DMSO-AF

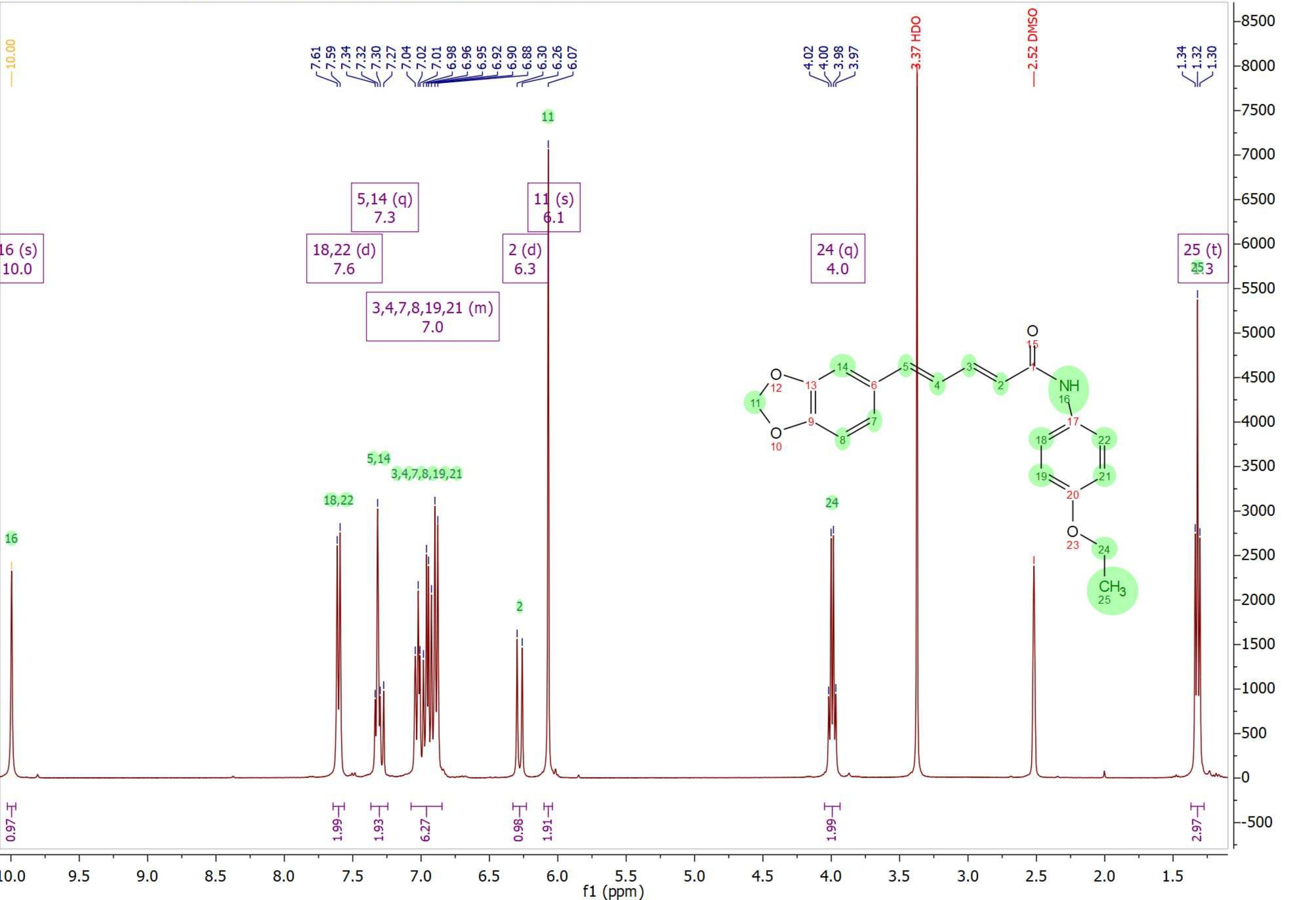

Diaa Elemam-4EtPAM-APT-AF.10.fid — Diaa Elemam-4EtPAM-APT-AF

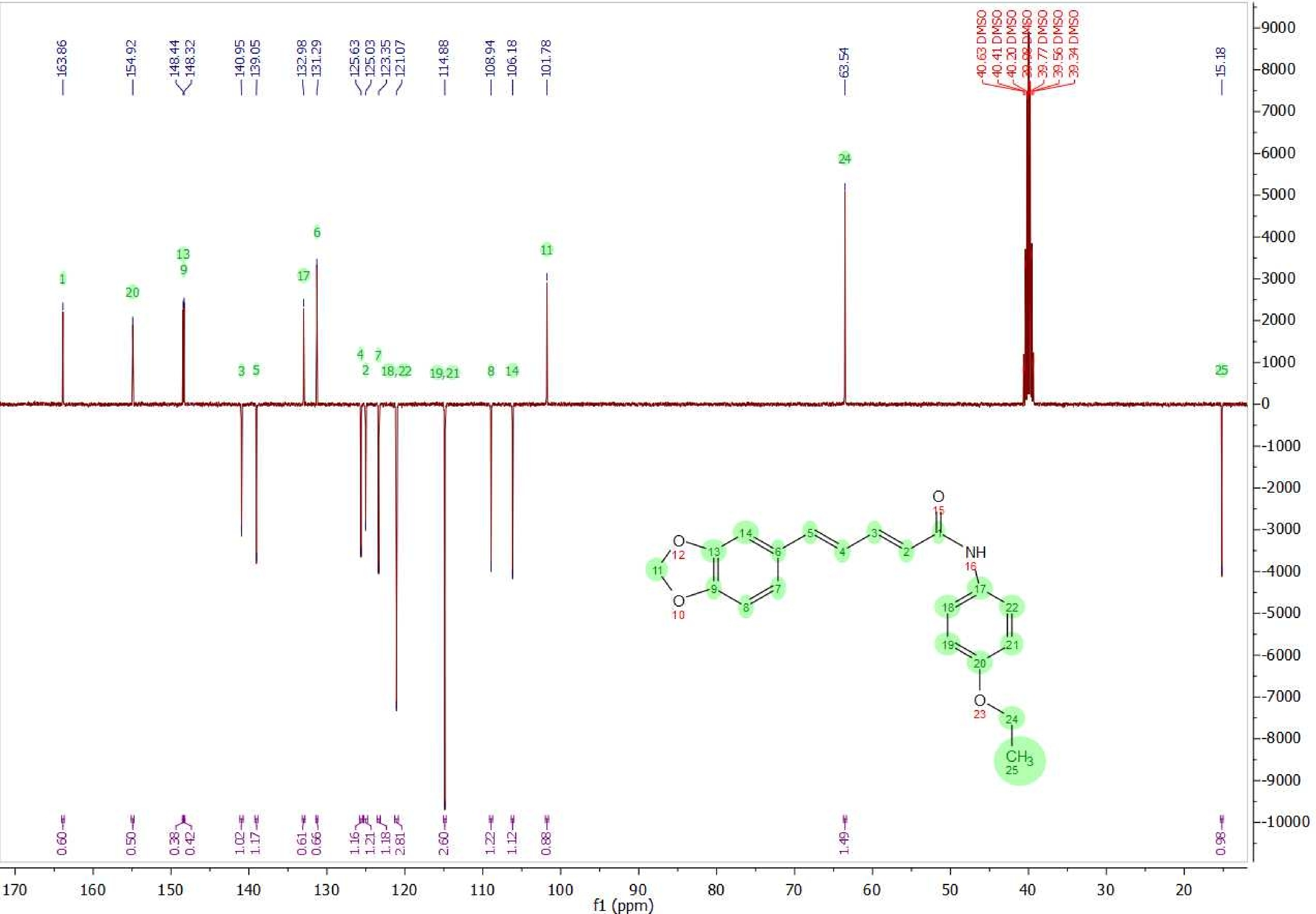

diaa elemam-PAAM-1-Hnmr-OW.10.fid — diaa elemam-PAAM-1-Hnmr-OW

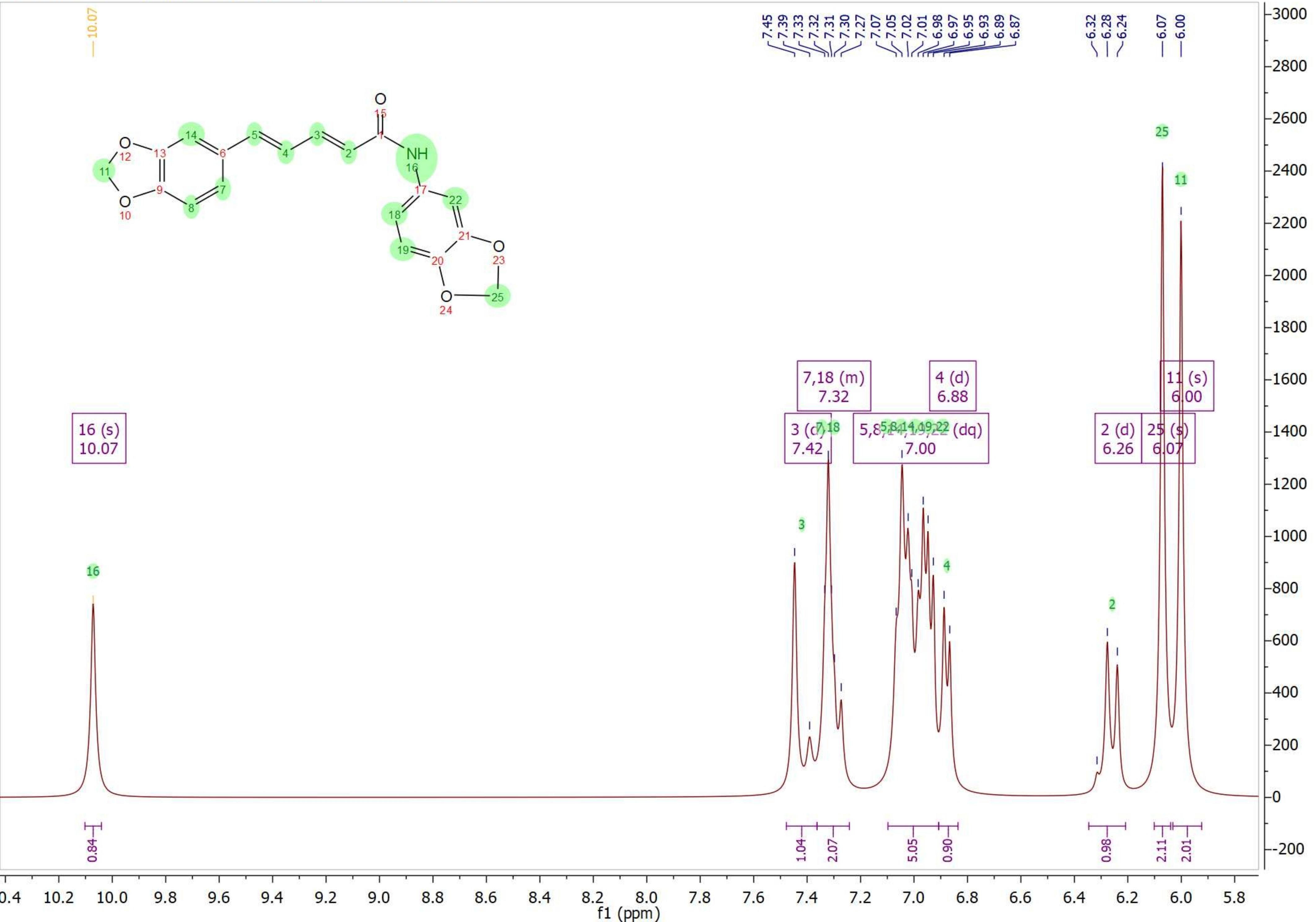

diaa elemam-PAAM-1-APT-OW.10.fid — diaa elemam-PAAM-1-APT-OW

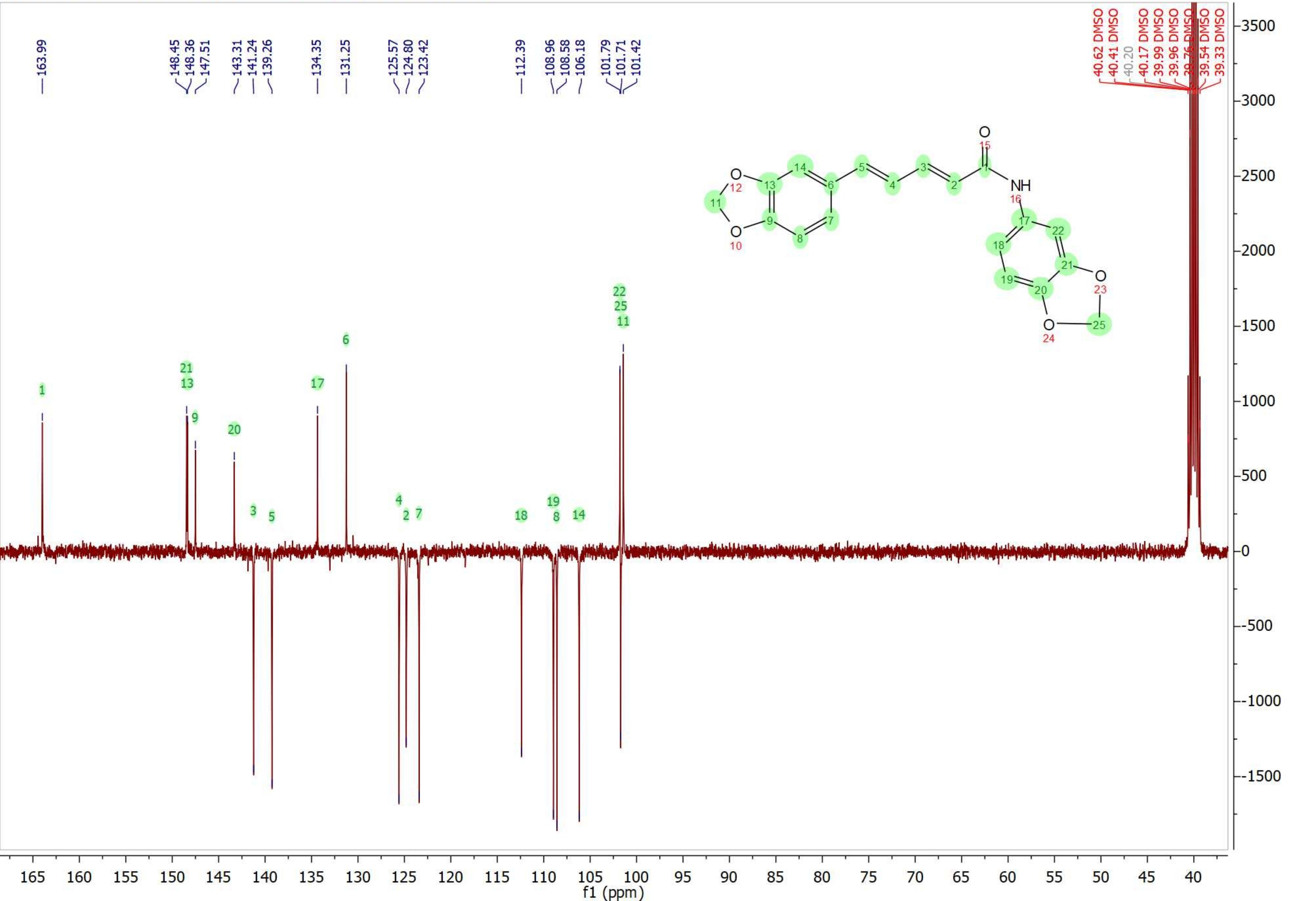

Diaa elemam-An.Pu-Hnmr-ES.10.fid — Diaa elemam-An.Pu-Hnmr-ES

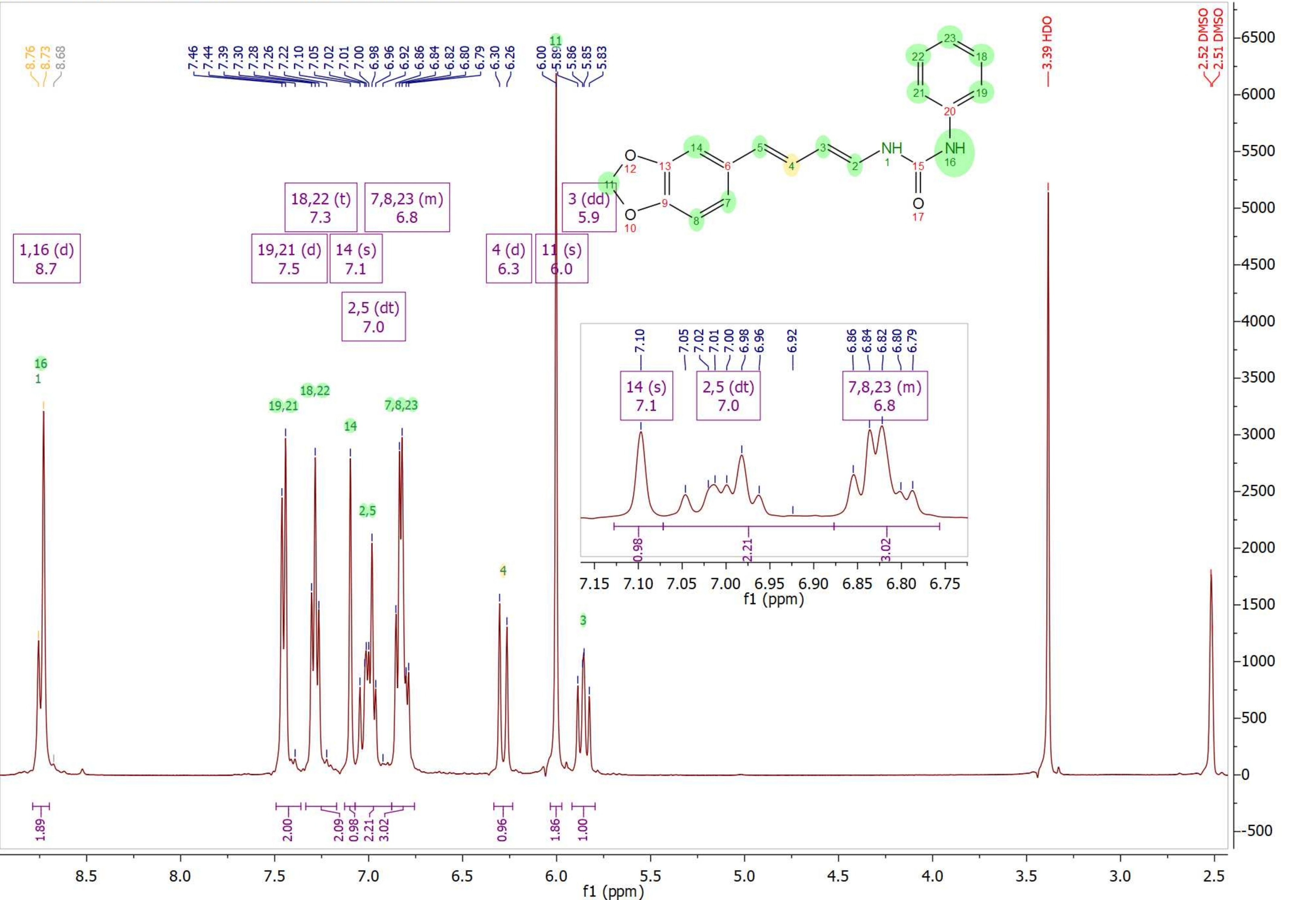

Diaa elemam-An.Pu-APT-ES.10.fid — Diaa elemam-An.Pu-APT-ES

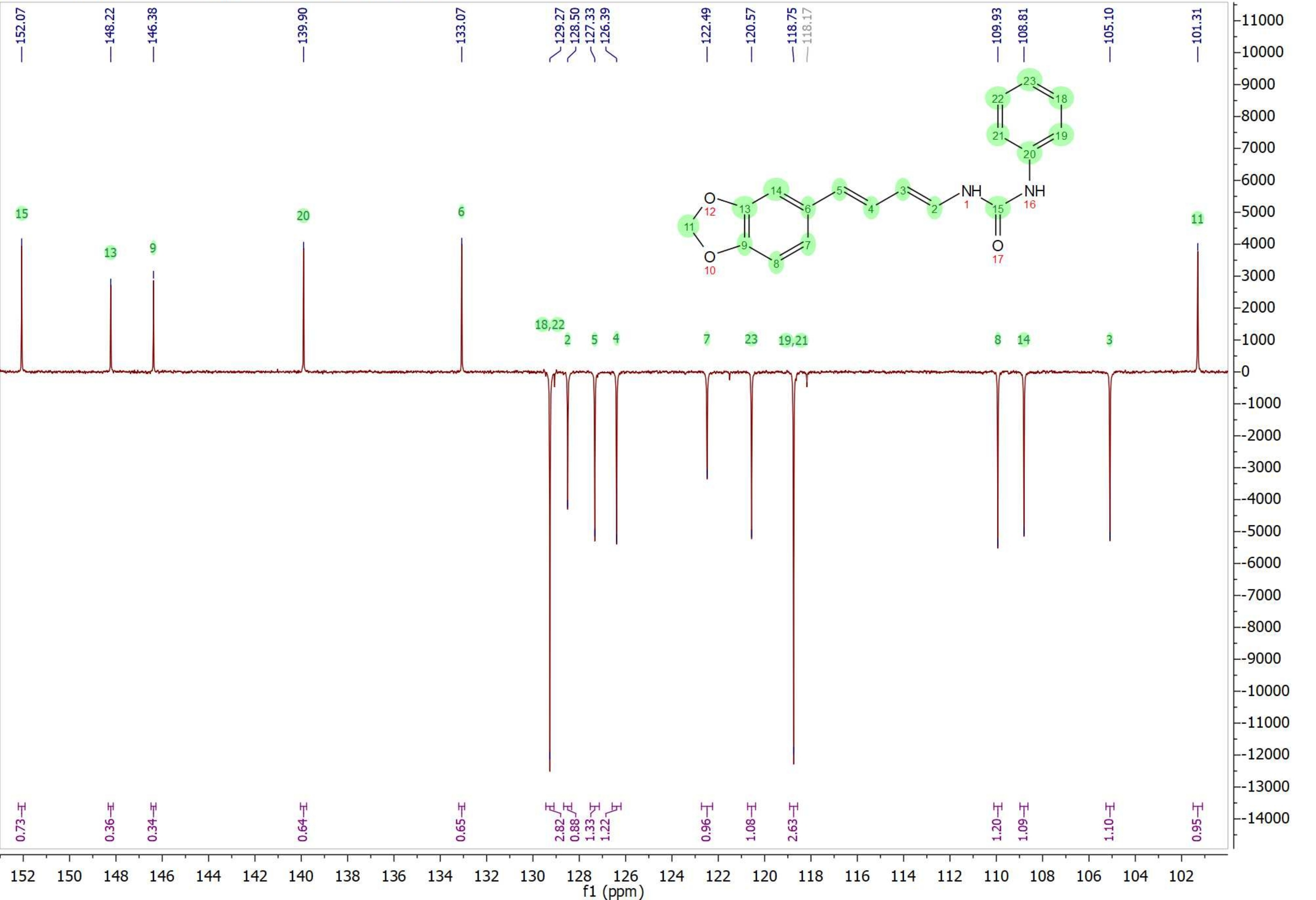

Diaa elemam-3Br-An-Pu-Hnmr-R.10.fid — Diaa elemam-3Br-An-Pu-Hnmr-R

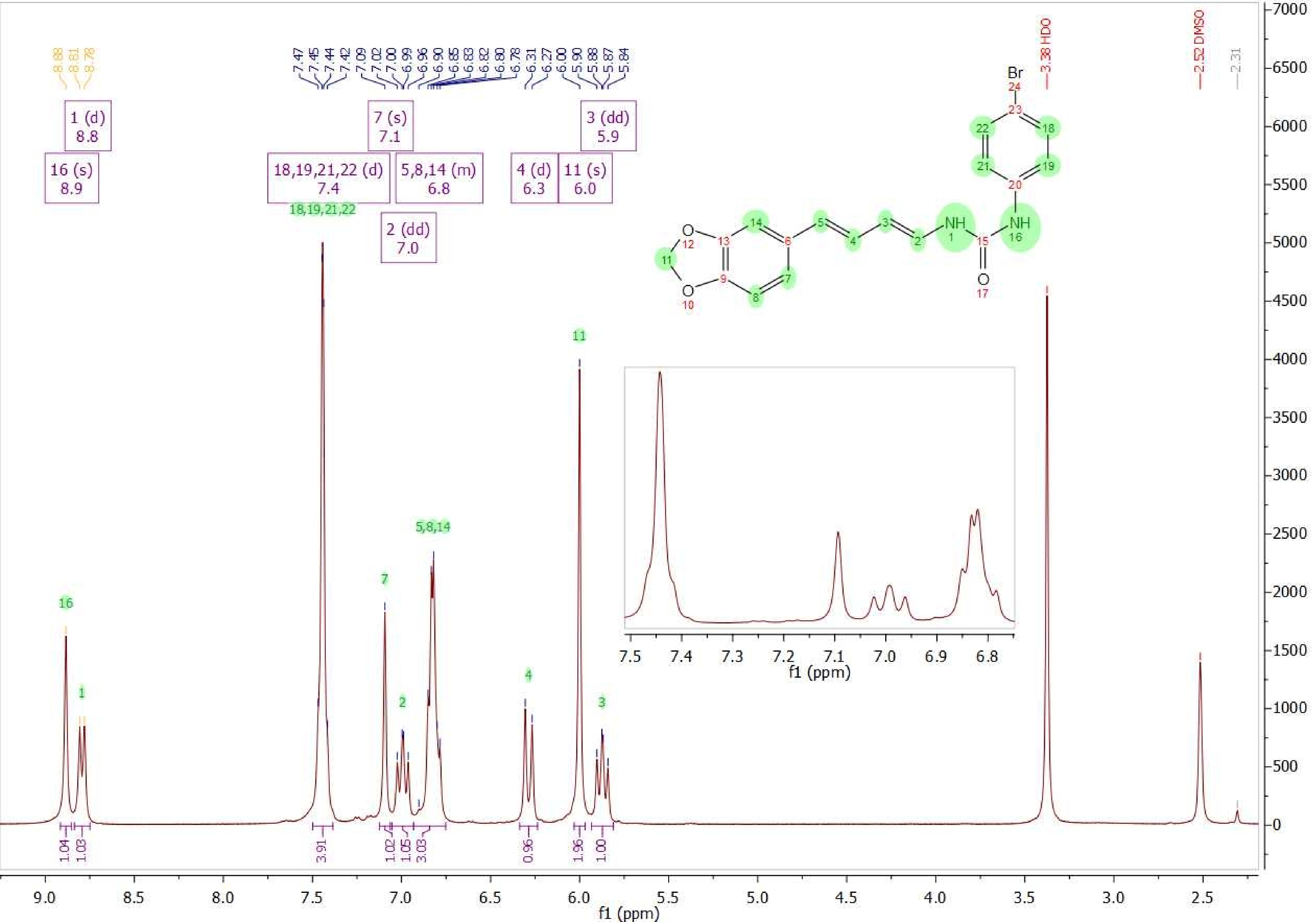

Diaa elemam-3Br-An-Pu-APT-R.10.1.1r — Diaa elemam-3Br-An-Pu-APT-R

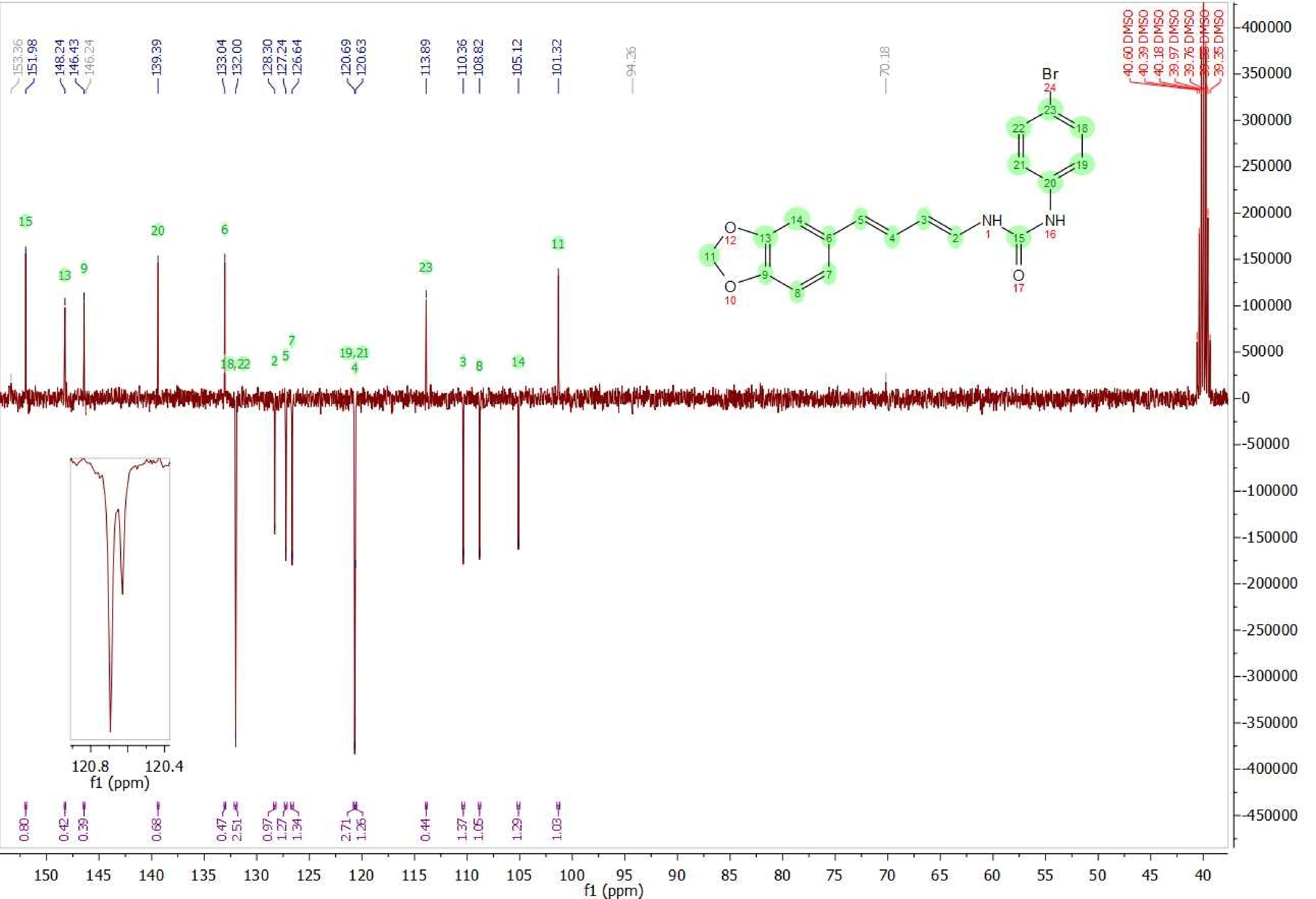

Diaa elemam-3CL.An.Pu-Hnmr-ES.21.fid — Diaa elemam-3CL.An.Pu-Hnmr-ES

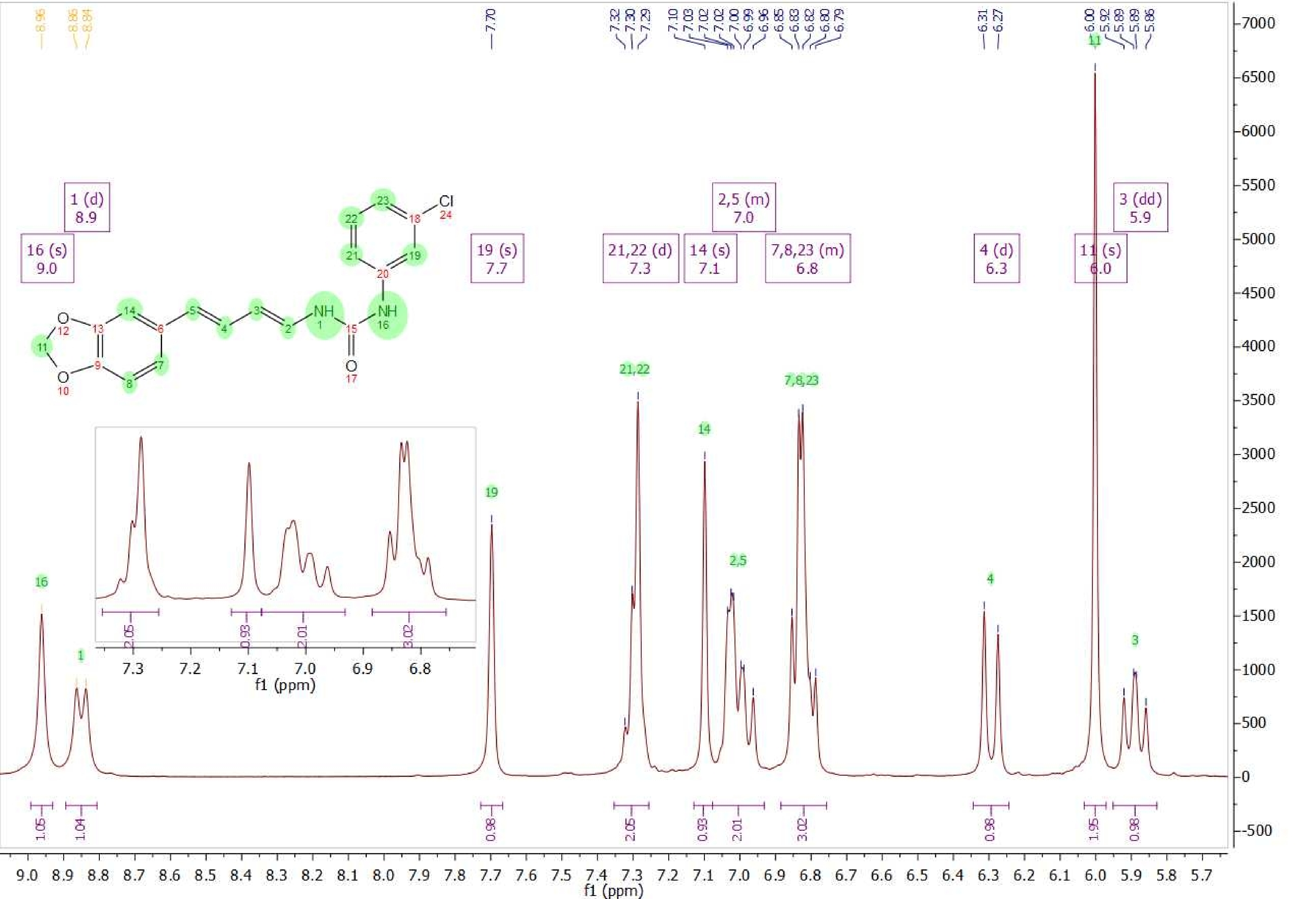

Diaa elemam-3.CL.An.Pu-APT-R.10.fid — Diaa elemam-3.CL.An.Pu-APT-R

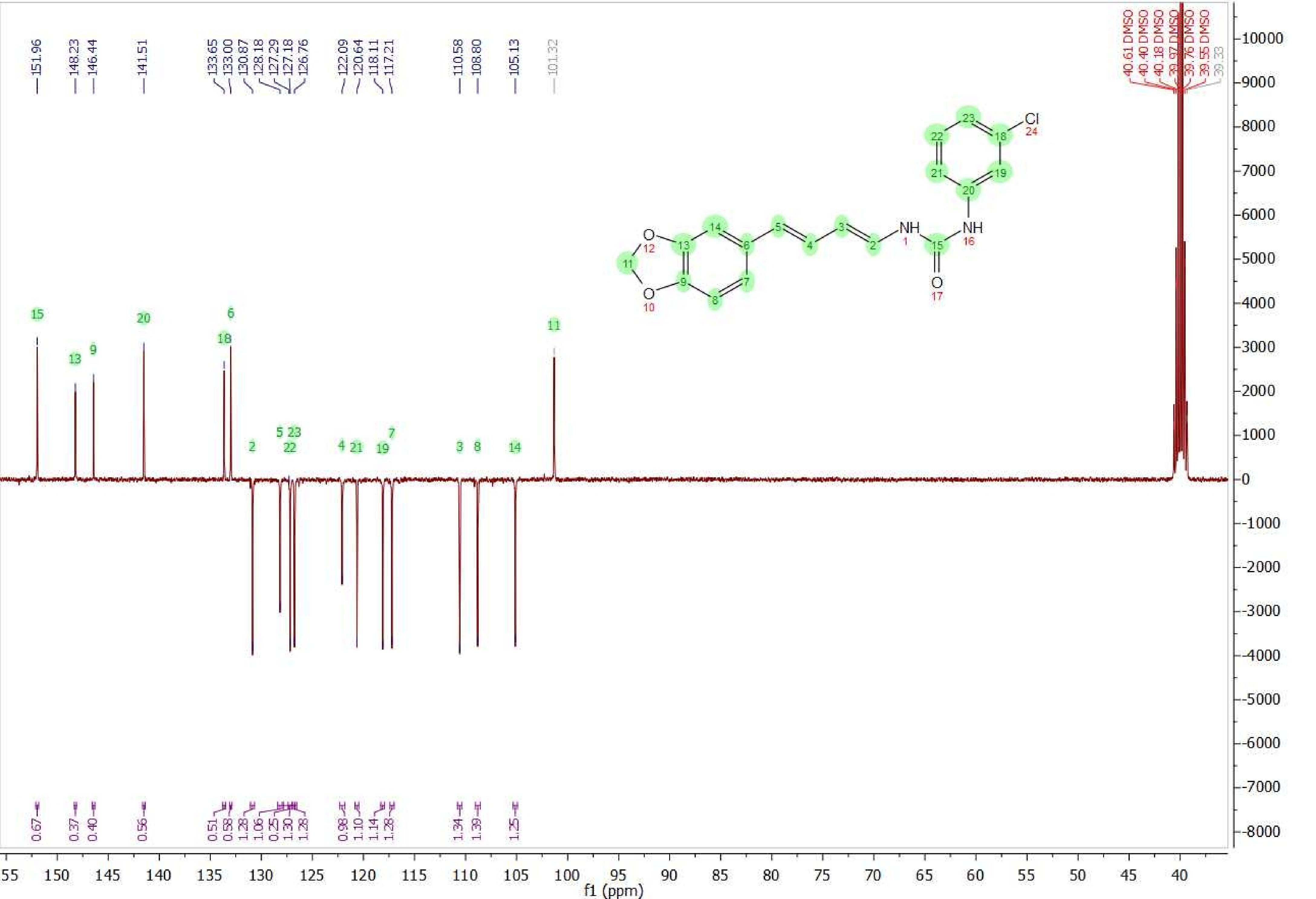

Diaa elemam-P.CL.An.Pu-Hnmr-ES.10.fid — Diaa elemam-P.CL.An.Pu-Hnmr-ES

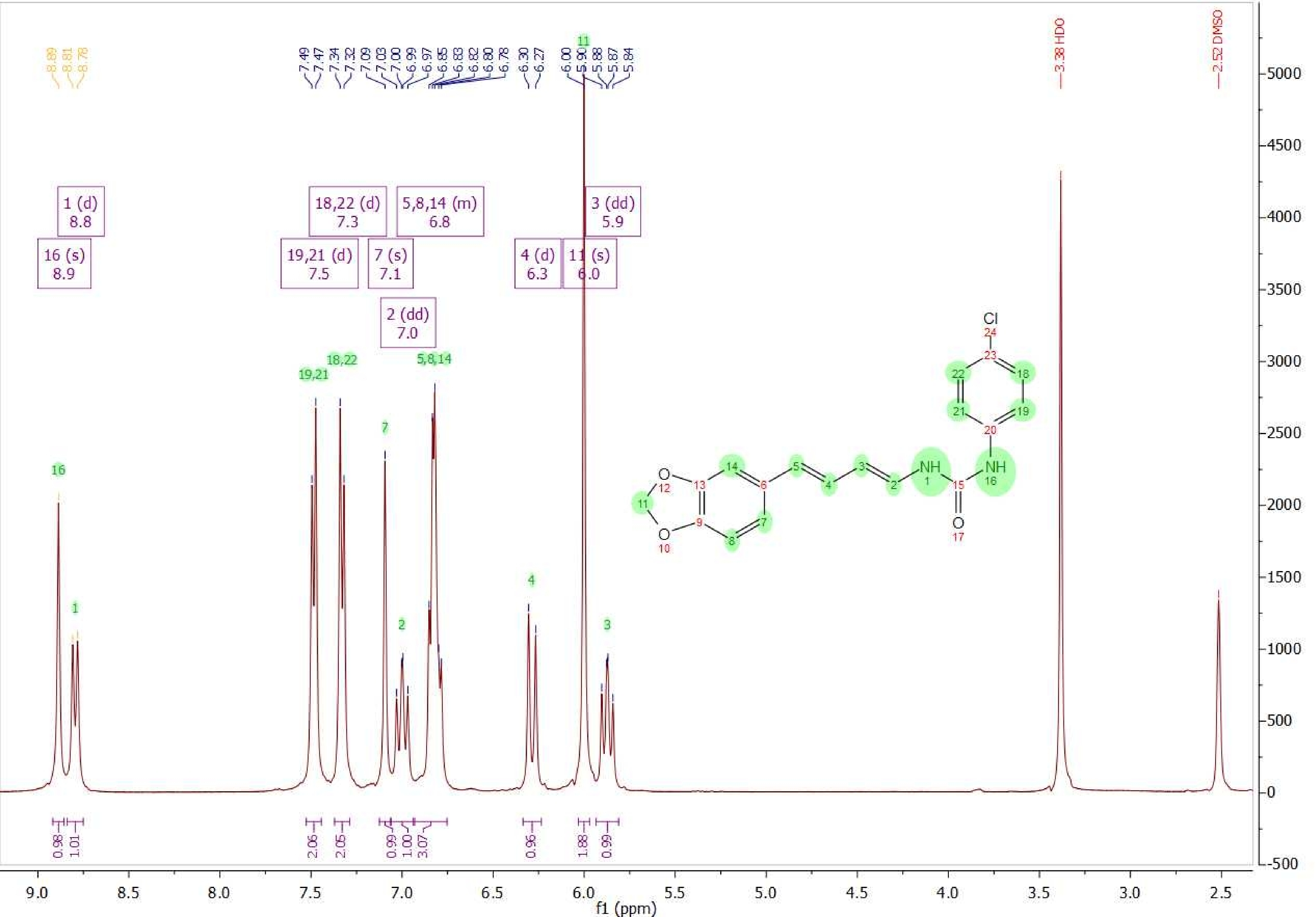

Diaa elemam-P.CL.An.Pu-APT-ES.10.fid — Diaa elemam-P.CL.An.Pu-APT-ES

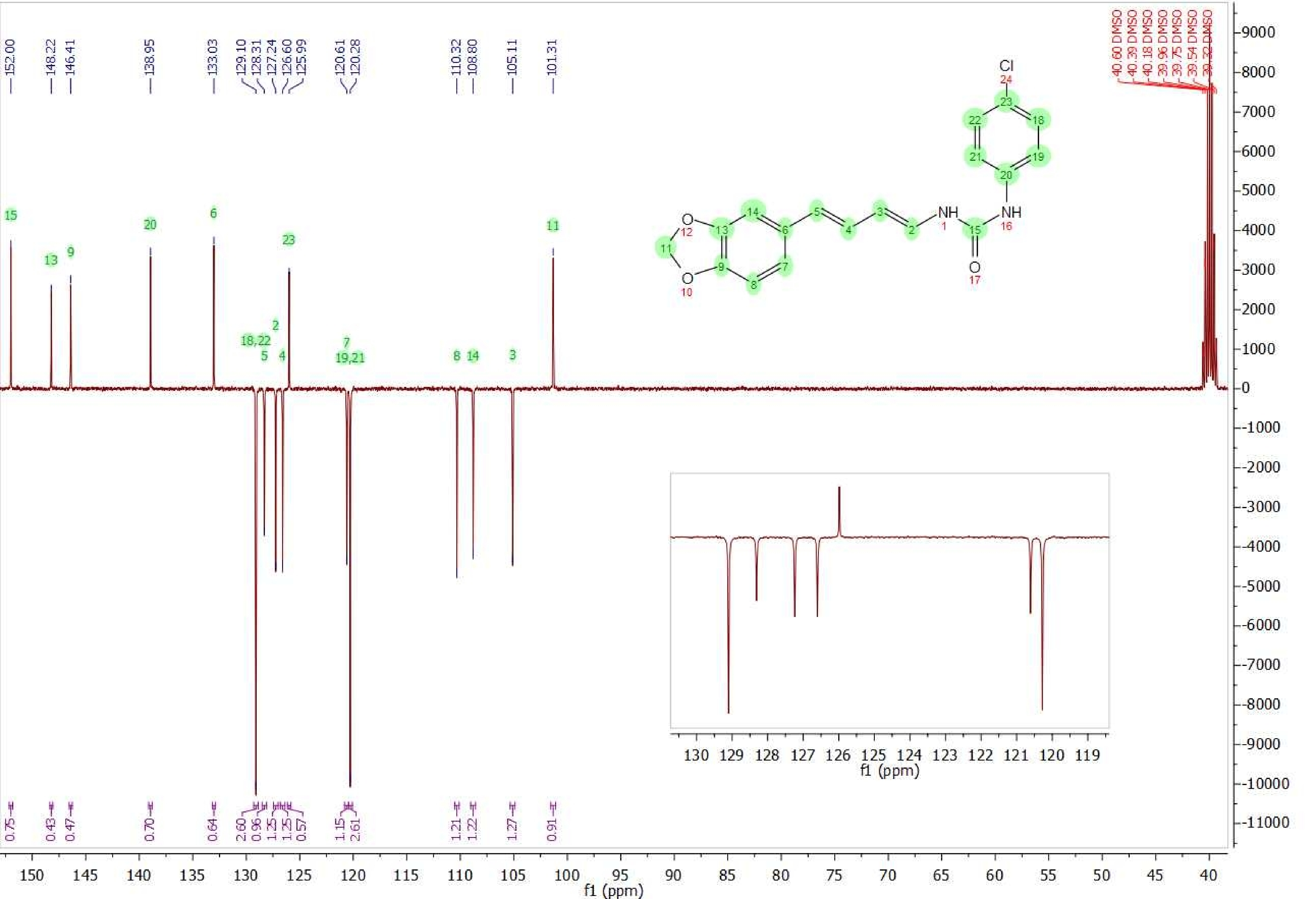

Diaaeldin Elimam-F An P-DMSO-Hnmr-A.10.fid — Diaaeldin Elimam-F An P-DMSO-Hnmr-A

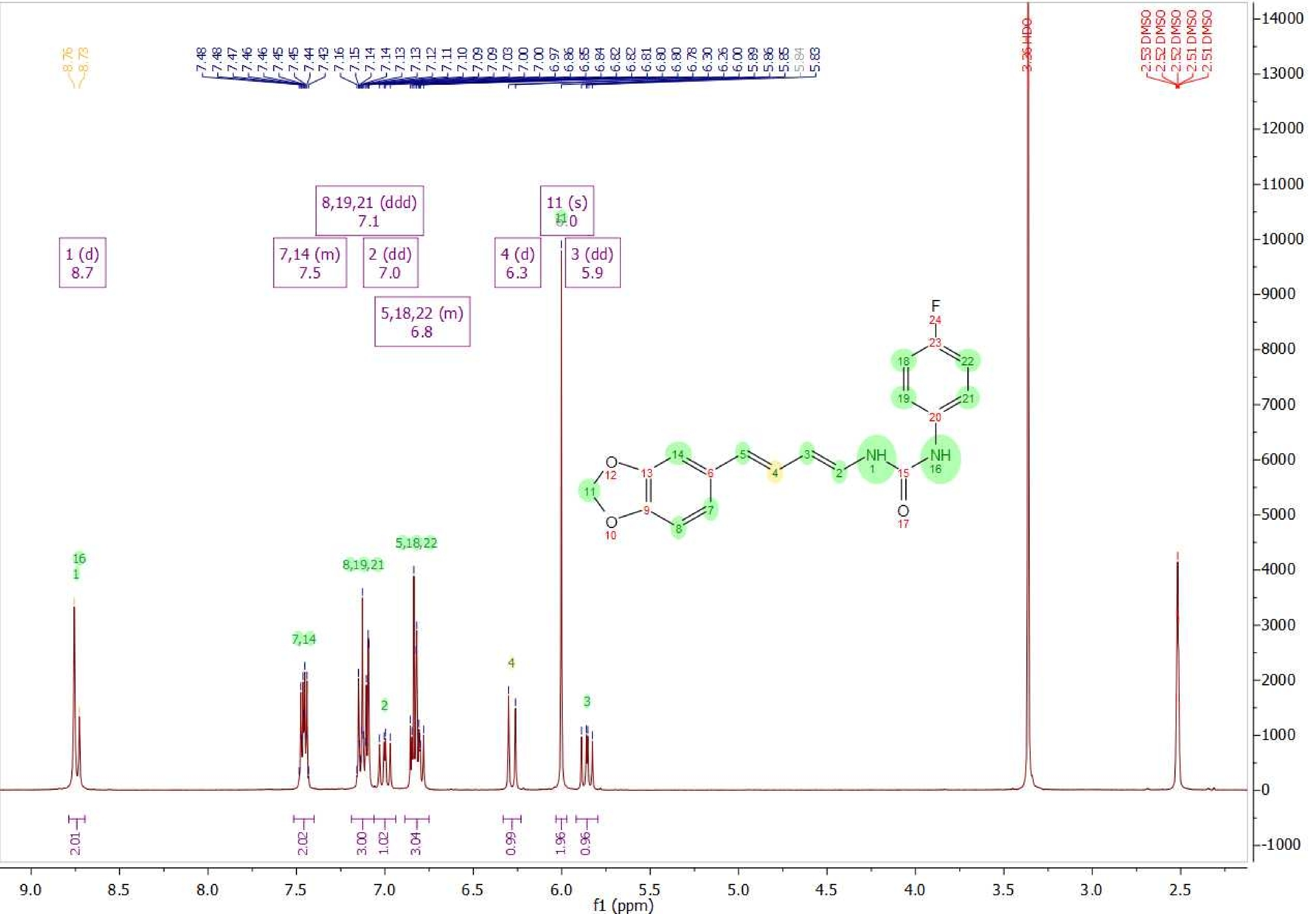

Diaa elemam-4.F.An.P4-APT-ES.10.fid — Diaa elemam-4.F.An.P4-APT-ES

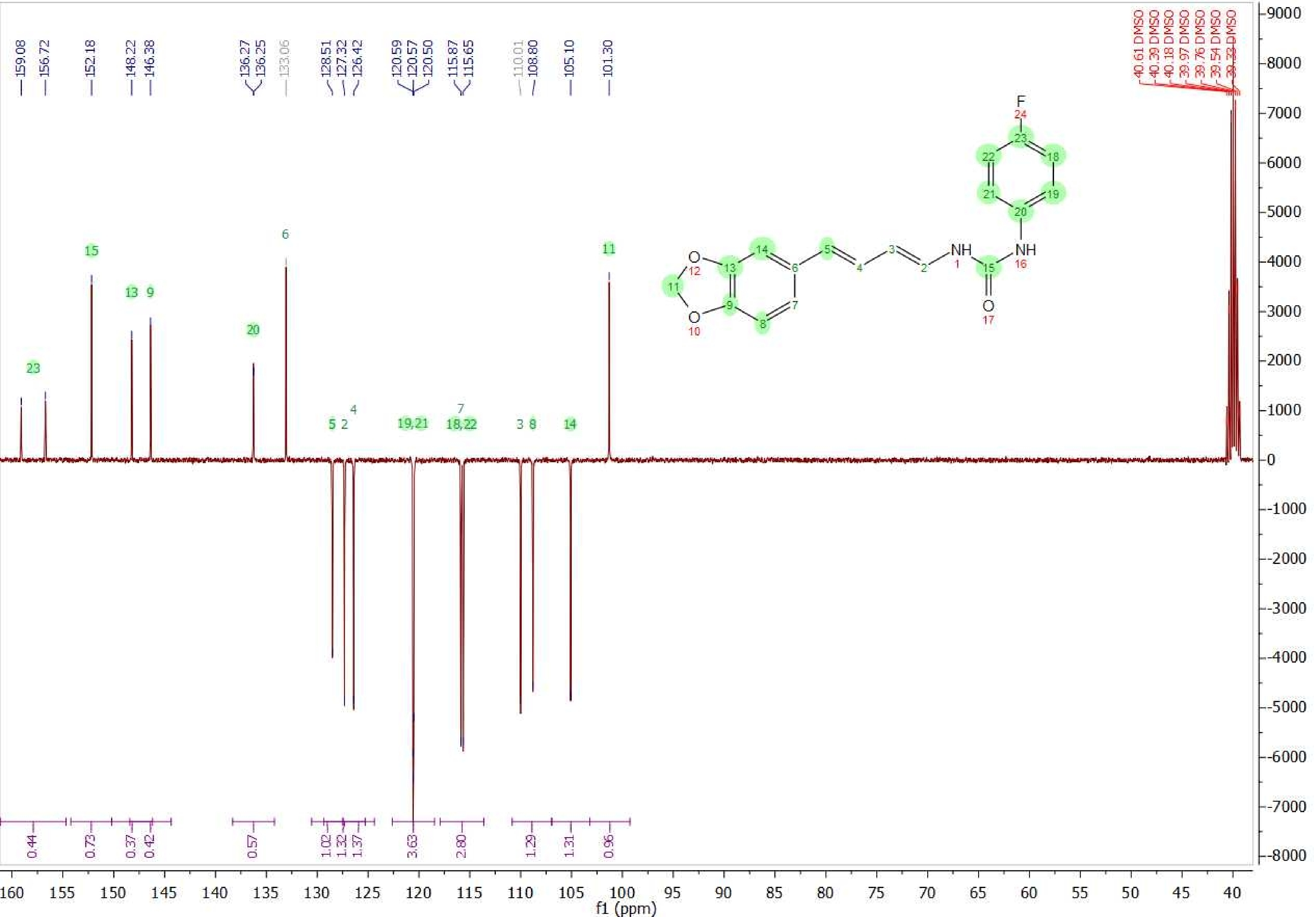

Diaa elemam-4.CF3.An.Pu-Hnmr-ES.10.fid — Diaa elemam-4.CF3.An.Pu-Hnmr-ES

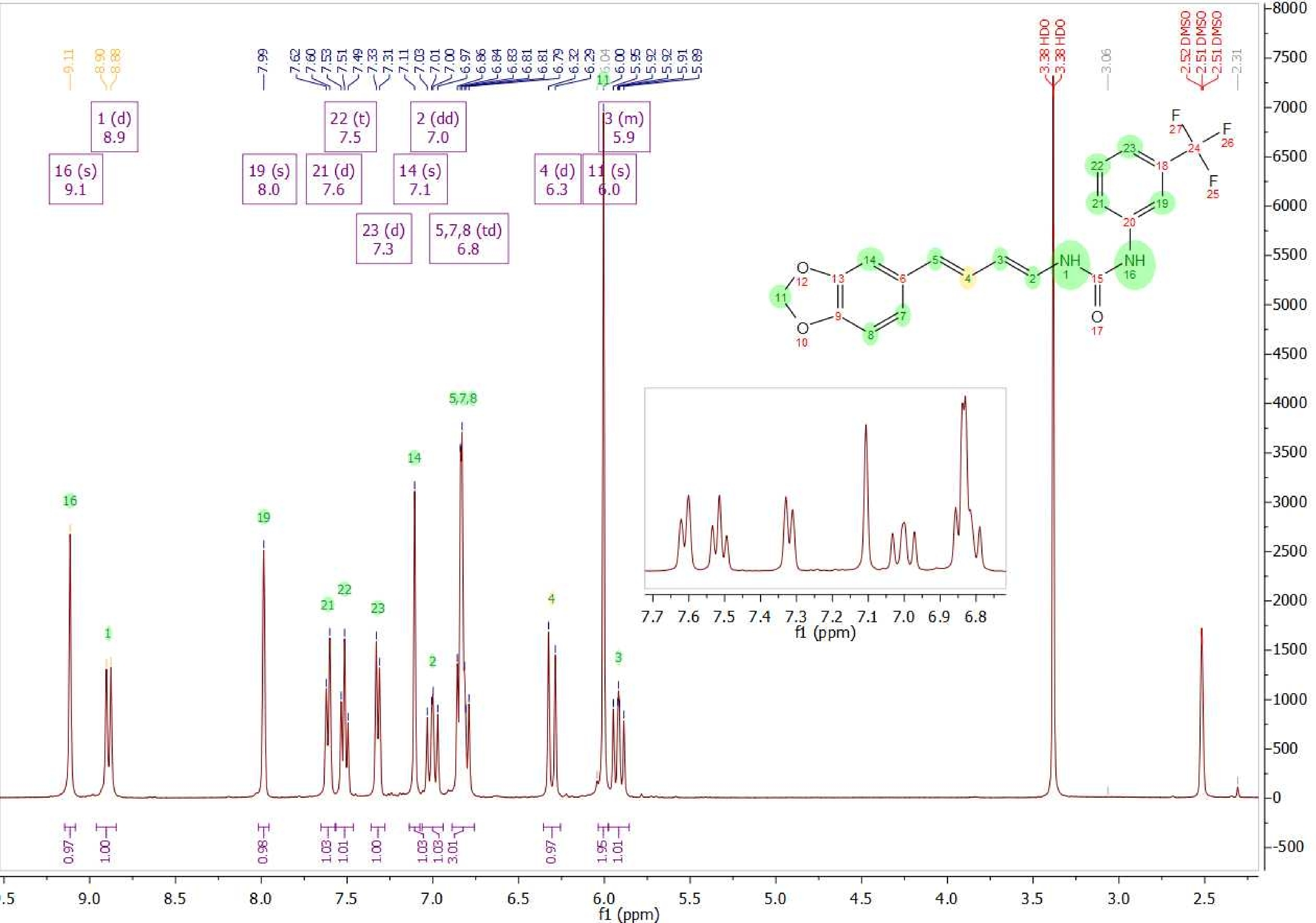

Diaa elemam-4.CF3.An.Pu-APT-R.10.fid — Diaa elemam-4.CF3.An.Pu-APT-R

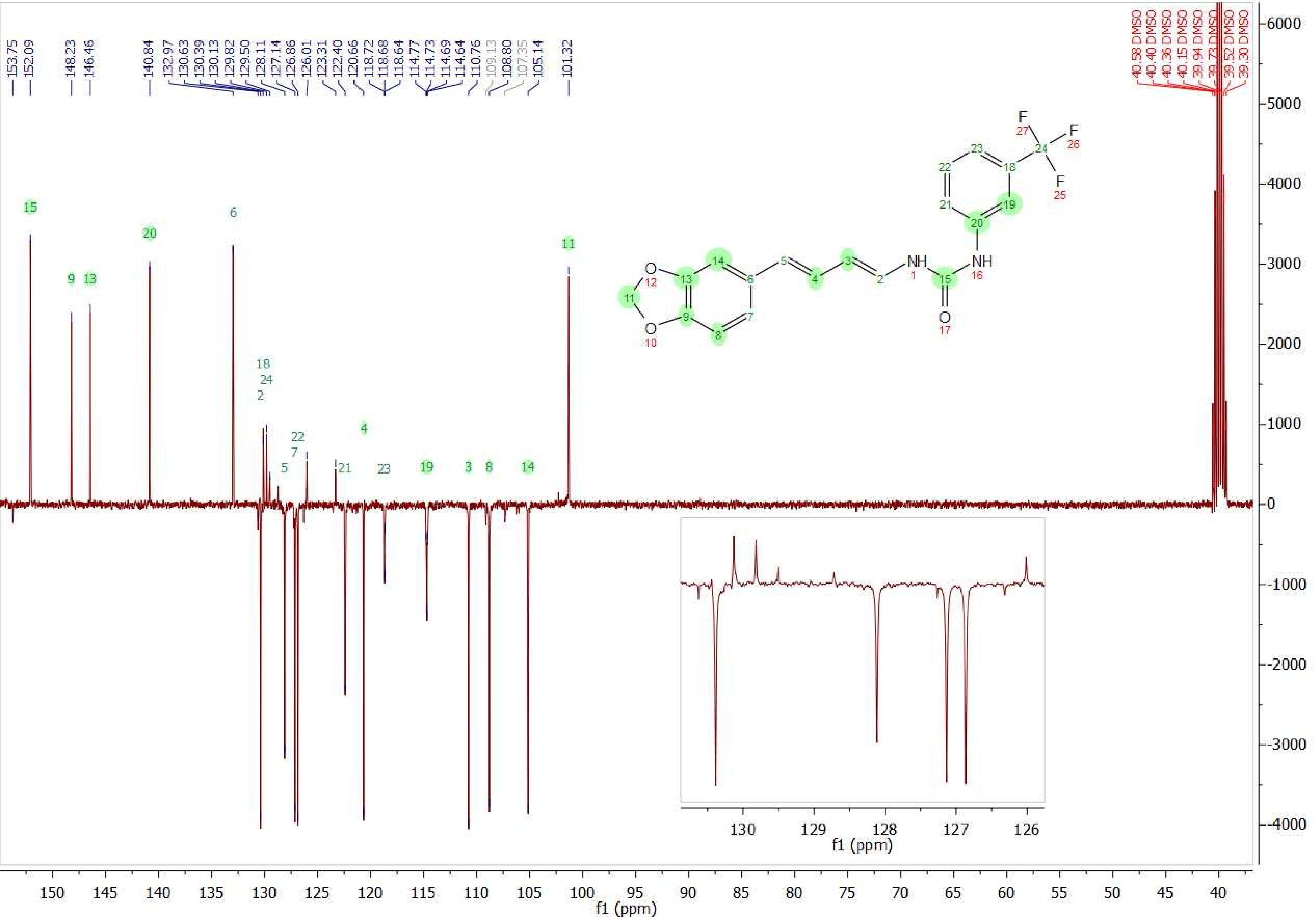

Diaaeldin Elimam-Br F An P-DMSO-Hnmr-A.10.fid — Diaaeldin Elimam-Br F An P-DMSO-Hnmr-A

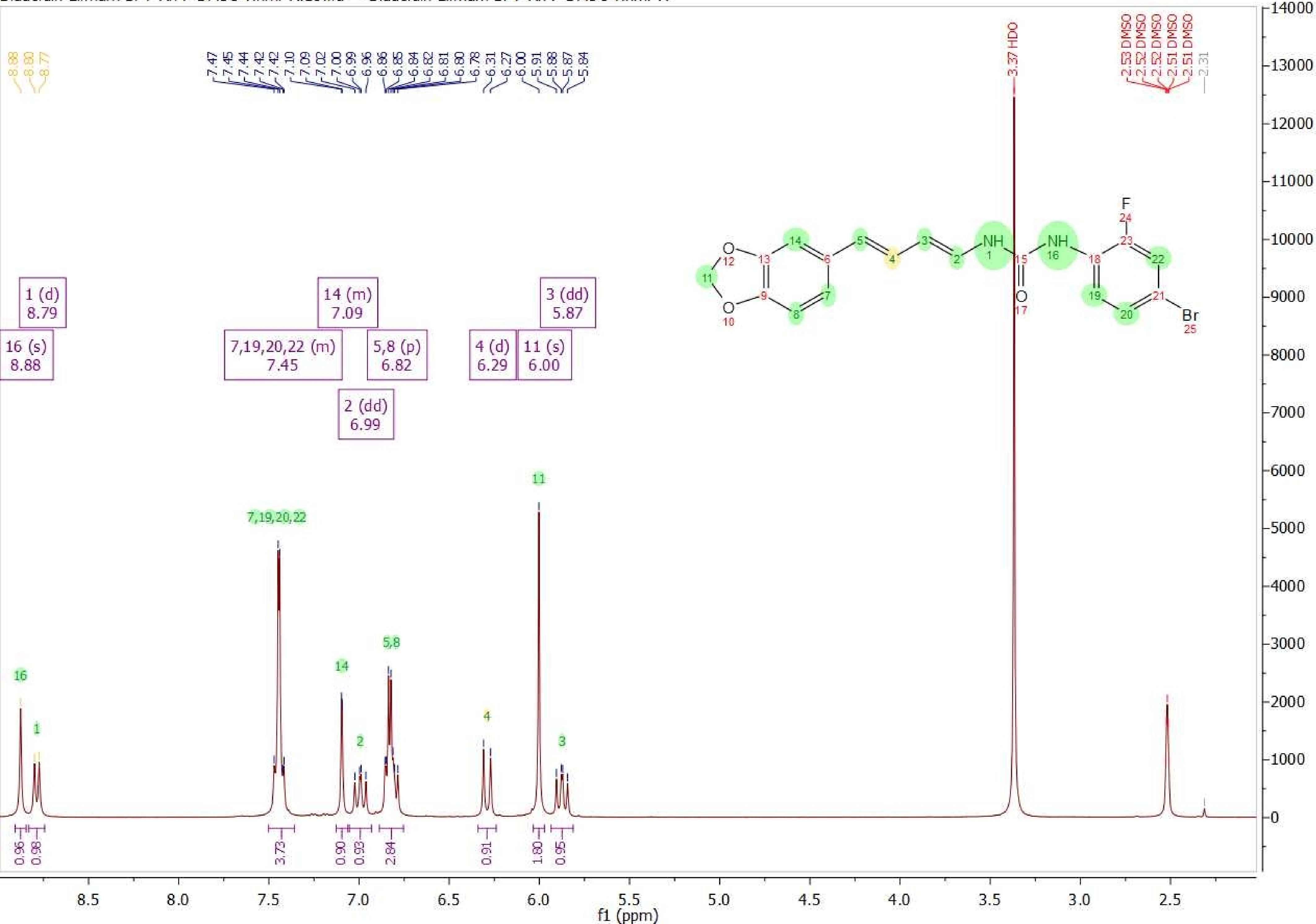

Diaa elemam-BC-Pu-Hnmr-ES.10.fid — Diaa elemam-BC-Pu-Hnmr-ES

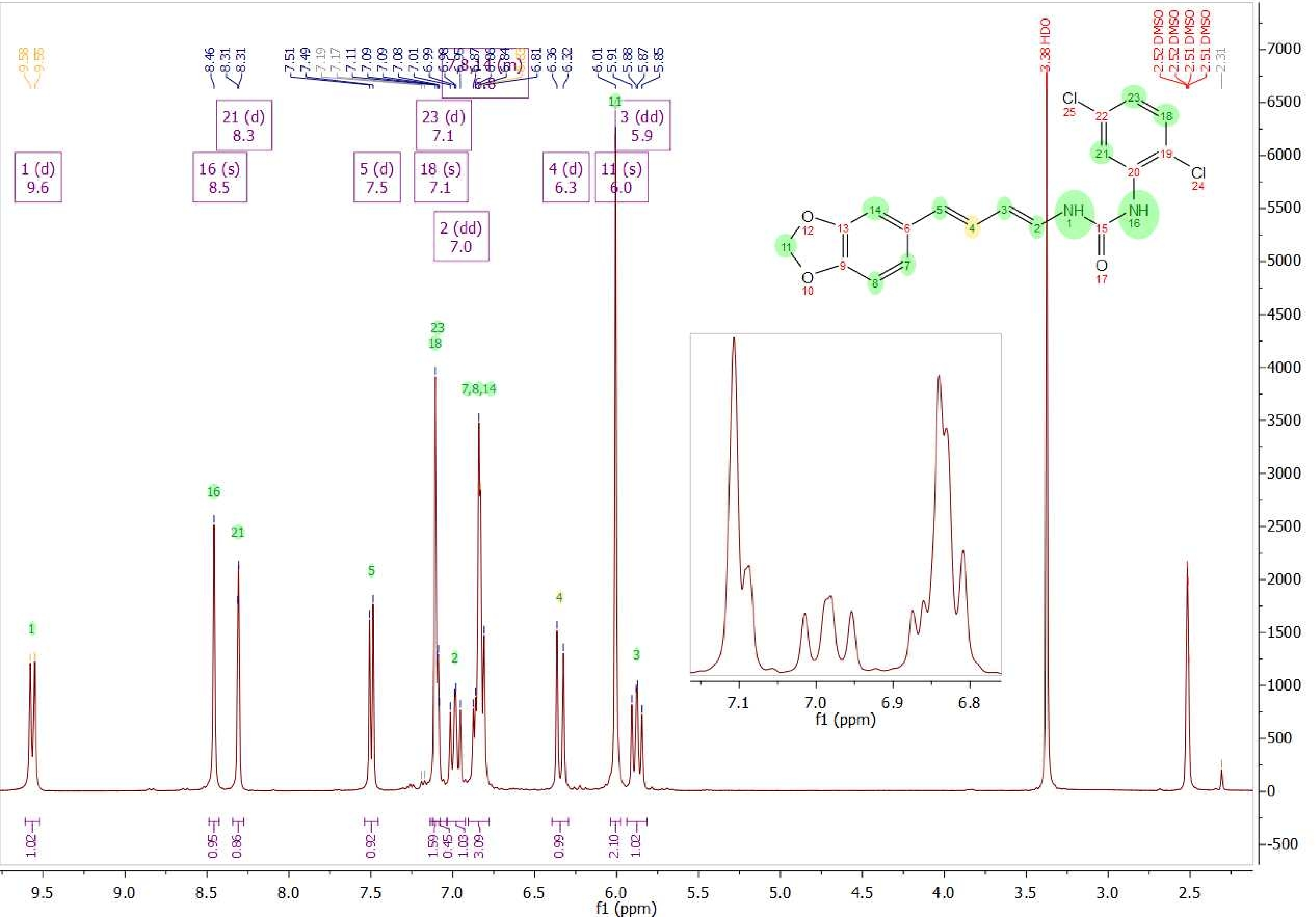

Diaa elemam-BC-Pu-APT-R.10.fid — Diaa elemam-BC-Pu-APT-R

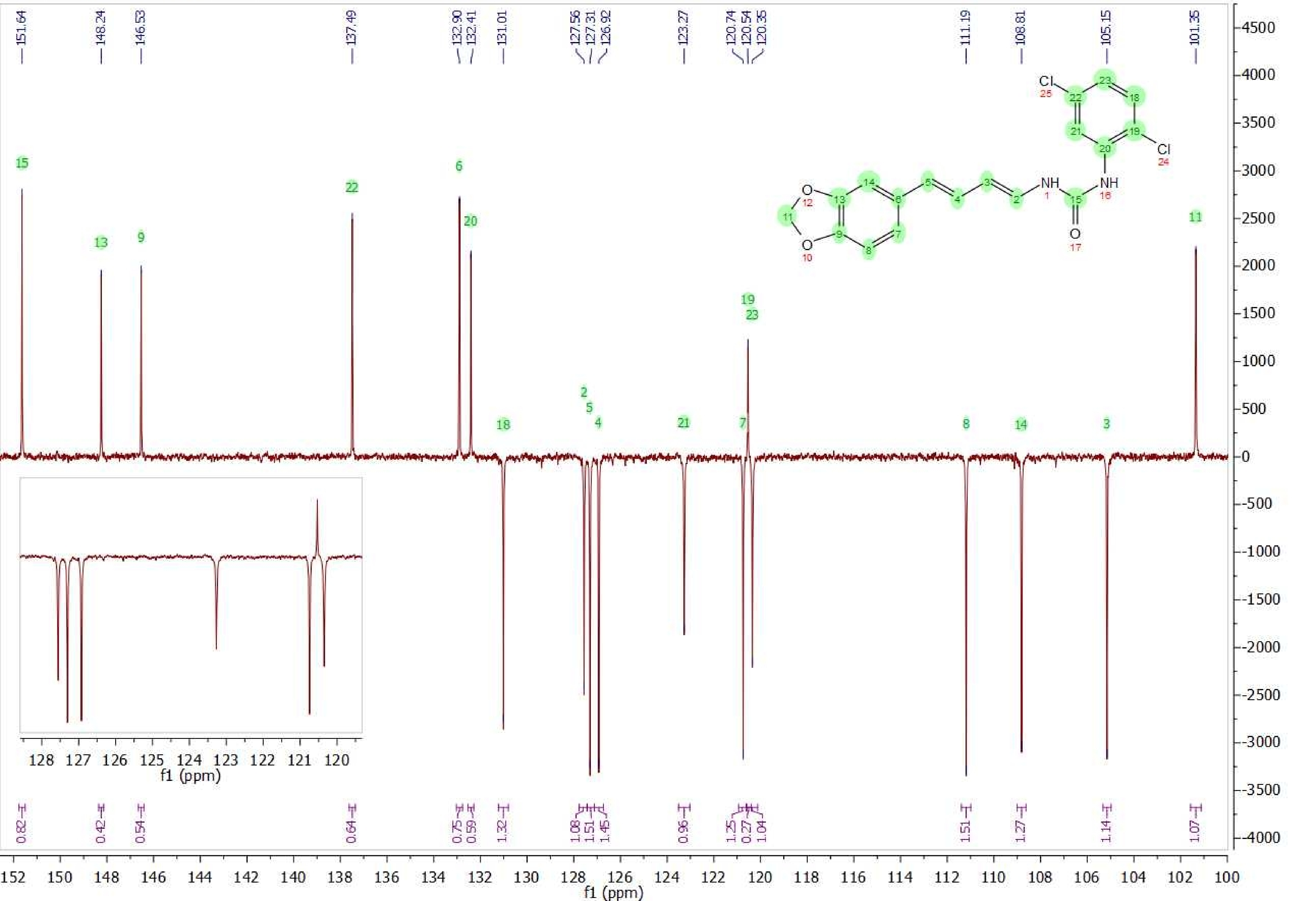

Diaa elemam-PL-3CL.4F.An.PL-Hnmr-ES.10.fid — Diaa elemam-PL-3CL.4F.An.PL-Hnmr-ES

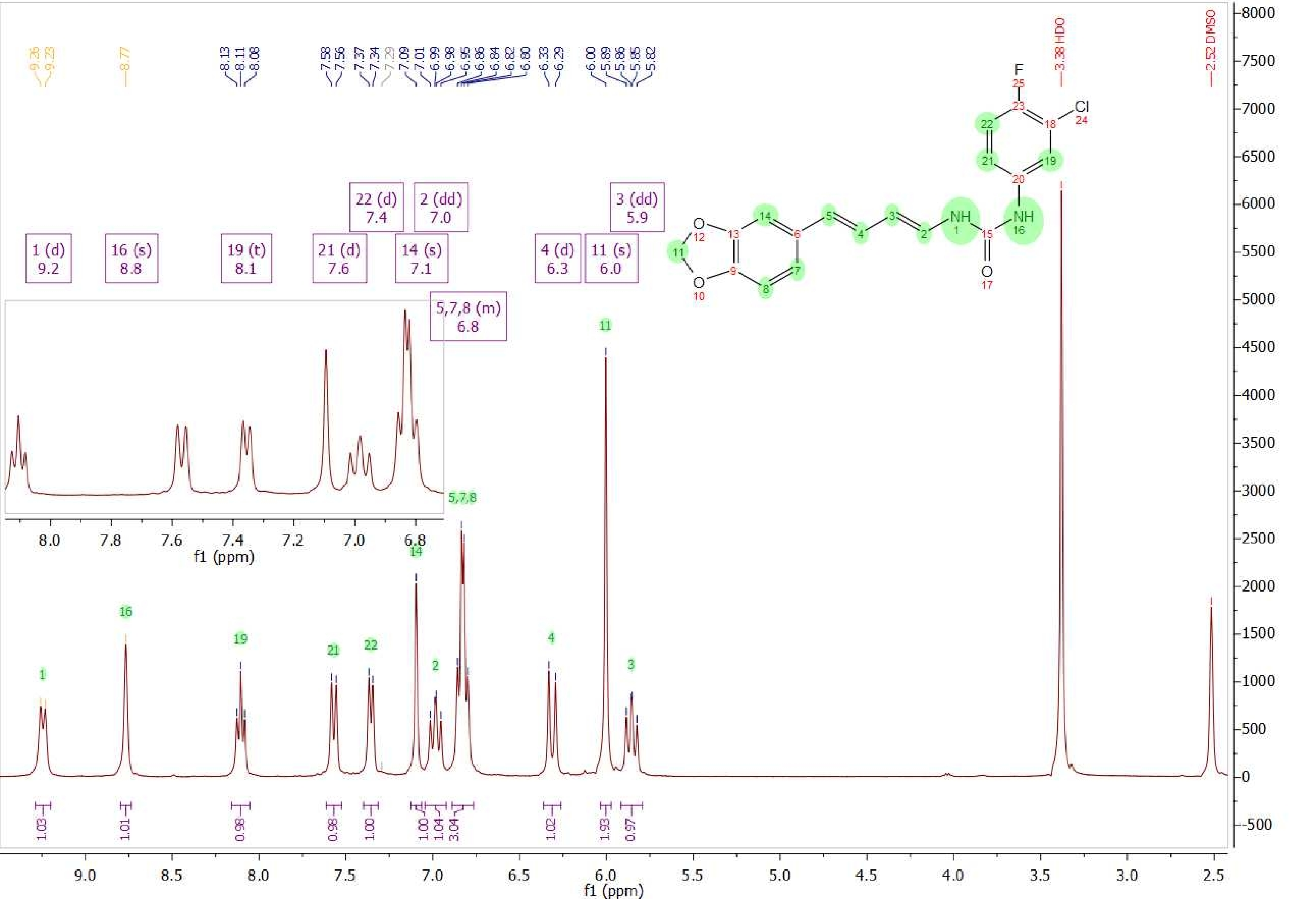

Diaa elemam-PL-3CL.4F.An.PL-APT-ES.10.fid — Diaa elemam-PL-3CL.4F.An.PL-ES

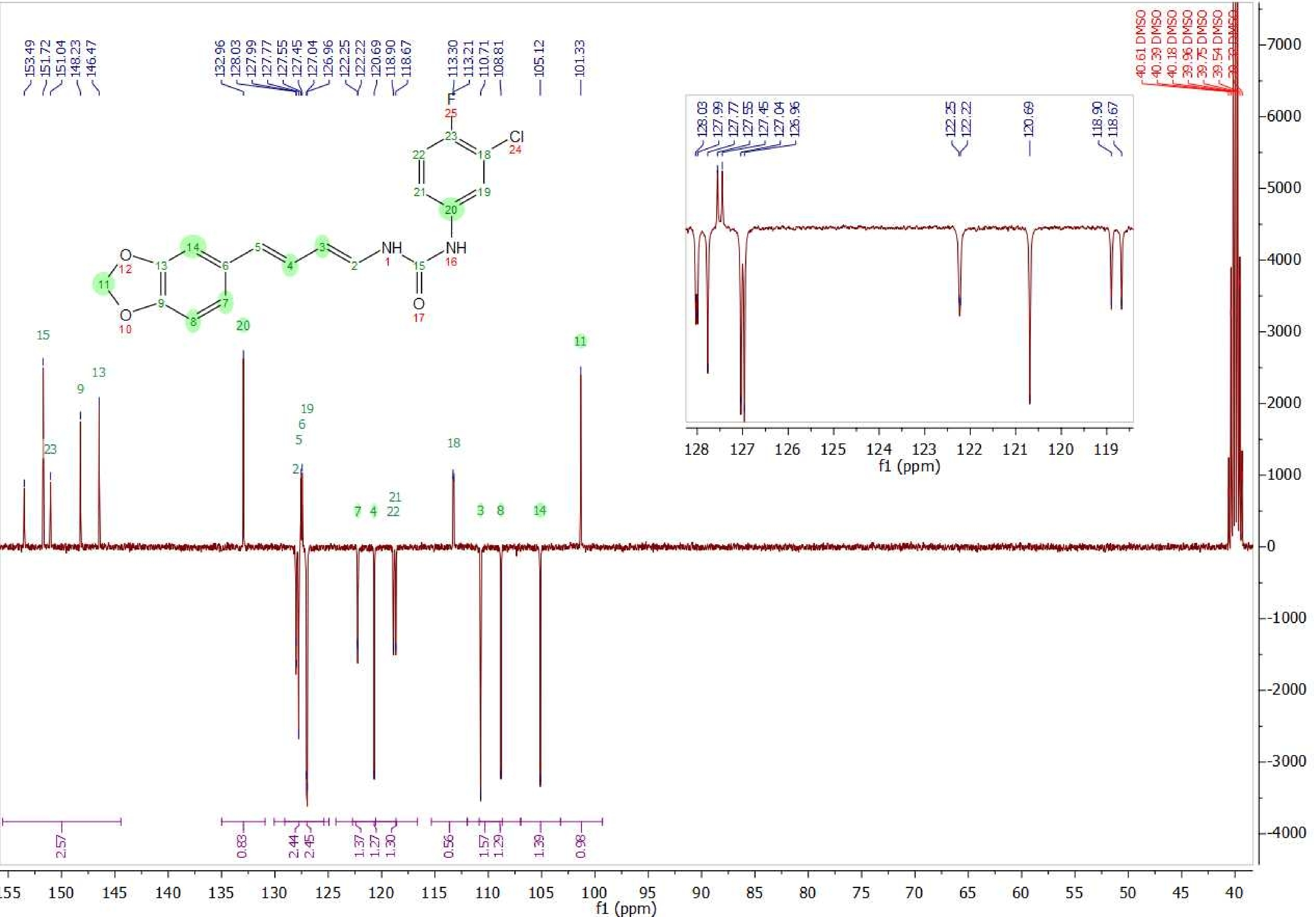

Diaa elemam-4.CL.3CF3.P4-Hnmr-ES.10.fid — Diaa elemam-4.CL.3CF3.P4-Hnmr-ES

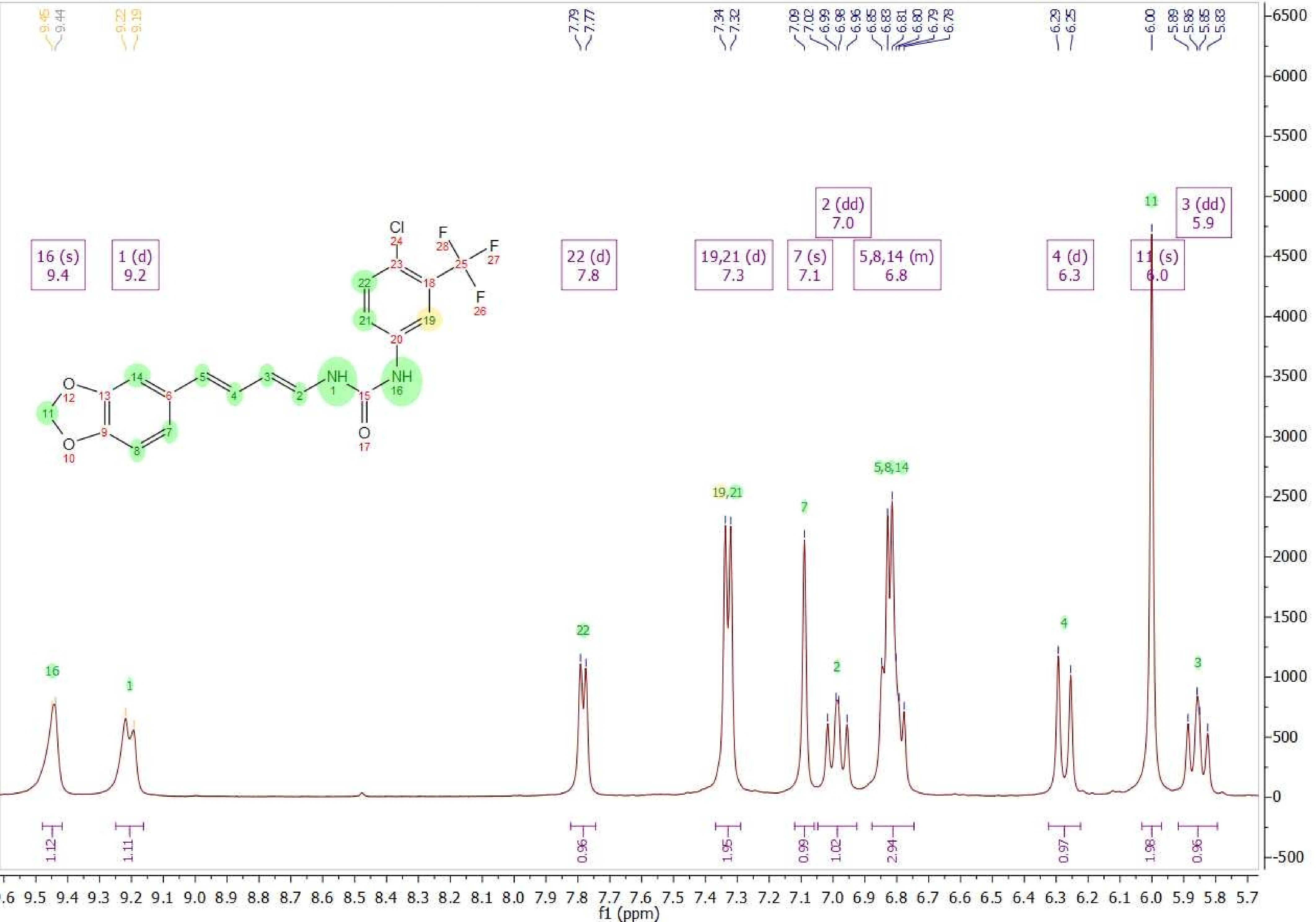

Diaa elemam-4.CL.3CF3.P4-APT-ES.10.fid — Diaa elemam-4.CL.3CF3.P4-APT-ES

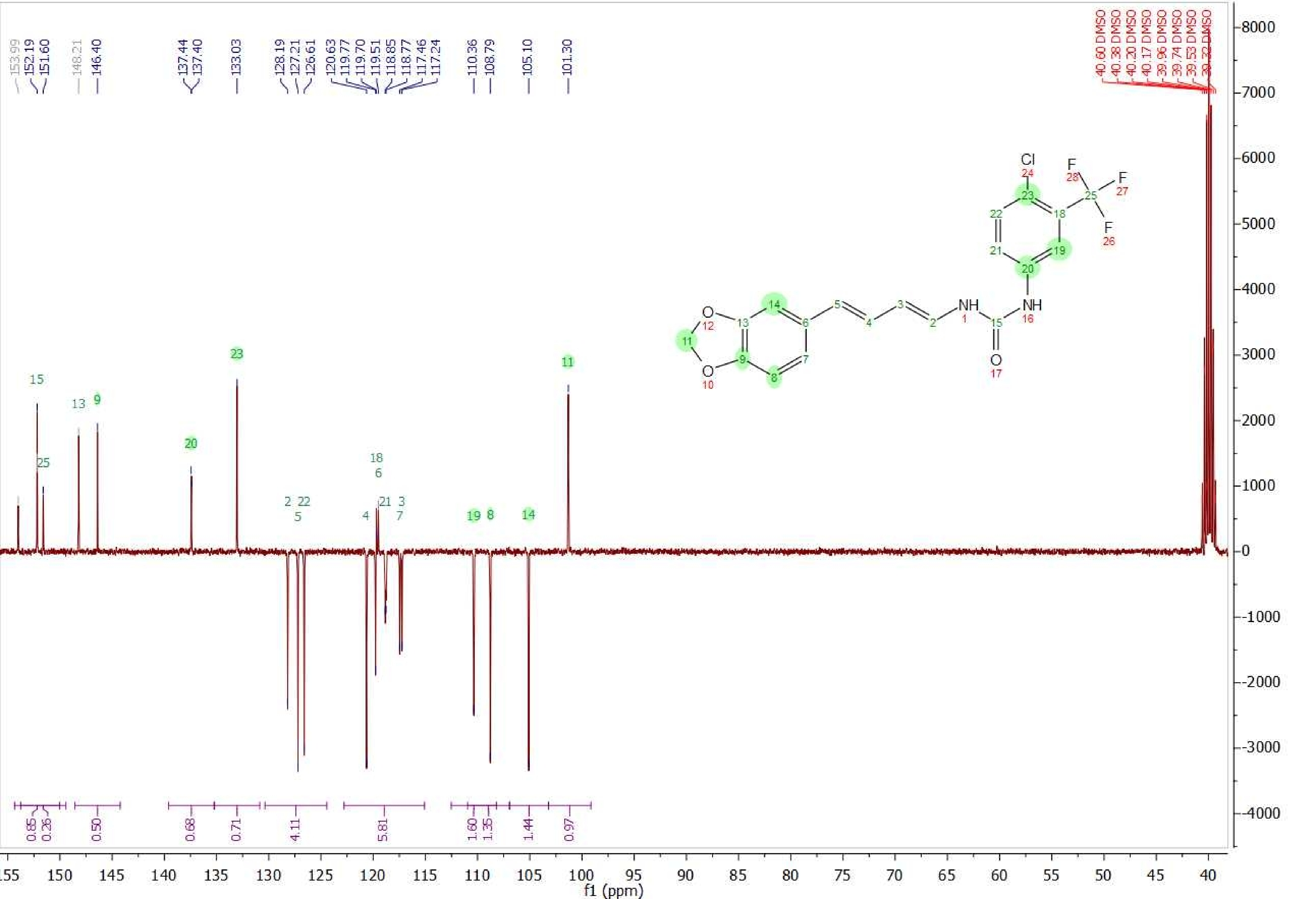

Diaa elemam-Tol.p.u-Hnmr-R.10.fid — Diaa elemam-Tol.p.u-Hnmr-R

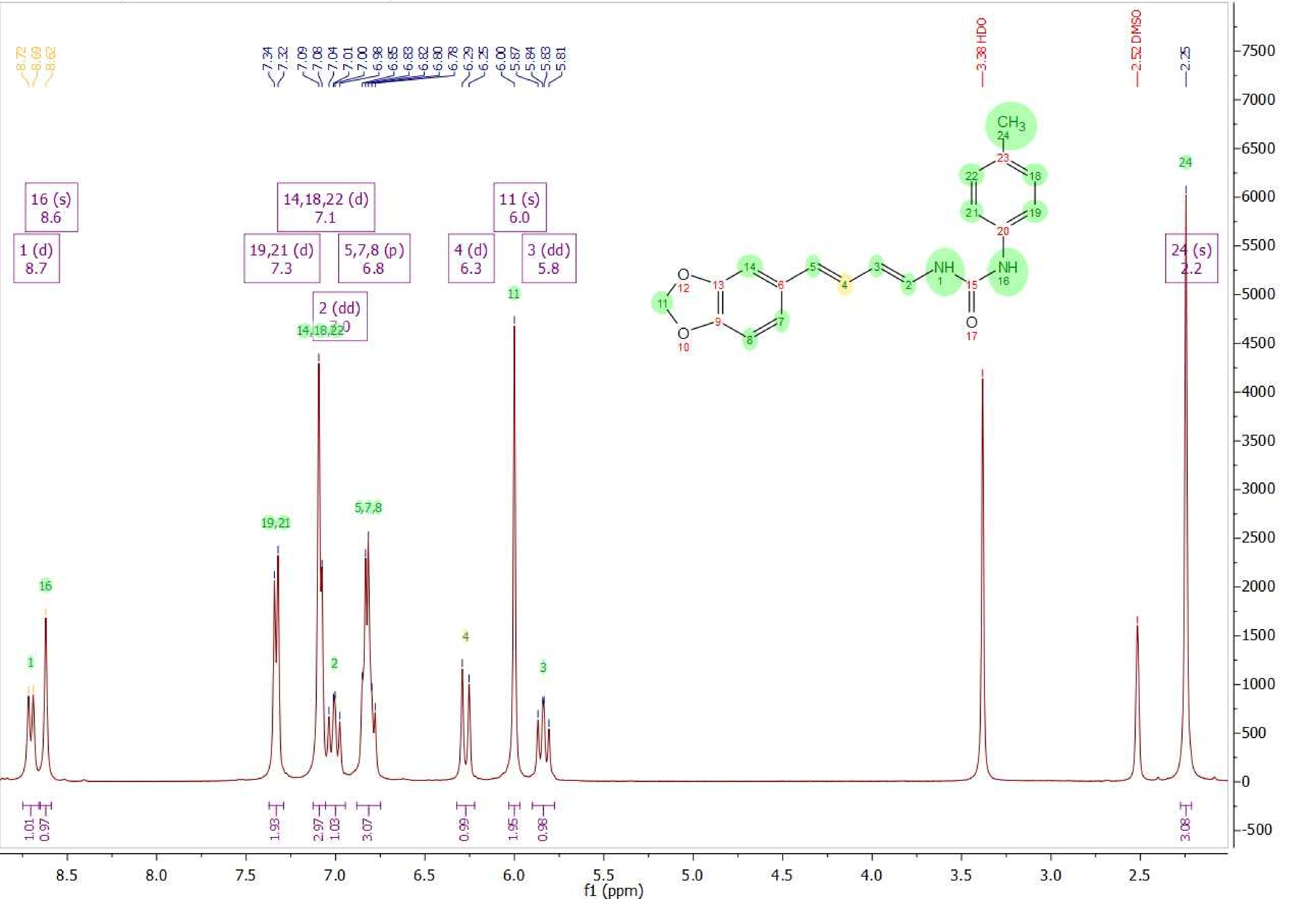

Diaa elemam-Tol.p.u-APT-R.10.fid — Diaa elemam-Tol.p.u-APT-R

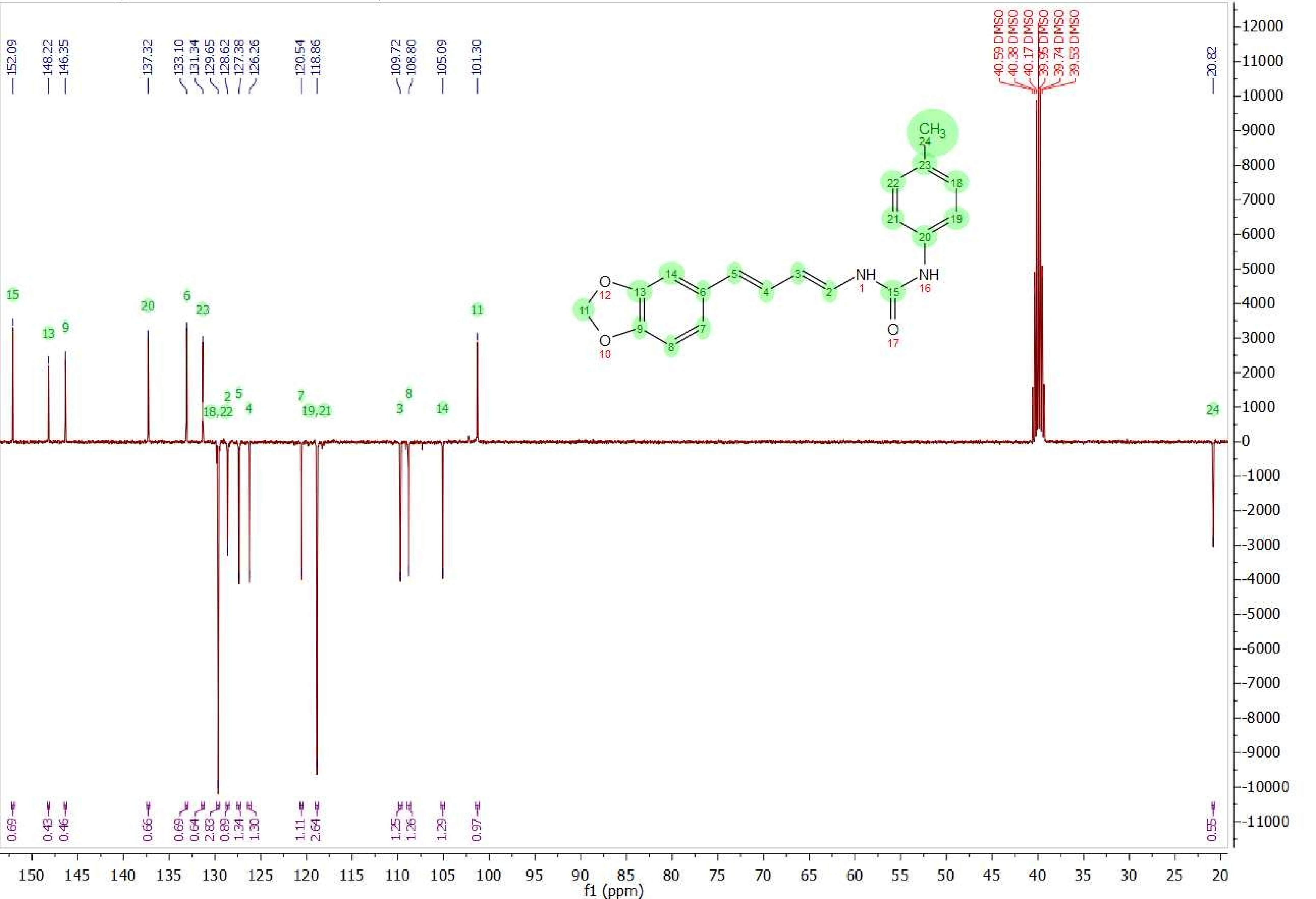

Diaa elemam-2,6.Me.An.Pu-Hnmr-R.10.fid — Diaa elemam-2,6.Me.An.Pu-Hnmr-R

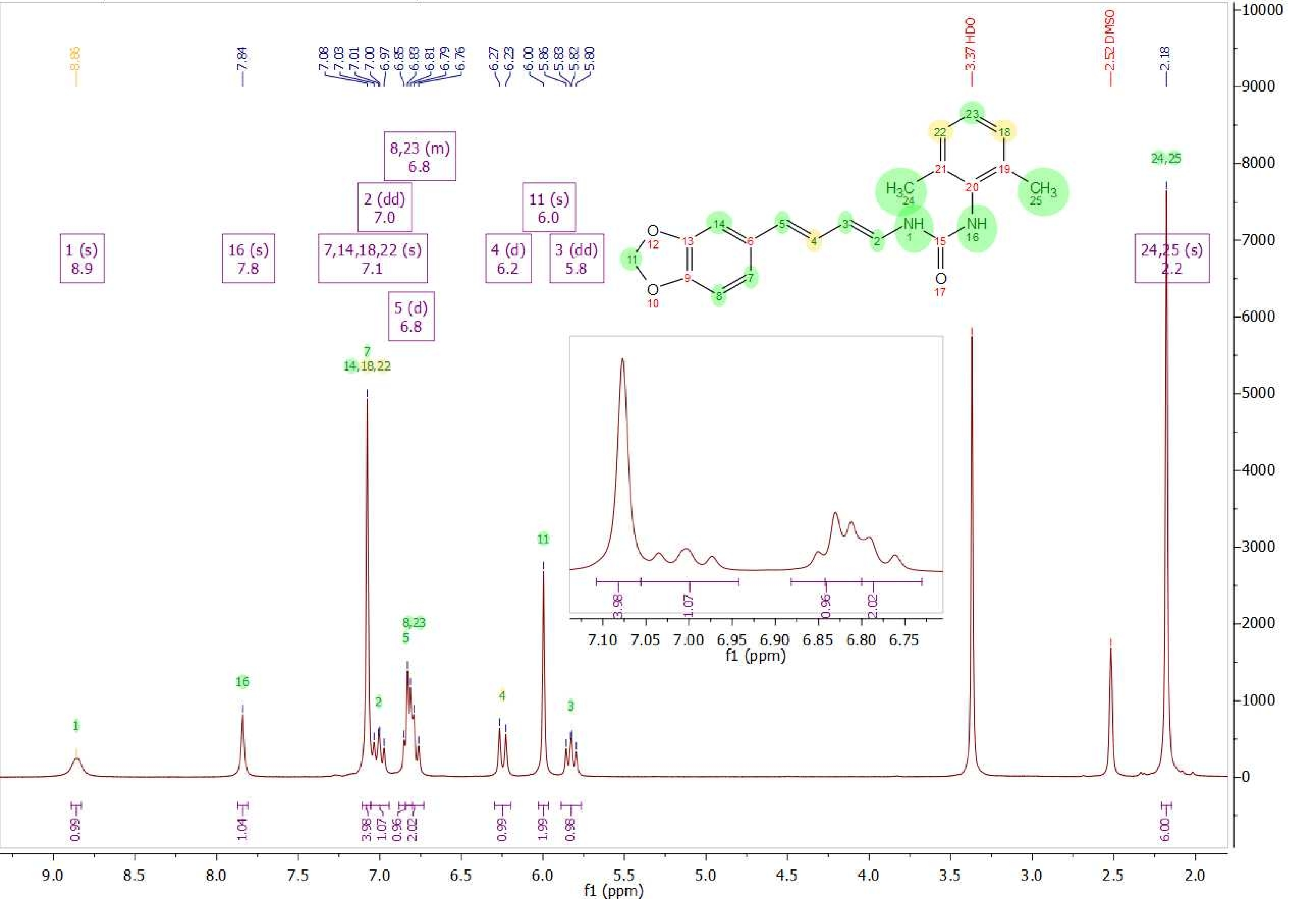

Diaa elemam-2,6.Me.An.Pu-APT-R.10.fid — Diaa elemam-2,6.Me.An.Pu-APT-R

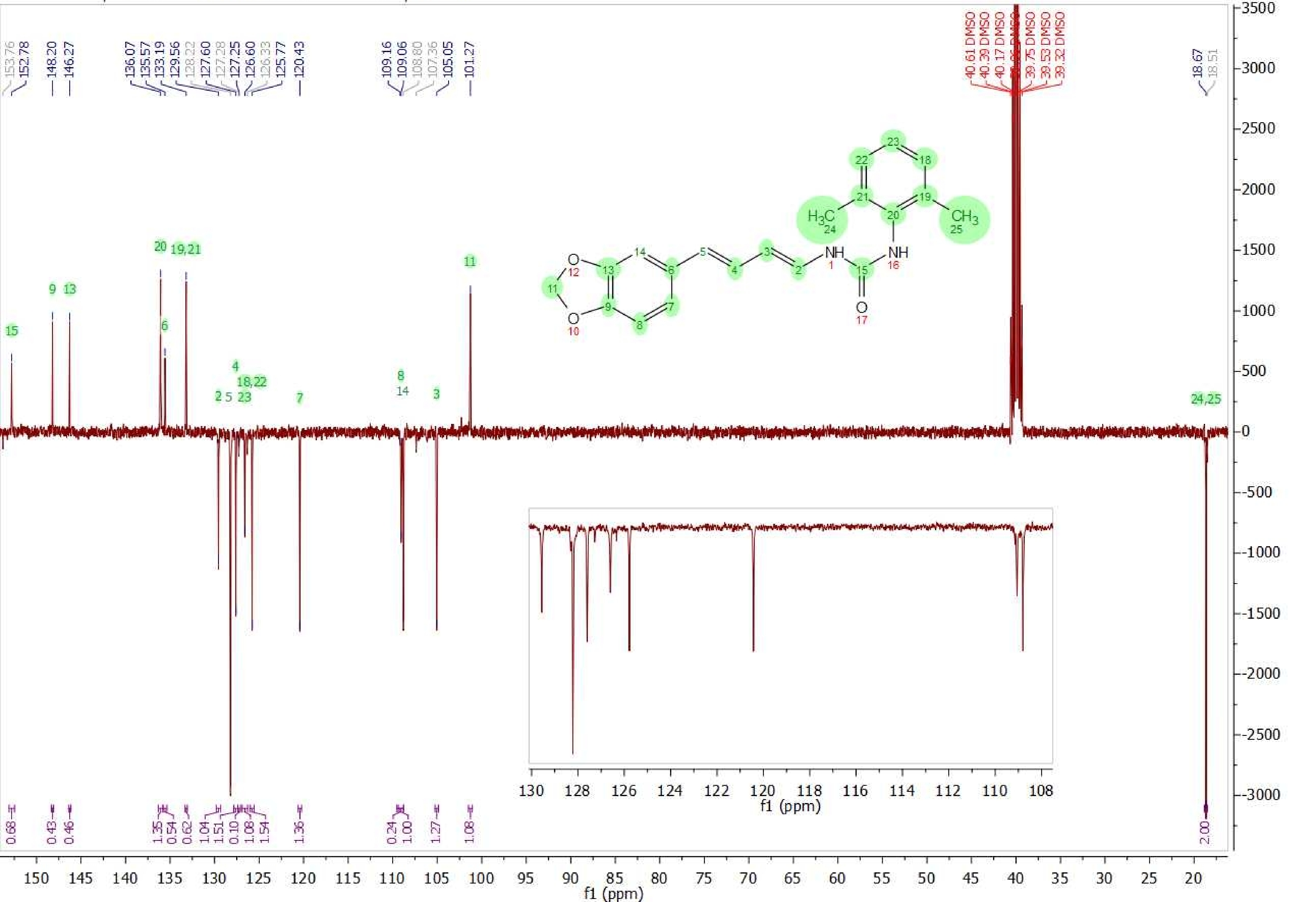

diaa elemam-P.MO.An.pu-Hnmr-ow.10.fid — diaa elemam-P.MO.An.pu-hnmr

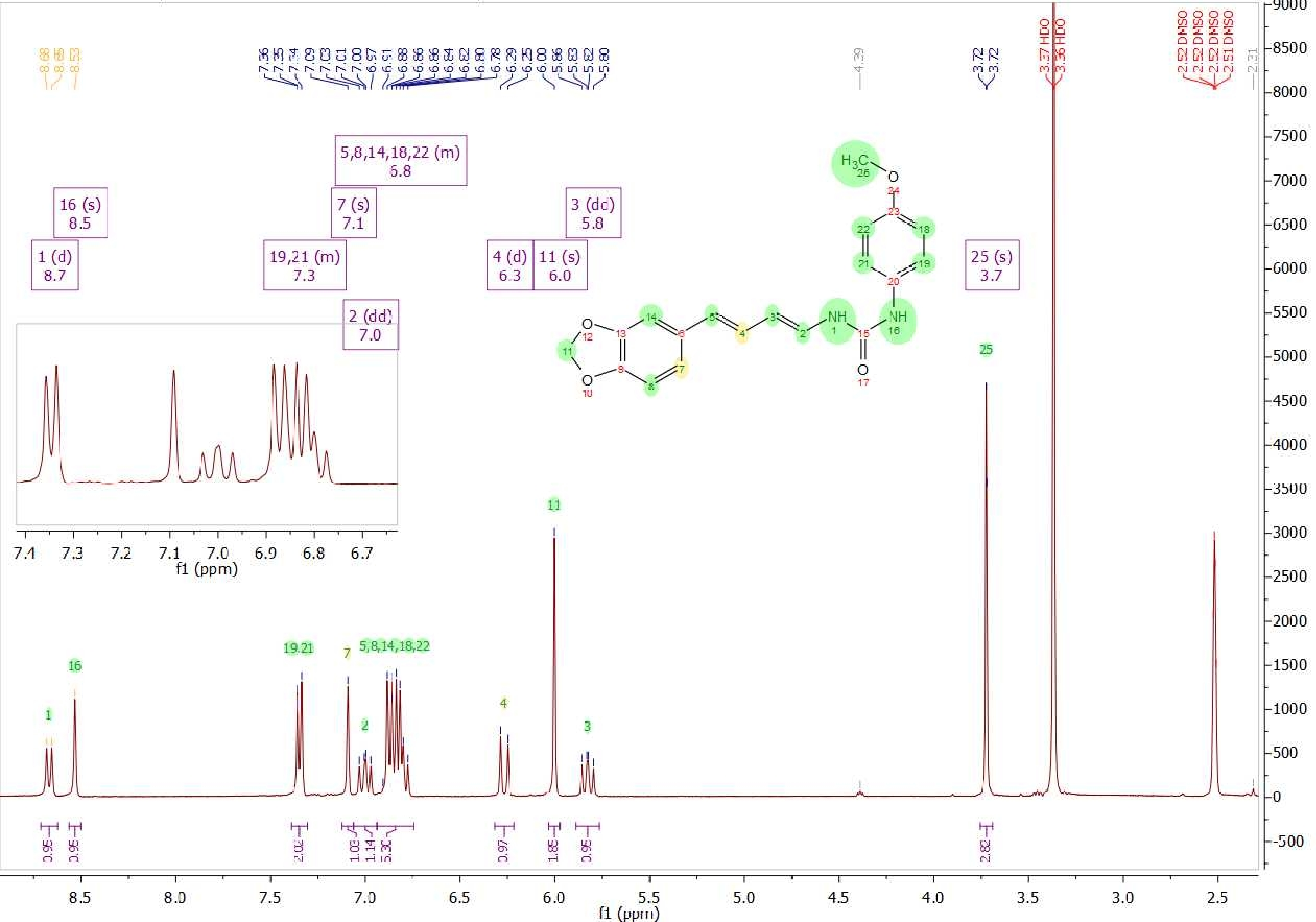

diaa elemam-P.MO.An.pu-ATP-ow.10.fid — diaa elemam-P.MO.An.pu-ATP-ow

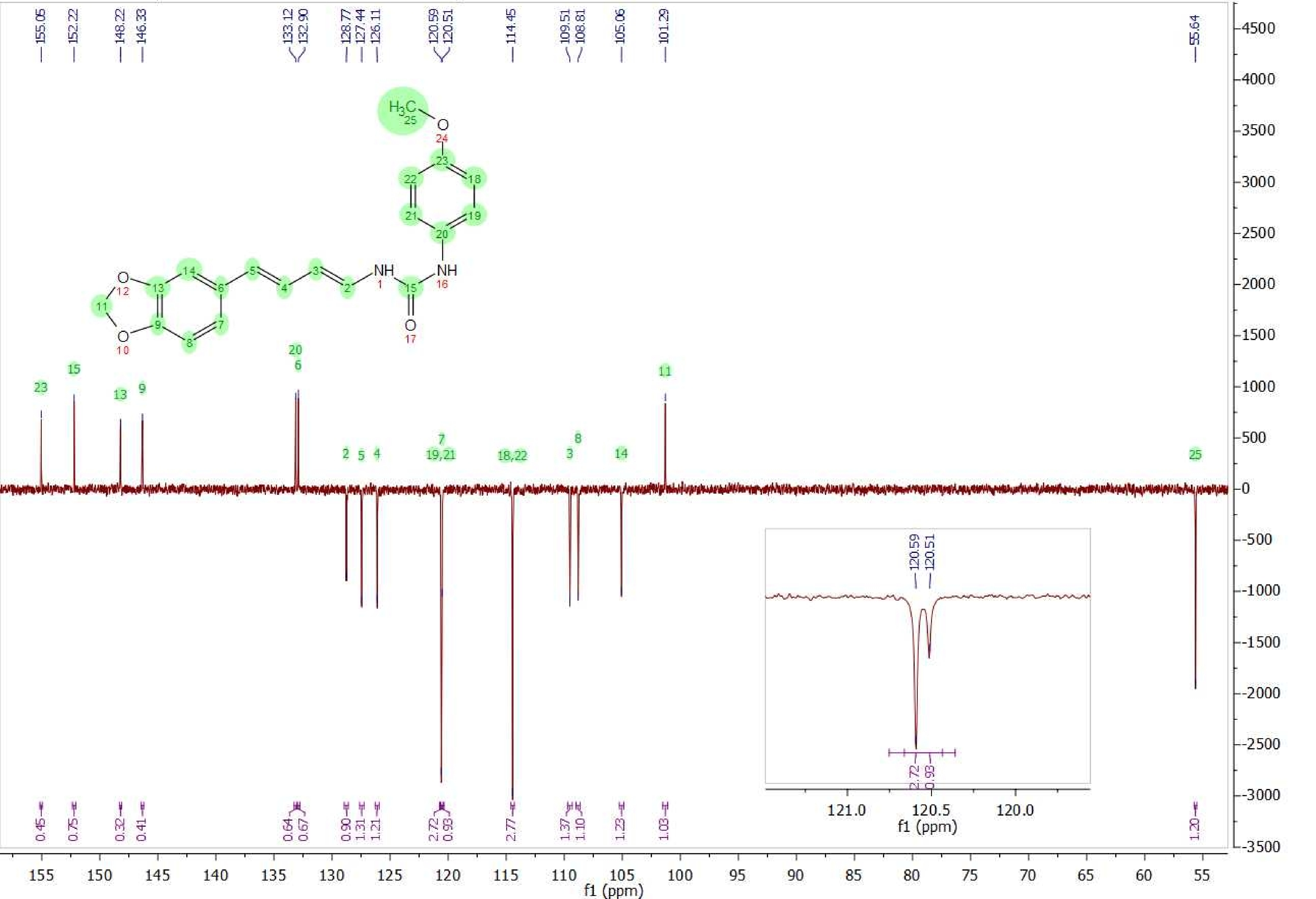

Diaa elemam-4Eto-An.Pu-Hnmr-R.10.fid — Diaa elemam-4Eto-An.Pu-Hnmr-R

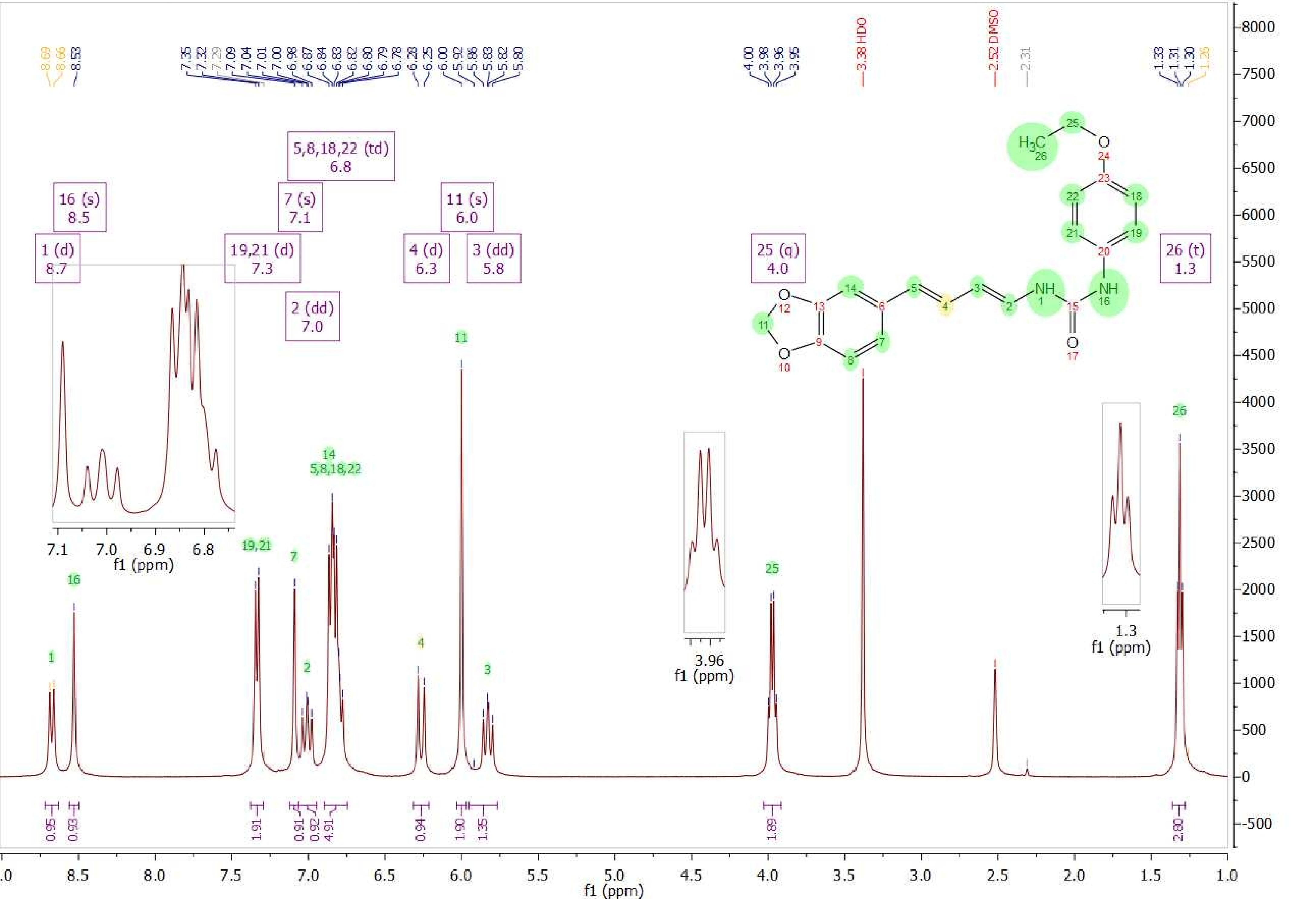

Diaa elemam-4Eto-An.Pu-APT-R.10.fid — Diaa elemam-4Eto-An.Pu-APT-R

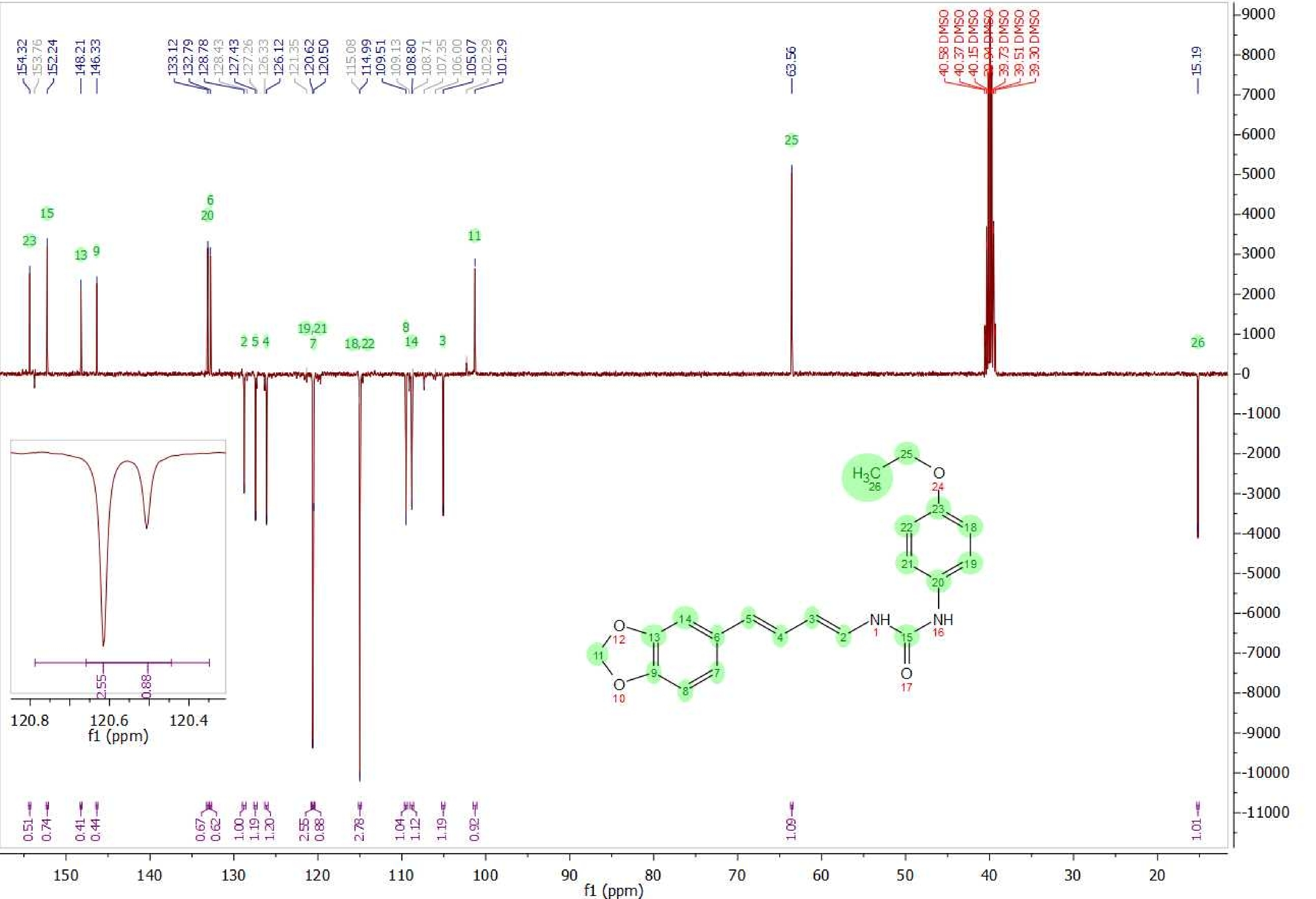

Diaa elemam-3,4Mo.An.Pu-Hnmr-R.10.fid — Diaa elemam-3,4Mo.An.Pu-Hnmr-R

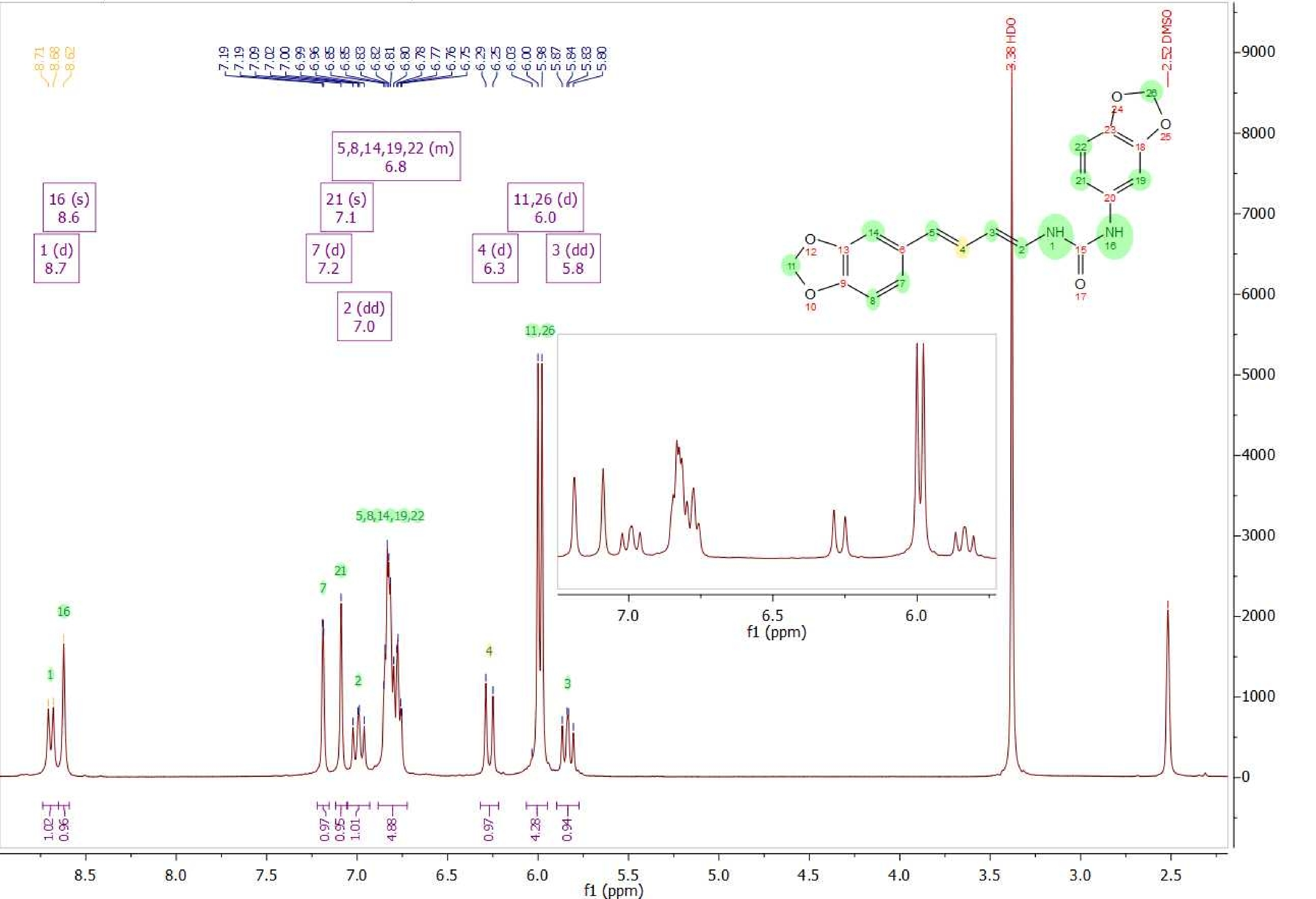

Diaa elemam-3,4Mo.An.Pu-APT-R.10.fid — Diaa elemam-3,4Mo.An.Pu-APT-R

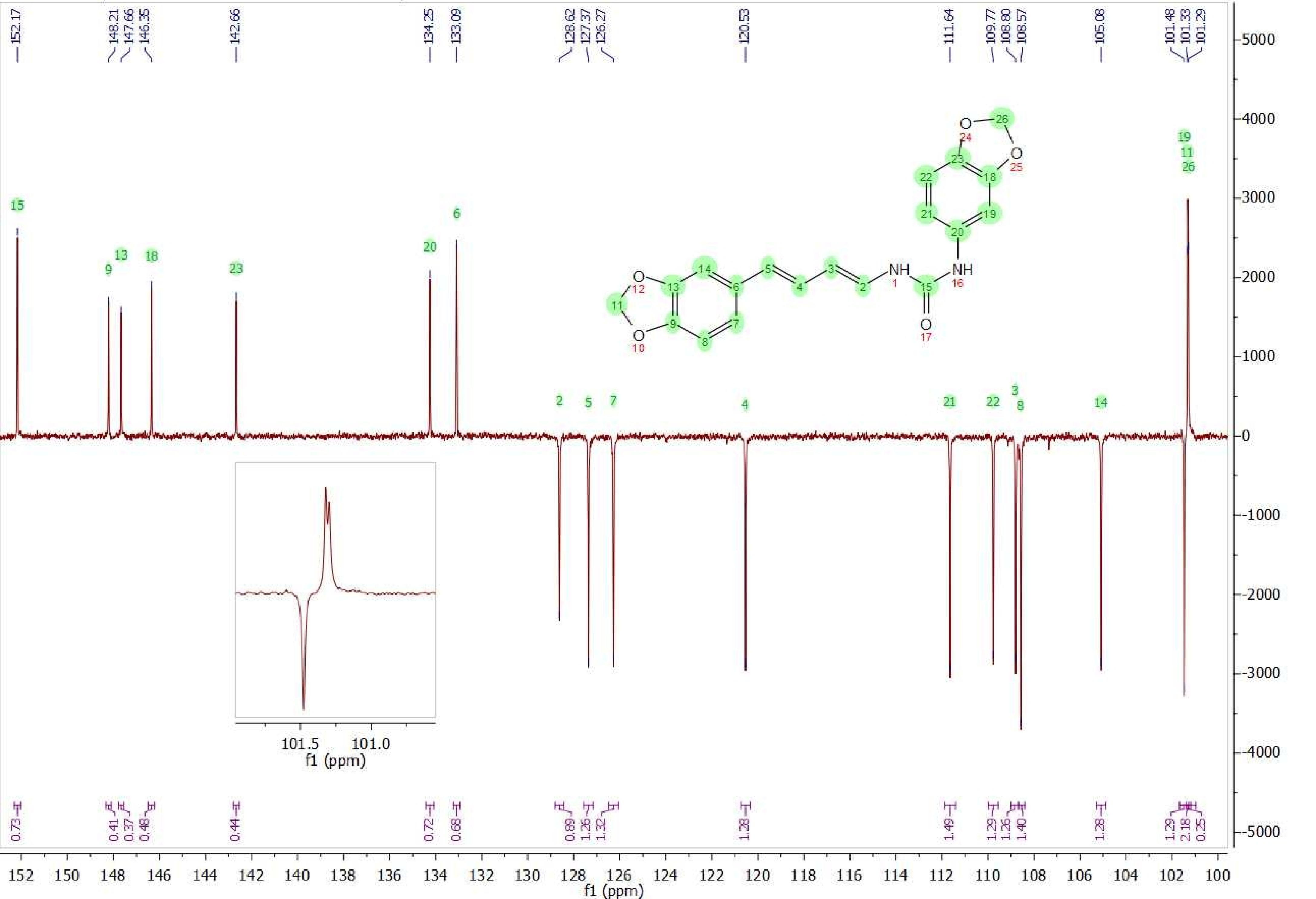

Supplement: Supplemental Material [file IENZ_A_1988944_SM9839.pdf]
